# The coupled reanalysis global meridional overturning circulation imprinted by historic volcanic eruptions

**DOI:** 10.1038/s41467-026-75651-z

**Published:** 2026-07-16

**Authors:** Yingjing Jiang, Shaoqing Zhang, Yang Gao, Lixin Wu, Lv Lu, Zikuan Lin, Wenju Cai, Deliang Chen, L. Ruby Leung, Bin Wang, Xueshun Shen, Mingkui Li, Xiaolin Yu, Xiaopei Lin

**Affiliations:** 1 Institute of Marine Sciences, College of Science, Shantou University, Shantou, China; 2 Key Laboratory of Physical Oceanography, Ministry of Education/Frontiers Science Center for Deep Ocean Multispheres and Earth System/College of Oceanic and Atmospheric Sciences, Ocean University of China, Qingdao, China; 3 Frontiers Science Center for Deep Ocean Multispheres and Earth System/Key Laboratory of Marine Environmental Science and Ecology, Ministry of Education, Ocean University of China, Qingdao, China; 4 Laoshan Laboratory, Qingdao, China; 5 College of Intelligent Systems Science and Engineering, Harbin Engineering University, Harbin, China; 6 Department of Earth System Sciences, Tsinghua University, Beijing, China; 7 Department of Earth Sciences, University of Gothenburg, Gothenburg, Sweden; 8 Atmospheric, Climate, & Earth Sciences Division, Pacific Northwest National Laboratory, Richland, WA USA; 9 Department of Atmospheric Sciences and International Pacific Research Center, University of Hawaii at Manoa, Honolulu, HI USA; 10 The Center of Earth System Modeling and Prediction, CMA, Beijing, China

**Keywords:** Climate and Earth system modelling, Physical oceanography

## Abstract

The global ocean meridional overturning circulation (GMOC) is central for ocean transport and climate variations. However, a comprehensive picture of its historical mean state and variability remains vague due to limitations in modelling and observing systems. Incorporating observations into models offers a viable approach to reconstructing climate history, yet achieving coherent estimates of GMOC has proven challenging due to difficulties in harmonizing ocean stratification. Here, we demonstrate that multiscale data assimilation that integrates atmospheric and oceanic observations into two coupled models in a dynamically consistent way robustly retrieves the past 80-year GMOC. By diagnosing volcanic eruption-induced North Atlantic cooling and surface buoyancy loss, we show historic volcanic eruption events are imprinted in variability of the rebuilt GMOC. The Mt. Pinatubo (1991) volcanic eruption produces a multidecadal characteristic of the overturning evolution by the chain of enhancing diapycnal mixing - strengthening deep convection - mediated by eddy activities.

## Abstract (web summary)

Two converging coupled reanalyses reconstruct ocean overturnings of past 80 years imprinted by historic volcanic events. The 1991 Mt. Pinatubo eruption triggered North Atlantic cooling and deeper convection, driving multidecadal overturning shifts.

## Introduction

In the Earth climate system, the global meridional overturning circulation (GMOC) acts as the primary “conveyor belt” for redistributing heat, freshwater, carbon, and nutrients globally^1–5^ and playing a key role in regulating global climate equilibrium, regional climate patterns, and ocean-atmosphere feedbacks^6–9^ Conceptual box models^10–12^ proxy-based observational reconstructions^13,14^ and multi-complexity Earth system models^15,16^ have collectively advanced mechanistic understanding of meridional overturning circulations. A comprehensive picture on historical GMOC variability is fundamental for assessing climate change risks and improving climate predictions, yet current knowledge on historical GMOC behavior remains limited due to the lack of direct observations—thus necessitating its estimation via advanced data assimilation for coherent model-data integration^17,18^ However, given limited observations in deep oceans, GMOC estimation is highly challenging since it requires a high-order coherence in ocean vertical structure (i.e. stratification)^6,17^ and balance between atmospheric and oceanic variables. Achieving such coherence and balance necessitates the dynamic consistence of atmosphere and ocean as well as multiscale interactions^19,20^ as observations are incorporated into models.

Coupled data assimilation (CDA)^21–23^ incorporates Earth observational information into coupled model dynamics and physics to rebuild an Earth system close to the real world, aiming to produce dynamically-consistent estimates for three-dimensional (3-D) atmospheric and oceanic motions^24,25^ i.e., coupled reanalysis. However, CDA struggles to resolve issues related to insufficient multiscale interactions^26^ and the negative effect of systematic model errors (i.e. model biases) on assimilation, especially in the deep ocean^27^ These challenges lead to divergent estimates of GMOC in existing ocean reanalyses, where mean state and variability often differ substantially^16,28^ The development of CDA over decades^21,24,29–33^ has progressively advanced in mitigating these issues, which is expected to produce useful and reliable estimates of 3-D ocean currents and GMOC^34^.

In this work, we use two CDA systems^32,33^ based on the Community Earth System Model (CESM)^35^ and 2^nd^ generation Coupled Model (CM2)^36^ to assimilate atmospheric and oceanic observations since 1945, creating two coupled reanalyses spanning over the past 80 years (see Methods). After showing the coherent configuration of CDA-estimated atmospheric and oceanic states, through decomposition and composite analyses of different scale modes on geostrophic and ageostrophic components, we demonstrate the observation-convergent mean state and variability of CDA-estimated GMOCs. Through analyzing the multiscale components of the CDA GMOC, we elucidate the physical linkage between the tropical region multiscale activities and high-latitude multidecadal oscillations. We show that by climate signal propagation cross the atmosphere and ocean, the historical GMOC carries the fingerprint of historic volcanic eruptions, and superimposed on the El Chichón (1982) event, the strong Mt. Pinatubo (1991) event triggered the most pronounced basin-wide overturning response over the past 80 years.

## Results

### Robustly-reconstructed GMOC in two coupled reanalyses

**Fig. 1:**
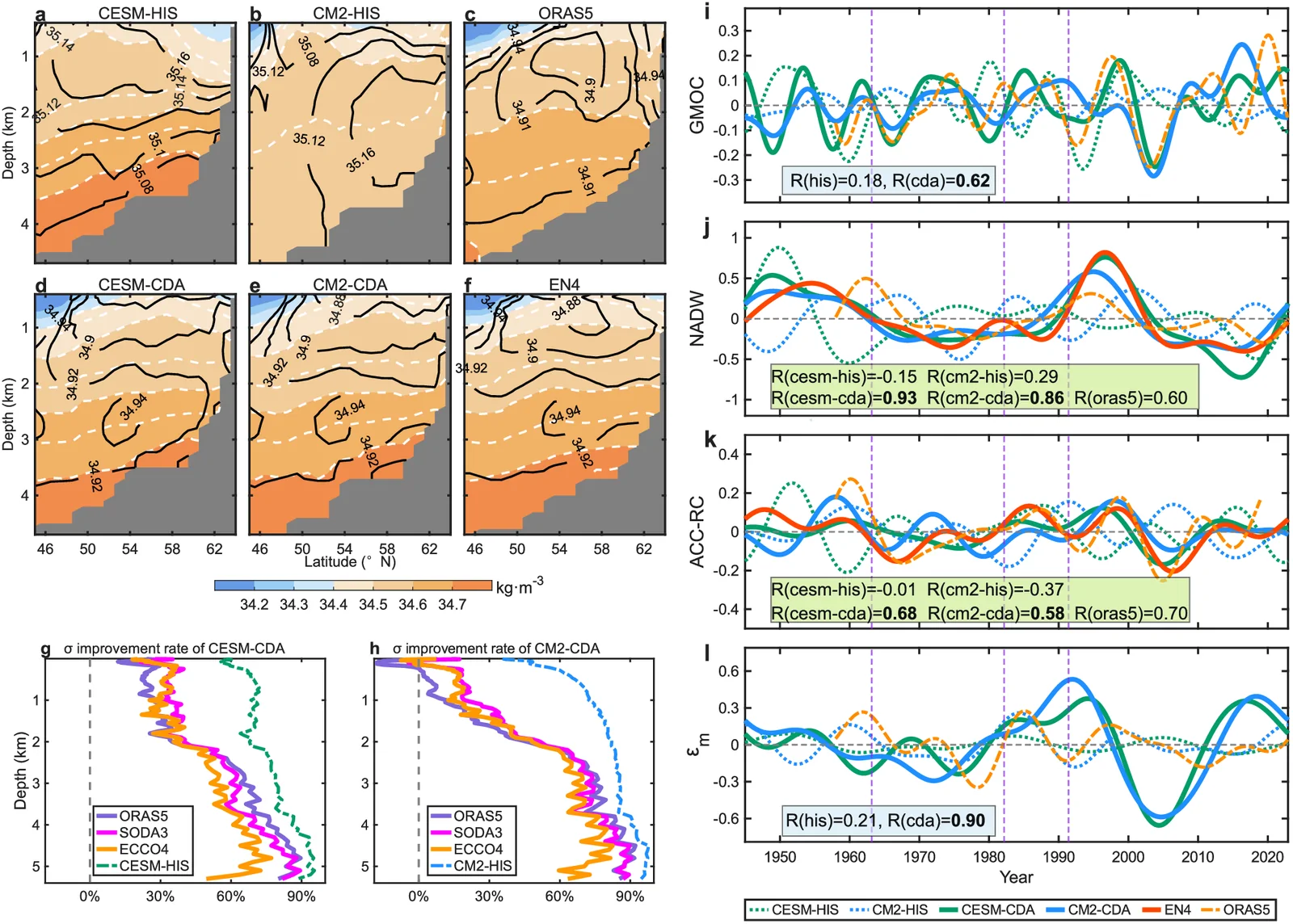
The convergent global meridional overturning circulations (GMOCs) and associated physical modes in two coupled reanalyses. Mean states of potential density distributions in the North Atlantic over the domain of 55°–35° W in CESM-HIS (**a**), CM2-HIS (**b**), ORAS5 (**c**), CESM-CDA (**d**), CM2-CDA (**e**) and observational dataset EN4 (**f**). The shadings with white contours plot potential density referenced to a depth of 1500 m (unit: kg/m^3^). The black contours plot salinity (unit: PSU). Variations of root mean square error (RMSE) reduction rate (denoted as improvement rate) of potential density (σ) with depth in CESM-CDA (**g**) and CM2-CDA (**h**) relative to model simulation as well as other reanalyses, against the observation product WOA18. 5‒20 years filtering of timeseries of indices of GMOC (**i**), North Atlantic Deep Water (NADW) (**j**), Antarctic Circumpolar Current residual circulation (ACC-RC) (**k**) and tropical diffusive mixing (ε_m_) (**l**). The correlation coefficients between two timeseries that need to be analyzed are listed in the small box in each (**i**–**l**). The calculation periods of all correlation coefficients are consistent with the time period of ORAS5. All panels are plotted using MATLAB R2025a.

We evaluate the CDA-estimated atmospheric and oceanic states and calculate the mean GMOC states from two model historical simulations, two CDA estimates and four ocean reanalyses (see Supplementary Texts 1, 2 and Supplementary Figs. 1–18). The two CDA estimates show clear convergence and share considerable similarities with other reanalysis products in GMOC characteristics, and all observation-constrained results feature a much stronger deep GMOC circulation than the free model simulations (Supplementary Fig. 6 and Supplementary Text 2). The multiscale CDA constraints greatly reduce the model simulation biases and produce coherent ocean vertical structure, achieving better mean current velocities than the reference ocean reanalyses (Supplementary Figs. 2, 3). Both CDA estimates align the ocean temperature and salinity structure closely with the observation, producing highly observation-consistent ocean stratification (Supplementary Figs. 11). The convergence of CDA-estimated GMOC mean states can be attributed to the convergent ocean stratification which is consistent with the observation (Fig. 1a–h). Notably, below 2000 m, the reduction of potential density errors of CESM-CDA from CESM-HIS is ~60‒90% (Fig. 1g), and the reduction of CM2-CDA from CM2-HIS even reaches 95% (Fig. 1h). Compared with the reference ocean reanalysis products, the ocean stratification of potential densities in CDA estimates demonstrates significant improvements, especially below 2000 m (Supplementary Figs. 11, 12). As the consequence, the CDA improves the deep ocean (below 2000 m) geostrophic GMOC by 32% (Supplementary Fig. 14a, b) and water volume transport at the 26.5^o^N by 70% (Supplementary Fig. 14c, d) compared to the free model simulation.

In contrast to the free model simulations, variability of the GMOC derived from CDA exhibits excellent convergence and is consistent with the observational results (Supplementary Fig. 17). To understand the mechanism of CDA in reproducing GMOC variability, we decompose the multiscale components of model-simulated and CDA-estimated GMOCs. The GMOC theory^37^ suggests the existence of multiscale processes that balance atmospheric forcing and oceanic response to sustain the GMOC. The balance between the residual of Southern Ocean upwelling minus Northern Hemisphere sinking and the low-latitude diffusive mixing maintains the atmospheric and oceanic meridional circulation (AMMC-GMOC) structure^2,38^ (illustrated in Supplementary Fig. 19). While the North Atlantic Deep Water (NADW) system represents mostly the Northern Hemisphere sinking^39^ the Southern Ocean upwelling is represented by the residual circulation (RC) from the wind-driven Ekman effect and eddy-induced transport on the flanks of submarine ridges along the ACC^40^ i.e., the ACC-RC system (see Supplementary Fig. 20). We calculate the kinetic energy dissipation rate of 30° S–30° N, ε_m_, to examine tropical diffusive mixing effects^41^ (see Methods and Supplementary Fig. 21).

We perform power spectrum analysis and examine band-pass filtered timeseries of GMOC, NADW, ACC-RC, and ε_m_ indices on decadal (5‒20-yr) (Fig. 1i‒l), interannual (2‒5-yr) (Supplementary Fig. 22) and seasonal-to-interannual (6-month‒2-yr) (Supplementary Fig. 23) scales. We find that the anomaly correlation between two model-simulated GMOCs is very low in these three scale bands (0.18, 0.22, and 0.45 respectively), whereas the two CDA-estimated GMOCs are significantly correlated in all scale bands (0.62, 0.73, and 0.89 respectively). This suggests that the model-data integration in CDA does capture multiscale information of the Earth system to some degree. In general, the NADW and ACC-RC decadal modes in all data-constrained products highly correlate with the “observation” in which the correlations of NADW and ACC-RC in ORAS5, CESM-CDA, and CM2-CDA by order are 0.60, 0.93, 0.86 and 0.70, 0.68, 0.58, respectively (Fig. 1j, k). For the seasonal to interannual scales, while the ACC-RC highly correlates with the “observation” (the correlations in ORAS5, CESM-CDA, and CM2-CDA by order are 0.65, 0.73, 0.79 in the interannual band and 0.82, 0.86, 0.73 in the seasonal-to-interannual band), the NADW has however no significant correlation with the “observation” in all data-constrained products (Supplementary Figs. 22b, 23b). This means that while the CDA is able to coherently integrate data into coupled models and rebuild the 3-D geo-fluids, it is also constrained by the model’s deficiency. Because of absent mesoscales in the 100 km coarse resolution assimilation models used in this study, it’s very difficult for the CDA to retrieve high-frequency signals of NADW, for which mesoscale eddies play an important role^42^

We also find that the GMOC’s decadal oscillations starting from the early 1980s has a significant fingerprint on the decadal modes of NADW, ACC-RC, and ε_m_ in both CDA estimates (Fig. 1j‒l). In the seasonal to interannual scales, the GMOC significantly correlates with the corresponding ACC-RC mode (Supplementary Figs. 22c, 23c). At the interannual band, the correlations of GMOC with ACC-RC in ORAS5, CESM-CDA, and CM2-CDA are 0.61, 0.74, and 0.69, respectively, and at the seasonal-to-interannual band, the correlations are 0.33, 0.65, 0.37 respectively, whereas NADW and ε_m_ have no robust correlation with GMOC in these bands. This phenomenon could be attributed to the multiscale characteristics of ACC-RC as a strongly atmosphere-ocean coupled component of the Earth system, whereas the seasonal to interannual scale NADW and ε_m_ modes strongly rely on oceanic mesoscale activities and associated air-sea interactions^43^ which are unresolved in the models used in this study.

More evaluations of atmospheric and oceanic states in Supplementary Text 1 and GMOC mean state and variability in Supplementary Text 2 for two coupled reanalyses strongly support the results above.

### Physical modes in historical GMOC variability

**Fig. 2:**
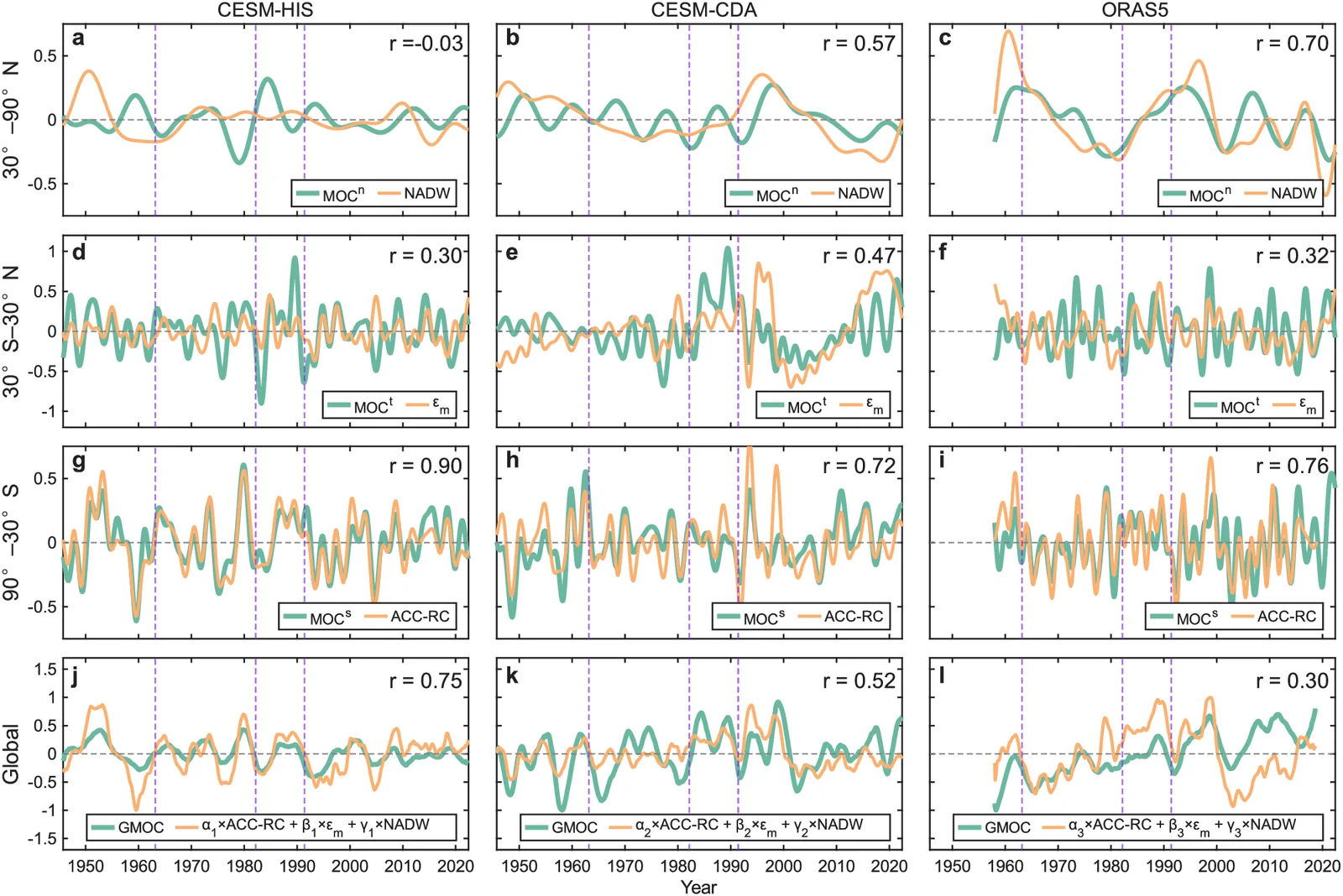
Composited GMOC with its physical modes in CESM model simulation and coupled reanalysis. The first principal component (PC1) derived from empirical orthogonal function (EOF) decomposition of GMOC over the area north of 30° N (denoted as MOC^n^) and 5–30-yr filtered timeseries of normalized North Atlantic Deep Water (NADW) in CESM-HIS (**a**), CESM-CDA (**b**), and ORAS5 (**c**). **d**–**f** Same as (**a**–**c**), but for PC1 of GMOC over the area of 30° S–30° N (denoted as MOC^t^) and 6-month–20-yr filtered kinetic energy dissipation rate (ε_m_). **g**–**i** Same as (**a**–**c**), but for PC1 of GMOC over the area south of 65° S (denoted as MOC^s^) and the 1–20-yr filtered Antarctic Circumpolar Current residual circulation (ACC-RC). **j**–**l** Timeseries of the 24-month running mean GMOC index and linear combination of ACC-RC, ε_m_, and NADW with coefficients α, β, and γ, respectively. The parameters α, β, and γ are the linear regression coefficients between the GMOC indices and ACC-RC, ε_m_, NADW indices, respectively, as [α_1_, β_1_, γ_1_] = [0.64, −0.05, 0.24], [α_2_, β_2_, γ_2_] = [0.46, 0.12, 0.07], and [α_3_, β_3_, γ_3_] = [0.31, −0.01, 0.08]. Generated by MATLAB R2025a.

To better understand the key processes that maintain GMOC, we perform a composite analysis of GMOC variability in terms of the NADW, ACC-RC, and ε_m_ (Fig. 2 and Supplementary Fig. 25). We first calculate the 1st principal component of GMOC stream functions over regions in the north of 30° N, between 30° S and 30° N, as well as in the south of 30° S (denoted as MOC^n^, MOC^t^ and MOC^s^). We find that the MOC^n^, MOC^t^, and MOC^s^ generally correlate with the NADW, ε_m_ and ACC-RC indices well respectively. Variability of the GMOC is largely accounted for by the linear combination of ACC-RC, NADW, and ε_m_ in both free model simulations and data-constrained products, and such fit is a little better in free model simulations. The mean correlation between the original and synthesized GMOC indices is 0.69 for the two free model cases and 0.45 for the three data-constrained cases. This reflects how well models tend to express idealized relationships between variables, while data constraints inevitably introduce some imbalances as model misfits are corrected by data. However, the correlation in the two CDA estimates is much higher (0.53) than in the ORAS5 ocean reanalysis (0.30), suggesting that the approach of CDA data constraint may help reduce imbalances introduced during model-data integration by the atmosphere-ocean coupling mechanism.

With the exception of the CESM-HIS case, in all other instances of free model simulations and data-constrained products, the PC1s of MOC^n^, MOC^t^, and MOC^s^ are substantially correlated with the NADW, ε_m_, and ACC-RC indices, respectively. The low correlation between the MOC^n^ and NADW in CESM-HIS may be attributed to the specific model deficiency in the North Atlantic of CESM^42^ This deficiency is largely rectified by the CDA data constraints, as can be seen by comparing Supplementary Fig. 25a, b. In all data-constrained products, the correlation between the MOC^s^ and ACC-RC is highest, while the correlation between the MOC^t^ and ε_m_ is lowest; the correlation between the MOC^n^ and NADW lies between them. The mean correlations of these three data-constrained products are 0.73 between MOC^s^ and ACC-RC, 0.52 between MOC^n^ and NADW and 0.53 between MOC^t^ and ε_m_, respectively. All these correlations far exceed the 99% significance level (which is ~0.12).

Improved GMOC variability in CDA arises from better simulation of its dominant drivers: Northern–Tropical Ocean diapycnal mixing transport (with improved correlations by 63% and 73% versus free model simulation) and Southern Ocean wind‑driven transport (correlation improved by 71%) (see Supplementary Fig. 26 and Supplementary Text 3c). Specifically, while getting better GMOC’s geostrophic component by ocean temperature and salinity assimilation, CDA improves wind-driven transport via surface pressure to constrain wind systems, and improves the representation of diapycnal mixing by producing a more realistic ocean stratification through the CDA including deep‑ocean climatological restoration.

More analyses on physics of GMOC variability can be seen in Supplementary Text 3, which solidly support the analysis above.

### Fingerprints of historic volcanic eruptions on GMOC

**Fig. 3:**
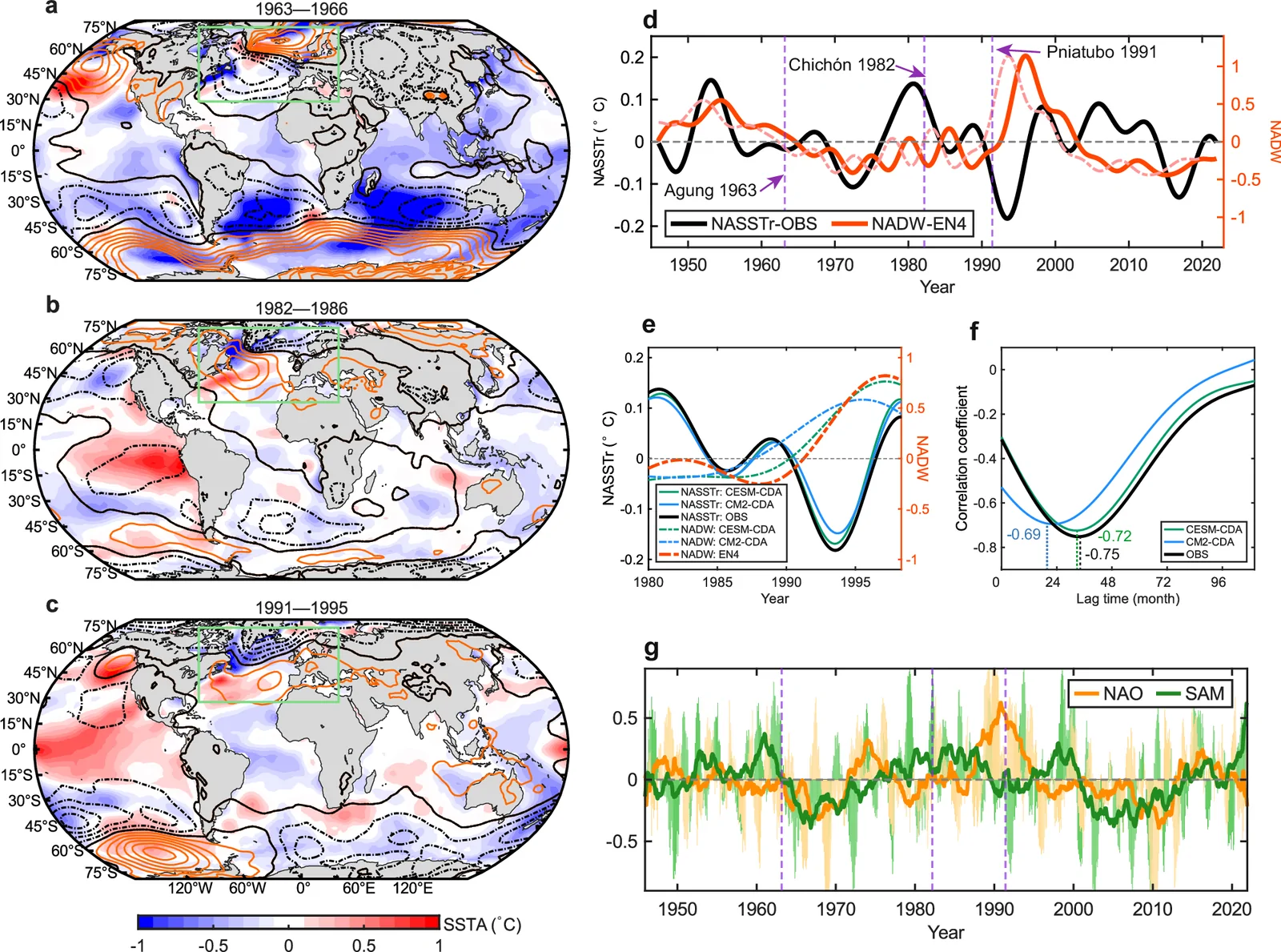
The response of NADW on major volcanic eruptions in the 2^nd^ half of 20^th^ Century. Distribution of OISST sea surface temperature anomaly (SSTA) (shaded) and ERA5 sea level pressure anomaly (SLPA) (contour) during the periods of 1963–1966 (**a**), 1982–1986 (**b**) and 1991–1995 (**c**). These three global SST cooling periods respectively correspond to the three major volcanic eruption events, Mt. Agung (1963), Mt. El Chichón (1982), and Mt. Pinatubo (1991)^44^ in the second half of the 20th Century. **d** Timeseries of 5–20-yr band-pass filtering NASSTr (see Supplementary Text 3d for the definition) in the observation (OBS) (solid-black) (°C) and 2–20-yr band-pass filtering normalized North Atlantic Deep Water (NADW) in EN4 (solid-red). The times of three volcanic eruptions events are marked by light-purple vertical lines. The dashed-light-red curve is a 28-month backward shifted version of the red-solid curve. **e** 5–20-yr band-pass filtering NASSTr and NADW during 1980–2000. **f** Lag correlation coefficients between NADW and NASSTr in (**e**) (the number corresponds to the value at the same color point). **g** The 5-year (dark-color) and 1-year (light-color) running mean timeseries of NAO (North Atlantic Oscillation) (orange) and SAM (Southern Annular Mode) (green) indices, based on ECMWF ERA5 reanalysis. Generated by MATLAB R2025a.

Previous model studies have shown that if superimposed on a cold phase of surface ocean internal variability, the cooling effect of natural aerosols caused by a volcanic eruption can greatly enhance and extend the cold abnormality of sea surface temperature (SST) in a global scope^44^ Two CDA estimates produce nearly identical variability of the global mean SST residual (GMSSTr) (a quadratic fit of GMSST being removed) on Mt. Agung (1963), Mt. El Chichón (1982) and Mt. Pinatubo (1991) events (Supplementary Fig. 27a), which shows the same features of observed SST as these volcanic eruptions been detected^44,45^ Here, we detect the consequence of such cold SST anomalies caused by these historic volcanic eruptions impacting on NADW, so as on GMOC, ACC-RC, and ε_m_. We find that the SST anomaly (SSTA) distributions produced by the Chichón-1982 and Pinatubo-1991 events are very similar, whereas the distribution produced by the Agung-1963 event is very different (Fig. 3a‒c). While the Agung-1963 produced cold SSTAs in a large area over the Southern Ocean and warm SSTAs in the North Atlantic, the Chichón-1982 and Pinatubo-1991 mainly produced cold SSTAs in the North Atlantic. To detect possible linkage between the volcanic eruption-caused SSTA and the signal of NADW, we examine the timeseries of North Atlantic SST residual (NASSTr) and NADW indices (Fig. 3d). We find that the variation of NADW has generally an opposite phase with the NASSTr by a roughly 2‒3-yr lag (Fig. 3e, f), reflecting a speed of a couple of meters per day for local water vertical transport in the North Atlantic^46^ Due to enhancement by the cooling effects of the Chichón and Pinatubo events, the variation of NASSTr appears a very strong intradecadal cold phase from the early 1980s to the end of 1990s, with the bottom in 1993‒1994. The surface cold water corresponds to the strong NADW phase over 1990‒2002, with the peak in 1996‒1997.

The AMMC anomalies (departure from time mean) caused by the Agung and Chichón/Pinatubo events are quite different (Supplementary Fig. 29). This suggests that the atmospheric background wind systems during the Agung and Chichón/Pinatubo are quite different, resulting in different aerosol distributions that drive different anomalous atmospheric circulations^47^ Indeed, the Pinatubo-1991 event coincided with a strongly positive North Atlantic Oscillation (NAO) phase (Fig. 3g), a condition that strengthens the westerly jet stream and promotes poleward heat transport. Within this dynamical setting, the volcanic eruption-induced North Atlantic cooling created favorable conditions for enhanced meridional transport and intensified deep convection. In contrast, the Agung-1963 and Chichón-1982 events occurred during a negative and a near-neutral NAO phase, respectively. Consistent with these background states, the associated NASSTr and NADW formation were substantially weaker. Furthermore, the Agung-1963 event occurred during a negative phase of the Southern Annular Mode (SAM). This background state weakened Southern Ocean wind stress, subsequently reducing the transport of the Antarctic Circumpolar Current (Fig. 1k) and the northward meridional heat/salt flux in the South Atlantic. The resulting attenuation of positive salinity anomaly in the NADW formation region ultimately suppressed local density-driven downwelling (Figs. 1j and 3d).

More analyses of GMOC’s fingerprints on historic volcanic events can be found in Supplementary Text 4, which strongly support the conclusions gained above.

### Responses of North Atlantic overturning on volcanic aerosols

**Fig. 4:**
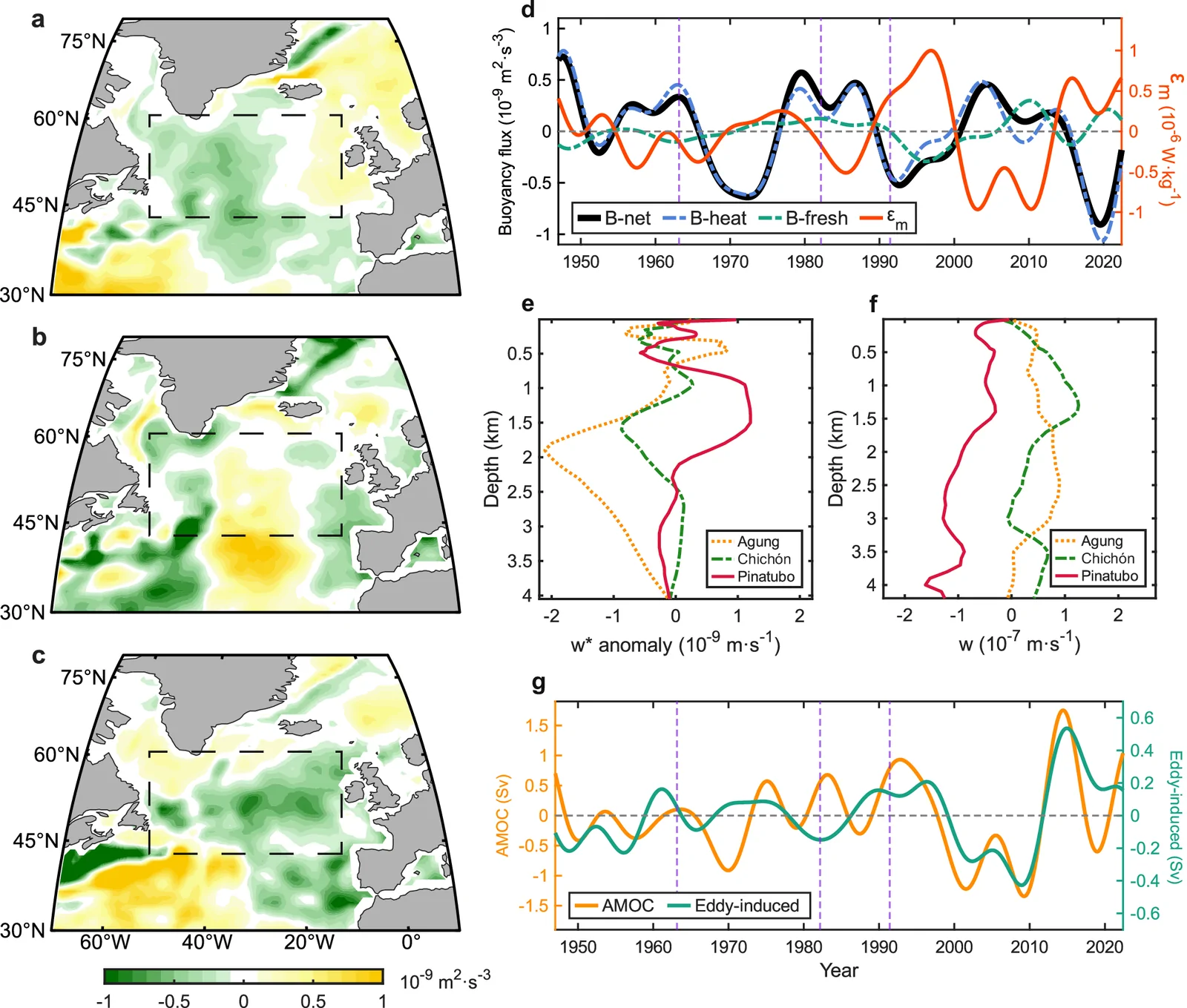
Surface buoyancy flux, diapycnal mixing, and AMOC responses to major volcanic eruptions in the North Atlantic. **a**–**c** Spatial distributions in the North Atlantic of 5-year (2-year) mean surface buoyancy flux difference (10^−9^ m^2^/s^3^) after and before the Agung (1963) (5-year mean) (**a**), Chichón (1982) (2-year mean) (**b**) and Pinatubo (1991) (5-year mean) (**c**) volcanic eruptions, based on the mean of two CDAs. The dashed box denotes the high-latitude key region for AMOC formation, used to define indices in (**d**–**g**). **d** Timeseries of 5–20-yr band-pass filtering surface buoyancy flux indices, the total B-net and its two components, thermal B-heat and freshwater B-fresh, as well as diapycnal mixing rate (i.e. ε_m_ in the high-latitude North Atlantic). All indices are based on the CDA mean. **e** 5-year time mean vertical profiles of diapycnal velocity anomaly (w*, 10^−9^ m/s) in the high-latitude North Atlantic after volcanic eruptions, based on CESM-CDA estimation. **f** Same as (**e**) but for vertical velocity (w, 10^−7^ m⋅s^−1^). **g** Timeseries of 5–20-yr band-pass filtering AMOC transport (Sv) and eddy-induced transport in the high-latitude North Atlantic, from CESM-CDA estimation. Vertical dashed lines in (**d**) & (**g**) mark the eruption date (1963-03, 1982-03, 1991-06). Generated by MATLAB R2025a.

Based on distinct climatic backgrounds, the cooling effect of volcanic aerosols in different volcanic eruptions can produce different atmospheric circulation patterns^47^ which in turn induce variable perturbations to NADW formation. Figure 4a–c shows the composite 5‑year (2-year) mean surface buoyancy flux over the North Atlantic, before and after the Agung-1963 (5-year mean), Chichón-1982 (2-year mean) and Pinatubo-1991 (5-year mean) events. A pronounced buoyancy loss followed the Pinatubo-1991, consistent with enhanced seawater sinking, whereas the Agung-1963 and Chichón-1982 produced only a weak flux anomaly. Throughout the record, surface heat flux dominates the total buoyancy flux. After 1991, however, the freshwater flux component shows a clear decline—a small but consequential anomaly for the circulation (Fig. 4d). Persistent negative surface buoyancy flux anomalies are recorded from 1991 to 2000, which create favorable conditions for the intensification of deep convection and diapycnal mixing in the high-latitude North Atlantic over this period. Notably, combined with deep-ocean restoring, CDA improves the representation of diapycnal mixing processes (Supplementary Fig. 26a) and underpins realistic simulation of volcanic perturbations in NADW formation.

Anomalies of diapycnal mixing velocity (see Methods) in follow-up 5 years after these volcanic eruptions are presented in Fig. 4e. The Pinatubo-1991 exhibits the strongest positive anomalies in the surface layer, intensifying by depth and diminishing at ~1 km, which is a signature indicating destabilized water column that favors the development of deep convection. The Agung-1963 and Chichón-1982 exhibit weaker and shallower density‑gradient anomalies, aligning with their subdued buoyancy‑flux responses. A complementary analysis of vertical velocity (w) anomalies (Fig. 4f) corroborates this pattern: strong downward motions extending to ~4 km depth are induced by the Pinatubo-1991, whereas the Agung-1963 and Chichón-1982 events give rise to weaker and shallower vertical motions. This message further confirms the mechanistic linkage between volcanic cooling, buoyancy loss, and intensified deep sinking. Further analysis of the CDA results reveals pronounced AMOC transport anomalies in the high-latitude North Atlantic following all three volcanic eruptions (Fig. 4g). The AMOC response was the clearest after the Pinatubo-1991 event: it initially strengthened, peaked, and then gradually declined—a evolution that closely follows changes in NADW formation. The overturning driven by eddies tracks this evolution and contributes about 19% of the total MOC variability. These results highlight how eddy processes mediate the chain of events from volcanic forcing to changes in surface buoyancy, diapycnal mixing, and deep convection.

## Discussions

By analyzing multiscale characteristics of GMOC and its components such as NADW, ACC-RC, and decadal tropical kinetic energy dissipation rates in two coupled reanalyses, we find that strong volcanic eruptions can be imprinted distinctly on GMOC variability. A clear example is the Mt. Pinatubo (1991) eruption, which induced pronounced North Atlantic cooling and surface buoyancy loss, thereby enhancing diapycnal mixing and strengthening deep convection that is mediated by eddy activities, leading to a multidecadal overturning characteristic from strengthening to weakening. While the GMOC’s fingerprints on pronounced historic events have been identified, accurately reconstructed 3-D ocean currents and GMOC are expected to further provide key insights into the sources of predictability for extreme climate phenomena associated with internal modes in the coupled system. With the multiscale representation provided by coupled reanalysis data, advanced artificial intelligence and machine-learning forecasting methods^48,49^ can be further developed to enhance seamless prediction capabilities. Then, coupled reanalyses with models that can resolve tropical cyclones, ocean mesoscale eddies, clouds, and sub-mesoscales^50^ as well as multiscale sea-ice processes^51^ are essential to boost the understanding of Earth system’s multiscale processes. In particular, sea-ice genesis and melting are inherently multiscale phenomena, encompassing scales ranging from millimeters to thousands of kilometers, each playing a unique role^51^ and requiring unique numerically processing in both thermodynamic processes and rheological dynamics^51,52^ Given the booming availability of multisource multisphere data, refined coupled reanalysis that incorporates such data into updated high-resolution models with deep machine-learning schemes shall provide a plausible way to break such bottlenecks.

## Methods

### Coupled data assimilation systems

#### Two coupled models

The CESM^35^ and CM2^36^ employed the historical simulation period from 1945 to 2006 and the Representative Concentration Pathway 4.5 (RCP45) scenario period from 2007 to the present. For CESM, the atmospheric component (Community Atmosphere Model version 5, CAM5) employed a spectral-element dynamic core (“ne30g16” configuration) with ~1° × 1° horizontal resolution near the equator, and 26 vertical levels; the oceanic component (Parallel Ocean Program version 2, POP2) is configured with a horizontal resolution of ~1° × 1° and 60 vertical levels, with 15 levels of 10-m thickness in the upper 150 m. For CM2, the atmospheric component (Atmosphere Model version 2.1, AM2.1) employed a finite-volume dynamic core with a horizontal resolution of 2° latitude × 2.5° longitude and 24 vertical levels; the oceanic component (Modular Ocean Model version 4, MOM4) is configured with 50 vertical levels (22 of which are 10-m thick in the upper 220 m) and a 1° × 1° horizontal B-grid resolution, telescoping to 1/3° meridional spacing near the equator.

#### Multiscale data assimilation algorithm

A multi-timescale, high-efficiency approximate Ensemble Kalman Filter^31^ combined with a deep-ocean bias relaxation scheme^18^ is implemented into both models to establish two CDA systems. This CDA algorithm uses the single-model state timeseries to construct stationary, low-frequency, and high-frequency filters, markedly reducing the consumption of computational resources^32,33^ The algorithm consists of the following two steps:

1 $$\Delta {y}^{o,\left(\tau \right)}=\frac{\frac{1}{{\left({\sigma }^{\left(\tau \right)}\right)}^{2}}{y}_{f}^{ \, p}+\frac{1}{{\left({\sigma }^{\left(o\right)}\right)}^{2}}{y}^{o}}{\frac{1}{{\left({\sigma }^{\left(\tau \right)}\right)}^{2}}+\frac{1}{{\left({\sigma }^{\left(o\right)}\right)}^{2}}}-{y}_{f}^{ \, p},\tau=1,\varGamma \, \left(\varGamma=3\,{in}\,{this}\,{study}\right),$$

2 $$\Delta {x}^{u}=\mathop{\sum }\limits_{\tau=1}^{\Gamma }{\alpha }^{\left(\tau \right)}\frac{{{\mathrm{cov}}}\left(\Delta {x}^{\left(\tau \right)},\Delta {y}^{\left(\tau \right)}\right)}{{\left({\sigma }^{\left(\tau \right)}\right)}^{2}}\cdot \Delta {y}^{o,\left(\tau \right)},\tau=1,\varGamma \, \left(\varGamma=3\,{in}\,{this}\,{study}\right).$$

Here, $\Delta {y}^{o,(\tau )}$ represents the observational increment, ${y}_{f}^{ \, p}$ is the “prior” model-estimated value at the observation location, ${\sigma }^{(\tau )}$ is the standard deviation of the multi-timescale (three timescales: stationary, low-frequency, and high-frequency in this case) ensemble, ${\sigma }^{(o)}$ is the standard deviation of the observation, ${y}^{o}$ is the observation, $\Delta {x}^{u}$ is the observation increment on the model grid after projection, ${\mathrm{cov}}(\Delta {x}^{\left(\tau \right)},\Delta {y}^{\left(\tau \right)})$ is the covariance relationship between the model value at the model grid and the estimated model value at the observation location of each timescale filtering scheme, and ${\alpha }^{(\tau )}$ represents the adjustable weight coefficients in the combination of filters.

In addition, given the scarcity of deep-ocean observations, an ocean model bias relaxing scheme^18^ is used during CDA to control deep-ocean model bias and achieve coherent ocean stratification^8,28^ In this scheme, climatological temperature and salinity data are restored into the model space with depth-dependent restoration strength. Specifically, the upper ocean is primarily constrained by observations, while the deep ocean is relaxed towards the climatology of the World Ocean Atlas (WOA)^53,54^ It is worth noting that neither the CDA alone nor the deep‑ocean restoring alone suffices^18^ a balanced and coherent ocean stratification is achieved only when the deep biases are constrained by climatological relaxation while the upper ocean is simultaneously constrained by observations.

#### Coupled data assimilation setting

Ocean observations—including sea surface temperatures (SSTs) from the Hadley Centre Sea Ice and Sea Surface Temperature Dataset (HadISST) and Optimum Interpolation SST (OISST), as well as in-situ ocean temperature and salinity profiles since 1945—are assimilated into the CDA systems. To simplify the CDA framework for climate reanalysis, only gridded surface pressure (P_s_) data from the fifth-generation ECMWF reanalysis (ERA5) atmospheric reanalysis products^55^ are assimilated as the atmospheric “observations” (see Supplementary Text 1c), which is in contrast to the coupled reanalysis CERA^23^ The P_s_ data effectively represent all other atmospheric information by capturing whole-column atmospheric mass variation^56^ The assimilation of P_s_ increments uses an inverted projection method of the vertical integral, effectively extracting observational information into the models^56,57^ The CESM-CDA and CM2-CDA systems are initialized on 1 January 1945 from their historical runs (branched from millennial piControl simulations), where the GMOC had reached a model equilibrium. For such initialized systems, the data assimilation (DA) spin-up proceeds far quicker than free model integration due to the direct imposition of observational constraints (see Supplementary Fig. 5 and Supplementary Text 1d)^21,58^ These systems completed their coupled reanalysis to the present, using historical radiative forcings, and followed the RCP45 scenario setting after 2007 (see Supplementary Table 1).

### Observation-based data and reanalysis products

Climatological data for ocean temperature and salinity from the WOA, as well as monthly EN4.2.2 temperature and salinity datasets (referred to as EN4)^59^ are used for further evaluation. Ocean reanalysis products used for comparison included the Simple Ocean Data Assimilation version 3.4.2 (denoted as SODA3) for the period 1980–2019^60^ Estimating the Circulation and Climate of the Ocean version 4 release 3 (denoted as ECCO4) for the period 1992–2015^61^ from the National Aeronautics and Space Administration, Ocean Reanalysis System 5 (ORAS5) for the period 1958–2022^19^ from the ECWMF, and Global Ocean Physics Reanalysis (GLORYS12) for the period 1993–2016^62^ from the Copernicus Marine Environment Monitoring Service. Additionally, ERA5 atmosphere reanalysis data from 1945 onwards are incorporated. Observational sea surface heat flux datasets from the National Oceanography Centre Southampton (NOCS) period 1973–2014^63^ and SeaFlux Ocean Surface Bundle Climate Data Record (OSB-CDR) period 1988–2021^64^ are also employed for model validation in this study. Detailed information on these reanalysis datasets is provided in Supplementary Table 2.

### Definition of GMOC and associated indices

#### Global meridional overturning circulation

The stream function of GMOC is defined as zonally integrated volume transport, measured in Sv (1 Sv = 10^6^ m^3^/s), from the depth level z to the ocean surface *ŋ* at latitude y^18,28^ It can be expressed as:

3 $$\varPsi ( \, y,z)=\int _{z}^{\eta }\int _{{x}_{W}}^{{x}_{E}}v(x,y,z)dxdz,$$

where, *v* represents the meridional velocity, and *x*_*W*_ and *x*_*E*_ denote the western and eastern boundaries of the global ocean, respectively.

#### GMOC index

Given that the spatial patterns of the principal component (PC) obtained through empirical orthogonal function (EOF) analysis are not consistent across different models, we employ a specific method to define the GMOC index. The GMOC index is defined as the linear regression coefficient of the instantaneous time slice (monthly in this case) GMOC state over the temporal mean distribution of the CDA mean state. The formula is expressed as:

4 $${G}_{{moc}}=\frac{{cov}({G}_{t},\,{G}_{m})}{{{\sigma }_{{G}_{t}}*\sigma }_{{G}_{m}}},$$

where, ${cov}\left({G}_{t}\,,\,{G}_{m}\right)$ represents the covariance between ${G}_{t}$ and ${G}_{m}$. Specifically, it is calculated as ${cov}\left({G}_{t}\,,\,{G}_{m}\right)=\frac{\frac{1}{{J}_{0}}{\sum}_{j}\frac{1}{{K}_{0}}{\sum}_{k}{G}_{{{\rm{t}}}}({{\rm{j}}},{{\rm{k}}})*{G}_{m}(j,{k})*{\sigma }_{{G}_{t}}}{{\sigma }_{{G}_{m}}}$ (where j and k denote the indices for the y‒z plane, and the sums are taken over the appropriate ranges of j and k). Here, ${G}_{m}$ is the mean state of CDA GMOC, ${G}_{t}$ is the instantaneous time slice of GMOC, ${J}_{0}$ and ${K}_{0}$ are numbers of points on the y‒z plane, and ${\sigma }_{{G}_{t}}$ and ${\sigma }_{{G}_{m}}$ are the standard deviations of ${G}_{t}$ and ${G}_{m}$, respectively.

#### North Atlantic deep water

The NADW index is defined as the average thickness between two isopycnals of σ_1.5_ over the domain of 55°–35° W and 45°–65° N^65,66^ Here, *σ*_*1.5*_ represents the potential density referenced to a depth of 1500 m. In this study, we chose *σ*_*1.5*_ = (34.56, 34.68) as the two isopycnals for CESM-HIS, CESM-CDA, CM2-CDA, ORAS5, and EN4, and *σ*_*1.5*_ = (34.52, 34.56) for CM2-HIS. The differences in the isopycnals chosen for different simulations are attributed to the distinct characteristics of deep mode water formation and evolution in each model.

#### Residual circulation of the Antarctic Circumpolar Current

The ACC-RC denotes the net transport of Ekman effects, while being compensated by eddy-induced transport^39^ It reflects the overall balance between the direct impact of Ekman effects and the counteracting influence of eddy-induced transport. We calculate the ACC-RC from the following formula^67^

5 $${\varPsi }^{*}={\varPsi }_{{Ekman}}+{\varPsi }_{{Eddy}}=-\frac{{\tau }_{x}}{{\rho }_{0} \, f}+{K}_{e}\frac{\partial \rho }{\partial z}/\left(\frac{\partial \rho }{\partial y}\right),$$

where, *τ*_*x*_ represent wind stress, *f* is the Coriolis parameter, *K*_*e*_ is eddy diffusivity coefficient, which is set to 1000 m^2^/s in this study^68^ and $\frac{\partial \rho }{\partial z}/(\frac{\partial \rho }{\partial y})$ is the slope of isopycnal. Similar to the GMOC index, we define the ACC-RC index as the linear regression coefficient of the instantaneous time-slice (monthly in this particular case) distribution of the ACC-RC system in relation to its temporal mean distribution.

#### Tropical diffusive mixing

We evaluate the kinetic energy dissipation rate for the analysis of the tropical diffusive mixing effects, which are crucial for maintaining global circulation. Because the turbulence is not resolved in our experiments, only the diagnosed kinetic energy dissipation rate of turbulence is computed. The following equation represents the diapycnal advection-diffusion balance, which is fundamental in our analysis:

6 $$\frac{D\rho }{Dt}=\frac{\partial (\Gamma \varepsilon {N}^{-2}\frac{\partial \rho }{\partial z})}{\partial z},$$

where, ${N}^{-2}=-\frac{g}{{\rho }_{0}}\frac{\partial \rho }{\partial z}$, *ρ* represents the potential density, *ρ*_*0*_ is the reference density, and *ε* is the diagnosed kinetic energy dissipation rate. The buoyancy flux is related to the turbulence *ε* through the constant efficiency factor *Г*^69^ The expression for *ε* on each layer, which is derived from relevant physical principles, is as follows:

7 $$\varepsilon=\int _{z}^{0}-\frac{g}{\Gamma {\rho }_{0}}\frac{d\rho }{dt}dz+C(x,y),$$

In this study, we set *Г* = 0.2 and *ρ*_*0*_ = 1026 kg/m^3^. The constant *C* is ascertained by ensuring that the minimum value of the integration equals 0. The vertical mean of ε is defined as the mean energy dissipation rate *ε*_*m*_ in 30° S–30° N. The tropical energy dissipation index *ε*_*m*_*(t)* is defined as the linear regression coefficient of the monthly time slice of *ε*_*m*_ with respect to its temporal mean distribution.

### Surface buoyancy flux

Following Cerovečki et al.^70^ we calculate the surface buoyancy flux, B, using equation:

8 $$B=\frac{g}{{\rho }_{0}}\left(\frac{\alpha {Q}_{H}}{{c}_{p}}+{\beta S}_{0}{Q}_{F}\right),$$

where g is the acceleration due to gravity, ρ_0_ is the reference density of seawater and S_0_ is the sea surface salinity. The net surface heat (Q_H_; W m^−2^) and freshwater (Q_F_; kg m^−2^ s^−1^) fluxes are positive for surface ocean inputs of heat and freshwater, respectively. α and β are the thermal expansion coefficient and saline contraction coefficient, respectively, and we account for their variations with latitude and time.

### Diapycnal mixing velocity

Following Ferrari et al.^71^ and Cimoli et al.^72^ the diapycnal velocity—describing the movement of water mass across density surfaces—is calculated as follows:

9 $${w}^{*}=\frac{{\partial }_{z}M}{{\partial }_{z}b},$$

where ∂_z_M denotes the vertical gradient of the buoyancy flux (derived as M = Γε, with Γ = 0.2 the turbulent flux coefficient and ε the turbulent kinetic energy dissipation rate), and ∂_z_b represents the vertical gradient of buoyancy (equivalent to the squared buoyancy frequency N^2^, a measure of ocean stratification stability).

### Correlation statistics significance

Correlation significance is assessed via *t*-test, with critical values (r_α_) calculated as: ${r}_{\alpha }=\frac{t}{\sqrt{{t}^{2}+{df}}}$. For a 960-month sample (α = 0.001), coefficients *r* > 0.105 indicate 99.9% significance, while *r* > 0.089 denotes 99% significance. For ORAS5 comparisons (780 monthly data points, 1958–2022), the critical *r*_*α*_ is 0.117.

## Supplementary information

Supplementary Information

Peer Review file

## Data Availability

Data related to the manuscript can be downloaded from the following: ERA5, https://cds.climate.copernicus.eu/datasets; HadISST, https://www.metoffice.gov.uk/hadobs/hadisst/; OISST, https://www.ncdc.noaa.gov/oisst/data-access; Argo, ftp://ftp.ifremer.fr/ifremer/argo/geo/; WOA, http://www.ncei.noaa.gov/; EN4, https://www.metoffice.gov.uk/hadobs/en4/; SODA3, https://www2.atmos.umd.edu/; ECCO4, https://ecco-group.org/; ORAS5, https://cds.climate.copernicus.eu/; GLORYS12, https://data.marine.copernicus.eu/product/GLOBAL_MULTIYEAR_PHY_001_030/services; RAPID, https://rapid.ac.uk/; MOVE, https://usclivar.org/amoc/amoc-time-series; NOAC, 10.1594/PANGAEA.959558; NOCS, https://gdex.ucar.edu/datasets/d260003/; OSB-CDR, https://www.ncei.noaa.gov/data/ocean-heat-fluxes/access/. Experimental data are available via Zenodo at 10.5281/zenodo.20627731^73^ or from the corresponding author on request.

## Code availability

All original figures are generated using MATLAB R2025a (https://www.mathworks.com). The code for climate model data processing and analyses is available via Zenodo at 10.5281/zenodo.15070241^73^.
