# Supplementary material for: The coupled reanalysis global meridional overturning circulation imprinted by historic volcanic eruptions
Source: Nat Commun. 2026 Jul 16;17:8703. doi: 10.1038/s41467-026-75651-z (PMC13490444; doi:10.1038/s41467-026-75651-z)
Supplement: Supplementary file 1 [file 41467_2026_75651_MOESM1_ESM.pdf]

Supplementary information for

The Coupled Reanalysis Global Meridional Overturning Circulation Imprinted by Historic Volcanic Eruptions

by

Yingjing Jiang[1], Shaoqing Zhang[2*], Yang Gao[3*], Lixin Wu[2,4*], Lv Lu[5], Zikuan Lin[2], Wenju Cai[2, 4], Deliang Chen[6,7], L. Ruby Leung[8], Bin Wang[9], Xueshun Shen[10], Mingkui Li[2], Xiaolin Yu[2], and Xiaopei Lin[2]

[1]Institute of Marine Sciences, College of Science, Shantou University, Shantou, China

[2]Key Laboratory of Physical Oceanography, Ministry of Education/Frontiers Science Center for Deep Ocean Multispheres and Earth System/College of Oceanic and Atmospheric Sciences, Ocean University of China, Qingdao, China

[3]Frontiers Science Center for Deep Ocean Multispheres and Earth System/Key Laboratory of Marine Environmental Science and Ecology, Ministry of Education, Ocean University of China, Qingdao, China

[4]Laoshan Laboratory, Qingdao, China

[5]College of Intelligent Systems Science and Engineering, Harbin Engineering University, Harbin, China

[6]Department of Earth System Sciences, Tsinghua University, Beijing, China

[7]Department of Earth Sciences, University of Gothenburg, Gothenburg, Sweden

[8]Atmospheric, Climate, & Earth Sciences Division, Pacific Northwest National Laboratory, Richland, WA, USA

[9]Department of Atmospheric Sciences and International Pacific Research Center, University of Hawaii at Manoa, Honolulu, HI, USA

[10]The Center of Earth System Modeling and Prediction, CMA, Beijing, China

Contents of this file

Supplementary Text 1–4

Supplementary References

Supplementary Figs. 1–30

Supplementary Tables 1–3

Supplementary Text 1–4:

Supplementary Text 1: More evaluations of atmospheric and oceanic states in two coupled reanalyses

a, Convergent AMMC (atmospheric mean meridional circulation) mean states

The AMMC is denoted as the temporal mean atmospheric meridional stream function, which can be expressed following Sun & Zhou [1] and Jiang et al. [2] as:

$$\Psi = \frac{2\pi a cos\emptyset}{g}\int_0^p \bar{v}dp\,, \qquad (Supplementary\,Eq.1)$$

Here, $\bar{v}$ is the zonal average meridional wind, p is the pressure, $\emptyset$ is the latitude, g is the gravitational acceleration, and a is the radius of the Earth.

In a manner similar to the definition of the GMOC index (see **Methods**), we define the AMMC index as the linear regression coefficient of the monthly AMMC with respect to its temporal mean value obtained from ERA5.

Supplementary Fig. 4 shows the root-mean-square error (RMSE) distributions of AMMC for model simulations and CDA estimates against ERA5. The large errors of model-simulated AMMC are distributed over the tropics and areas lying at 60°–80° S in the Southern Hemisphere (SH). The tropical error of CM2-HIS is larger than that of CESM-HIS, which can be attributed to the coarser resolution of the atmospheric model in CM2. The substantial errors in these regions could potentially affect the accuracy of subsequent analyses related to the AMMC and its interaction with other climate components. The observational constraint greatly reduces the error of the AMMC in both CDA experiments. The improvement in CM2-CDA is larger than that in CESM-CDA. This indicates that observational data play a crucial role in enhancing the accuracy of AMMC estimates, and the greater improvement in CM2-CDA suggests that it may benefit more from the incorporation of observational information as compared with CESM-CDA. The AMMC obtained via CDA is generally consistent with ERA5 (**Supplementary Figs. 4e–f**), although it is a little weaker at high latitudes.

b, Convergent ocean temperature and salinity estimates

By analyzing the timeseries of ocean temperature and salinity at different depths (0–500, 500–2000, and 2000–5000 m) over the latitude range of 60° S–60° N (as shown in **Supplementary Fig. 1**), we note that, although systematic differences between CDA estimates (CESM-CDA and CM2-CDA) and the objective analysis product EN4 exist, they are much smaller when compared with the differences between model simulations (CESM-HIS and CM2-HIS) and the EN4 product. Moreover, the phases in the variations of CDA-

estimated ocean temperature and salinity are highly consistent with EN4. In addition, consistent with previous studies [3,4], the timeseries of ocean temperature and salinity (**Supplementary Fig. 1**) show that the direct oceanic observation-constraints blending to the coupled dynamics take a few years for the ocean equilibrium in which the upper ocean is more quickly than the deep ocean (compare lower panels to upper panels) while salinity is slower than temperature (compare right panels to left panels). The timeseries of atmospheric work exerted on the oceans (W_{A2O}) (**Supplementary Fig. 2e**) and oceanic heat flux to the atmosphere (HF_{O2A}) (**Supplementary Fig. 3e**) show that the spinup of surface fluxes is very quick in the CDA process, taking a couple of months to reach their equilibrium. It's worth to mention that the climatological restoration for deep ocean in this study as a part of multiscale oceanic data-constraints is crucial for coherence quick spinup of upper and deep oceans to rebuild the ocean's stratification, which will be analyzed and discussed more in **Supplementary Text 2c** as the ocean's stratification of CDA-produced coupled reanalyses is analyzed in details.

We also present the results of other CDA experiments, namely CESM-CDA0 and CM2-CDA0, which were initialized with different initial conditions on January 01, 1978. These experiments have been evaluated in Jiang et al. [2]. Both CDA systems converge from different initial conditions that are produced from different coupled spin-up states (CESM-CDA0 starts from the historical simulation on 01-01-1869 and CM2-CDA0 starts from the previous assimilation on 01-01-1978 from Chang et al. [5]. This convergence indicates that the CDA method possesses a certain robustness in handling different initial setups, which is crucial for obtaining reliable and consistent results in the context of oceanographic studies.

Although coherent convergence of upper- and deep-ocean temperature and salinity in CDAs to the EN4 observation product occurs, the CDA results are somewhat sensitive to the change in oceanic observing system as the Argo profiles are assimilated (see the period after 2000 in **Supplementary Figs. 1c–d**). This sensitivity implies that further investigations are needed to understand how the CDA performance might be affected by different configurations of the oceanic observing system, and to develop strategies to mitigate potential uncertainties introduced by such changes.

c, Convergent atmospheric work exerted on the ocean and oceanic heat fluxes to the atmosphere

To gain an understanding of the balance between the atmosphere and ocean achieved through CDA, we conduct an examination of air–sea interaction processes within the CDA framework. To quantitatively assess the atmospheric work exerted on the ocean (W_{A2O}), we utilize the following formula based on Huang et al. [6]:

$$W_{A2O} = \tau_x * u^O + \tau_y * v^O, \qquad (Supplementary\ Eq. 2)$$

where, (τ_x, τ_y) are the wind-stress at the sea surface and (u^o, v^o) are the sea surface currents (for verification with "observation", we use geostrophic currents in this case).

Supplementary Fig. 2 presents the error distributions of W_{A2O} for both model simulations and CDA results in comparison with the observed product, as well as the timeseries of the global mean W_{A2O}. The observed sea surface wind-stress is obtained from ERA5, while the geostrophic currents are derived from the Archiving, Validation and Interpretation of Satellite Oceanographic data (AVISO). **Supplementary Fig. 2a–b** shows that the errors of model simulations are substantial in low-latitude areas of the North Pacific, areas near 20° S in the Indian Ocean, and ACC areas of the Southern Ocean. The substantial errors in these regions may potentially lead to inaccuracies in subsequent analyses related to air–sea interaction processes and climate model predictions. In addition, the errors are also substantial in the areas of the Kuroshio, Gulf Stream, and near 10° S in the Pacific.

The much smaller errors of W_{A2O} obtained via CDA compared with the corresponding model simulation not only indicate its superiority in approximating the actual situation but also suggest that it can provide more reliable estimates for further studies on air–sea interaction dynamics. From the timeseries of the normalized W_{A2O}, we observe that the error of the CESM is approximately 1 mW/m^2 larger than that of the CM2. We also note that, in addition to reducing the W_{A2O} bias, CDA substantially corrects the phases of W_{A2O} variability. This correction is of great importance because it can enhance the accuracy of predicting air–sea interaction phenomena based on W_{A2O}.

To analyze the oceanic heat flux to the atmosphere, we examine it through the surface turbulent heat flux (HF_{O2A}), which consists of sensible (Q_S) and latent (Q_L) heat fluxes as described by Cronin et al. [7]. The relationship can be expressed as:

$$HF_{O2A} = Q_S + Q_L, \qquad (Supplementary\ Eq.\ 3)$$

As shown in **Supplementary Fig. 3**, coupled data assimilation (CDA) effectively reduces the climatological biases of upward ocean-to-atmosphere heat flux (HF_{O2A}), particularly over regions with vigorous air–sea interaction including the Gulf Stream, Kuroshio and eastern equatorial Pacific. The model simulations (CESM-HIS and CM2-HIS) exhibit prominent positive (and negative) heat-flux overestimation across these key domains [8], while such systematic biases are largely mitigated after CDA implementation for both model configurations. The substantial reduction of heat flux errors by CDA compared with model simulation, especially in high latitudes of the North Atlantic and ACC areas, indicates that CDA can effectively correct inaccuracies in estimating the oceanic heat flux to the atmosphere. This improvement is of great importance

because it enhances the reliability of climate models in simulating the heat exchange process between the oceans and atmosphere.

In terms of temporal variability (**Supplementary Fig. 3e–f**), HF_{O2A} anomalies from CESM-CDA and CM2-CDA evolve coherently throughout the study period and match to observational datasets closely, including NOCS (National Oceanography Centre Southampton surface turbulent heat flux dataset), OSB-CDR (SeaFlux Ocean Surface Bundle Climate Data Record) as well as ERA5 reanalysis. For instance, the CDA-corrected HF_{O2A} anomalies reproduce the observed temporal evolution — their fluctuation phases during 1997–2005 over the Gulf Stream and 1995–2010 over the Kuroshio align well with reference datasets in both peak and trough timings. It is worth to mention that ERA5-derived turbulent heat fluxes have features with region-dependent systematic biases and decadal trend uncertainties. These features may originate from temporally-discontinuous moisture/temperature analysis increments induced by the change of observational systems, evident as in-situ buoy measurements over tropical Indian Ocean and mid-latitude western boundary current regions [9,10]. Similarly, the observation-based products (NOCS and OSB-CDR) also have their own uncertainties—for example, over the Kuroshio region during 2000–2007, NOCS and OSB-CDR exhibit large discrepancies, while both CDA simulations and ERA5 fall between them, which serves only as additional references here (**Supplementary Fig. 3f**).

***d*, Rapid spin-up of the GMOC in the CDA experiments**

The time series of GMOC (below 2000m) root-mean-square differences (RMSD) between the CDA experiments and their corresponding historical (HIS) simulations demonstrate a remarkably rapid spin-up behavior across the global and regional ocean basins (**Supplementary Fig. 5**). In all domains, the GMOC RMSD stabilizes within 1–2 years after the start of assimilation, as evidenced by the leveling off of both the monthly time series and their low-frequency running means (i.e., 6-month and 24-month).

This efficient convergence arises from two key features of the experimental design. First, the CDA experiments are initialized from the balanced state of the historical simulations, so the system starts from an existing quasi-equilibrium rather than from a climatology – mitigating the need for a long adjustment to reach a basic equilibrium. Second, the combination of atmosphere-ocean observation constraints and deep-ocean climatological restoring improves the ocean stratification, allowing the system to quickly transition from the initial equilibrium to a new observation-constrained balanced circulation [4,14].

Supplementary Text 2: More evaluations of GMOC mean state and variability in two coupled reanalyses

***a*, Similar distribution pattern of GMOC mean states**

We first calculate the mean states of GMOC in two historical model simulations CESM-HIS and CM2-HIS, two CDA estimates CESM-CDA and CM2-CDA, as well as four ocean reanalysis products SODA3, ORAS5, ECCO4 and GLORYS12 (**Supplementary Fig. 6**). Although differences exist in detailed features, the mean states of GMOC in the two free model simulations of CESM-HIS and CM2-HIS exhibit a generally-similar pattern that has a substantial northward transport circulation evident above 3000 m, underlain by a weak southward return flow (panels **a** & **b**). We may comprehend such a similarity by their overall common approximations on physics associated with topography and subgrid processes as well as perfect balance and conservation in model regime. While substantial differences in a large scope between the coupled reanalysis and other reanalysis products are observed (compare panels **c** & **d** to panels **e–h**), the two coupled reanalyses show however an overall convergent feature. Compared to model simulations, the coupled reanalyses adjust the upper northward transport circulation to three separate centers within the 10 Sv contour circle. The Antarctic Circumpolar Current (ACC)-associated transport becomes weaker, while the North Atlantic Deep Water (NADW)-associated transport largely strengthens. Both CDA estimates reveal a new circulation center between 10° and 30° S at a depth of 500–2000 m, while this center appears very weak in SODA3 and ECCO4, but absent in ORAS5. This feature is also present in GLORYS12, with a comparable intensity but a relatively northward-shifted position.

For the deep circulation system of GMOC, all data-constrained results are significantly stronger than the free model simulation. The maximum strength of NADW-associated transport in CDA estimates is approximately 20% stronger than their free simulation counterparts, extending vertically by 1000 m to reach a depth of 4000 m. In addition, compared to free model simulations the CDA-estimated GMOCs have a much intensified deep southward return circulation below 2500 m, exhibiting double centers between 3000 and 4000 m, one at 30° S and the other at the equator. In other four ocean reanalysis products, the pattern of GMOC mean state resembles a blend of model simulation and CDA estimate: although the upper northward transport circulation exhibits two centers marked by a stronger ACC and weaker NADW within a continuous 10 Sv contour circle, these reanalysis products exhibit a much stronger and wider scope deep southward return circulation than the model simulations and CDA estimates do.

Basin decomposition analysis of meridional overturning reveals that the new CDA GMOC center between

10° and 30° S at a depth of 500–2000 m mainly arises from the CDA-enhanced Atlantic subtropical gyre system, resulting in a markedly different Atlantic meridional overturning circulation (AMOC) (**Supplementary Fig. 7**). In contrast, the substantial southward transport in the CDA estimates largely originates from an enhanced Indo-Pacific deep ocean tropical system, corresponding to a strong Indo-Pacific meridional overturning circulation (IPMOC) (**Supplementary Fig. 8**).

In general, verification of ocean currents is difficult due to the lack of direct observations. Here we use the "observation" data on ocean currents derived from Argo-drift information of 950–1150 m [11,12] to validate ocean currents (**Supplementary Fig. 9**). As pointed out by Su et al. [11], there are widespread global discrepancies between modeled and observed mid-depth ocean currents. From **Supplementary Fig. 9**, we observe that in general the free model simulations underestimate current velocities globally, and the underestimation is especially pronounced over the tropics and mid-latitudes. Such an underestimation pattern may explain the simple GMOC pattern in the free model simulation shown in **Supplementary Figs. 7a–b**. The ocean current velocity distributions of SODA3 and ORAS5 are similar to that of CDA estimates, while ECCO4 is close to the model simulations. From the perspective of average velocity, ECCO4 is too small, while CESM-CDA and CM2-CDA are the closest to the "observation" compared to other ocean reanalysis products. From the distribution of RMSE, CEMS-CDA and CM2-CDA are significantly smaller than SODA3 and ORAS5, especially in the ACC region of the southern hemisphere and between 35° N and 75° N in the northern hemisphere. This suggests that the GMOC's center between 10° and 30° S at a depth range of 500–2000 m in both coupled reanalyses are very likely to be a robust estimate.

In deep oceans, current velocities in free model simulations are generally small (**Supplementary Figs. 10a–b**). Large-value regions are predominantly concentrated along the western boundary of the Atlantic and in the Antarctic Circumpolar Current (ACC) areas, with a maximum velocity reaching 2 cm/s. In contrast, in other regions, the velocities of ocean currents are generally less than 0.5 cm/s. It's worth to note that in open deep-ocean regions, such as the Pacific and central South Atlantic, the current velocities of the CESM are larger than those of the CM2, and the CESM provides greater detail. This may be attributed to the fact that the CESM has a slightly higher horizontal and vertical resolution compared with the CM2.

The deep-ocean currents estimated by CDA are substantially enhanced globally compared with the model simulations (**Supplementary Figs. 10c & 9d**), although the increases in the ACC and WBC areas are relatively small. This accounts for the enhancement of the deep-circulation system of GMOC in CDA estimates. Other reanalysis products also have deep-ocean currents enhanced in different degree (**Supplementary Figs. 10e–h**)

compared to the free model simulation, which are consistent with intensified deep circulation of GMOC (**Supplementary Figs. 6e–h**). However, owing to the lack of observations in deep portions of the oceans below 2000 m, it's difficult to determine whether this enhancement aligns with the real-world situation. Considering ocean stratification and the improvement of 950-1150 m currents, we may comprehend that the enhanced deep-ocean currents in data-constrained products especially in CDA that takes atmosphere–ocean coupling into account closer to the real world than free model simulations.

The convergence of two coupled reanalyses in representing the GMOC including its mean state and variability is a remarkable finding, which is different from the phenomenon that the two historical simulations CESM-HIS and CM2-HIS show a similar GMOC mean state. In practice, model simulations share common structural and parametric limitations, including coarse discretization of fluid dynamics, overly smoothed topographic representations, and parameterizations that approximate subgrid physical processes. Combined with biased model balance and conservation, these common deficits tend to imprint similar but distorted features on the simulated mean state of GMOC. When these models are constrained by observations in reanalysis, the resulting reanalyzed GMOCs are shaped not only by observational constraints but also by these underlying model biases. As a result, it easily happens that the reanalyzed GMOCs tend to have different features due to the adverse impacts of differences in model biases and assimilation algorithms. Here the convergence of the GMOC mean state and variability in two coupled reanalyses suggests that our CDA data constraint approach with atmosphere-ocean coupling and multiscale data-blending mechanisms does help reduce imbalances that are introduced during data-model integration.

***b*, The AMOC and IPMOC components of GMOC**

The AMOC, being an important component of GMOC, plays a crucial role in both local and global climates [13]. In **Supplementary Fig. 7a–d**, the mean states of the AMOC for model simulations and CDA estimates are presented. Meanwhile, in **Supplementary Fig. 7e–h**, the corresponding versions of ocean reanalysis products are presented. The mean states of the AMOC in CESM-HIS and CM2-HIS exhibit a similar characteristic, featuring northward flows in the upper 1000 m and southward flows in the 1500–3000 m depth range. The intensity of the upper circulation of the AMOC in CESM-HIS is marginally stronger than that in CM2-HIS. In contrast, the strength of the deep southward transport circulation in CM2-HIS is greater than that in CESM-HIS. The CDA process substantially enhances the AMOC, and it also generates a new circulation core at a depth of 1000 m and latitude of 20° S. Consequently, in both the CESM-CDA and CM2-CDA, the AMOC displays a "double-core" northward transport circulation structure, which can be attributed to an

enhanced South Atlantic subtropical gyre.

From **Supplementary Fig. 7e–g**, we observe that the features of the AMOC in ocean reanalysis products appear to be intermediate between those of the model simulations (**Supplementary Fig. 7a&b**) and those of the CDAs (**Supplementary Fig. 7c&d**). Compared with the model simulations, the AMOCs in ocean reanalysis products exhibit strengthened circulations in the upper 3000 m of the South Atlantic and relatively small southward transport circulations in deep tropical oceans. However, unlike in the CDAs, the strengthened South Atlantic circulations do not form an enclosed circulation core. Meanwhile, the circulation cores associated with the NADW in ocean reanalysis products appear to be similar with those of the model simulations, with the latter cores remaining above 3000 m. In contrast, the corresponding CDA counterparts extend below 4000 m.

Besides presenting the IPMOCs for model simulations and CDA estimates, we also display the IPMOCs of ocean reanalysis products in **Supplementary Fig. 8**. Similar to the IPMOCs of the CDA estimates, the IPMOCs in ocean reanalysis products exhibit negative (southward transport) circulations, particularly in ECCO4 and ORAS5, which are predominant over the area south of 40° N. This supports the hypothesis that the enhanced deep southward transport circulations in CDA-estimated GMOCs (shown in **Supplementary Figs. 6c–d**) primarily come from the data-constrained Indo-Pacific Oceans, and therefore, they are most likely close to the real world.

c, Coherent ocean stratification in coupled reanalyses

Previous studies have suggested that balanced and coherent states of the atmosphere and oceans are crucial for reconstructing the three-dimensional flows and overturning circulations of the oceans through data assimilation [14,2]. To help understand the estimates of oceanic currents and overturning circulation in coupled reanalyses, following the evaluation of the AMMC, W_{A2O}, and HF_{O2A} in **Supplementary Text 1**, here we further investigate ocean stratification of temperature, salinity, and potential density in CDA to confirm the coherence of ocean stratification within the procedure of our CDA model-data integration.

We compare the zonal-mean deep-ocean (2–6 km) potential density and the global temperature, salinity, and density biases (**Supplementary Fig. 11)**. The results reveal substantial improvements in the representation of deep-water mass structure in the CDA experiments relative to their corresponding historical free model simulations. In the density cross-sections (**Supplementary Figs. 11a–f**), the CESM-CDA and CM2-CDA experiments reproduce the observed meridional distribution of key isopycnals (e.g., 34.76 and 34.84 kg m^{-3}) far more accurately than CESM-HIS and CM2-HIS, closely matching to the structures in the ORAS5 and

WOA09 observational climatology.

These structural improvements are corroborated by the vertical profiles of global-mean deep-ocean biases (**Supplementary Figs. 11g–i**). Specifically, CM2-HIS exhibits large cold biases in potential temperature, while CESM-HIS shows prominent positive biases in salinity, leading to significant positive biases in potential density in both free-running simulations. In contrast, both CESM-CDA and CM2-CDA substantially reduce these temperature, salinity, and density biases, bringing them much closely aligned with observational and reanalysis references. Notably, the CDA experiments also outperform to several other widely used ocean reanalysis products (e.g., ORAS5, SODA3, ECCO4) in key aspects, particularly minimizing deep-ocean density biases, which highlights the effectiveness of the CDA framework in constraining deep circulation and water mass properties.

We compute the RMSE reduction rates of potential density within the CDA framework relative to the ocean reanalysis results (SODA3, ECCO4, and ORAS5), with the latter being verified against the WOA (as depicted in **Supplementary Fig. 12**). In comparison with the results of ocean reanalysis, the RMSE of potential density in most areas of the two CDAs significantly declines, particularly below 2000 m, with a reduction rate ranging from 60% to 80%. These results indicate that in areas where observational data are scarce in the deep oceans, employing stronger restoration schemes can mitigate deep-ocean model biases and lead to balanced and coherent ocean stratification. In the upper middle ocean (0–2000 m), the CDA-estimated potential density, employing multiscale filtering assimilation, is also improved in many areas, especially when compared with ECCO4 and ORAS5.

_d_, Observation-convergent geostrophic component of GMOC

We calculate the geostrophic GMOC using observed and CDA-derived ocean temperature and salinity data, and subsequently verified its geostrophic component. The following formula for the geostrophic meridional velocity (v_g) is derived based on the geostrophic balance and thermal wind relationship, which are fundamental in oceanographic studies [15]:

$$\boldsymbol{v_g}(k) = \boldsymbol{v_g}(k+1) - \frac{g}{f}\frac{[z(k+1) - z(k)]}{\sigma(k)}\frac{\partial \sigma(k)}{\partial x}, \qquad (Supplementary\ Eq. 4)$$

where, g is the gravitational acceleration, f is the Coriolis parameter, k is the *k-th* vertical grid with k increasing downwards, z is the grid depth, and $\sigma(k)$ is the potential density referenced to $z(k)$. The geostrophic velocity is reconstructed with the level of no motion at the ocean bottom. Referring to previous research [16], a small spatially uniform velocity *O (0.1 mm/s)* is removed afterwards to ensure zero meridional transport.

The geostrophic GMOC displays a general characteristic of positive (northward-transport) circulations in the SH and negative (southward-transport) circulations in the NH, as shown in **Supplementary Fig. 13**. However, in the SH, although the geostrophic GMOCs in CDA estimates, ocean reanalysis products, and the "observation" are characterized by a large positive circulation with a small negative center in the upper 500 m of the subtropics, the positive circulation in model simulations is much smaller and weaker, and vice versa for the negative circulation. In the NH, although the CDA-estimated geostrophic GMOCs exhibit negative, positive, and negative patterns in the upper 1000 m at low, middle, and high latitudes, respectively, similar to those of ocean reanalysis products and the "observation", the patterns in model simulations are particularly weak, especially in the tropics within CESM-HIS.

The above analyses are substantiated by the distributions of differences between the two model GMOCs, those between the two CDA-estimated GMOCs, and those among the ocean reanalysis products, as depicted in **Supplementary Fig. 13**. The strong geostrophic transport in the SH may be attributed to the more open nature of the oceans in the SH, while the weaker geostrophic transport in the NH may be attributed to the more complex ocean basin structure in the NH. Owing to common discretization features, such as an approximately 100 km horizontal resolution, free model simulations tend to produce generally similar GMOC patterns. However, these patterns deviate substantially from the real world owing to the approximation of physics and omission of certain scales in the models. Our CDA results with independent models suggest that multiscale data–model incorporation, consisting of deep-ocean bias treatment, can recover the GMOC mean state by a credible degree.

The evaluation of geostrophic GMOC metrics demonstrates that both CESM-CDA and CM2-CDA outperform to their historical simulations (HIS) across the full-depth and deep-ocean (below 2000 m). In terms of GMOC representation accuracy (**Supplementary Figs. 14a–b**), the CDA experiments show substantial reductions in RMSE of geostrophic GMOC and significant improvements in pattern correlation coefficient (PCC) relative to HIS. Specifically, compared to the model simulations, CESM-CDA reduces full-depth geostrophic GMOC RMSE by 18.3% and deep-ocean RMSE by 28.7%, while CM2-CDA reduces full-depth RMSE by 19.1% and deep-ocean RMSE by 35.2%. Concurrently, the PCC increase notably in both basins, with improvements of up to 24% in the deep-depth domain for CM2-CDA. These results confirm that CDA effectively constrains both the large-scale structure and deep-ocean components of the GMOC.

Consistent with the basin-scale GMOC improvements, the representation of AMOC transport at 26°N is also enhanced in the CDA experiments (**Supplementary Figs. 14c–d**). Compared to the historical simulations, the CDA

yields mean deep AMOC transports (averaged below 2 km) that are much closer to the Rapid observation and reanalysis references (e.g., SODA3, ECCO4). Although the mean deep AMOC transport of CESM-HIS appears close to the Rapid observation, its vertical distribution is physically unrealistic (**Supplementary Fig. 14c**). By contrast, the deep AMOC biases in both HIS simulations are effectively corrected in the CDA experiments, yielding mean transports and vertical structures far more consistent with observation. These results confirm that CDA significantly improves the representation of GMOC, including the deep ocean.

***e*, Convergent GMOC variability in two coupled reanalyses**

We show the STD values of the GMOCs for model simulations and various assimilation products in **Supplementary Fig. 15**. In general, large STD values of the GMOC are predominantly located in the ACC (Antarctic Circumpolar Current residual circulation), tropical, and NADW areas. However, these values are relatively lower in model simulations but much higher in ocean reanalysis products, particularly in the ACC-RC region and tropics of SODA3 and ECCO4. Although the distribution patterns of GMOC STD values for the two CDAs and ORAS5 exhibit a generally similar characteristic, featuring high values in the tropics, deep penetration in the ACC-RC area, and relatively high values at high-latitudes of the NH, the patterns of SODA3 and ECCO4 deviate from this characteristic. Among the seven GMOC STD distributions, which include two model simulations, two CDA estimates, and three ocean reanalysis products, the two CDA estimates exhibit the same distribution centers. However, the values in CM2-CDA are larger than those in CESM-CDA (as can be seen by comparing in **Supplementary Figs. 15c–d**). Consistent with GMOC's behavior, the AMOC shows smaller STD values in the free model simulations than in reanalyses (**Supplementary Fig. 16**). Both CEMS-HIS and CM2-HIS exhibit a similar structure, in which, the maximum values are located in the North Atlantic at approximately 40° N and a depth of 1000–2000 m (panels **a–b**). In contrast, the maximum STD values of CESM-CDA and CM2-CDA are located in tropical oceans at the depth of 500–1500 m (panels **c–d**), while other ocean reanalysis products show a relatively-divergent distribution of AMOC's STD values (panels **e–g**).

To assess GMOC variability, we utilize Atlantic meridional overturning circulation (AMOC) volume transport observations from the RAPID-MOCHA array (Feb. 2004–Dec. 2021) at 26.5° N in the Atlantic [17], along with volume transport data from the Meridional Overturning Variability Experiment (MOVE) mooring observations (Jan. 2000–Jan. 2018) at 16° N in the western Atlantic and the North Atlantic Changes (NOAC) array (Jan. 1993–Dec. 2018) at 47° N [18].

To show timeseries of GMOC variations, we calculate the linear regression coefficient between GMOC states at each time slice and their mean state as a GMOC index, measuring the departure of an instant from the

mean (see **Methods**). The timeseries of GMOC indices for CESM-HIS, CM2-HIS, CESM-CDA, CM2-CDA and the mean of three reference ocean reanalysis products are displayed in **Supplementary Fig. 17a**. Model-simulated GMOC timeseries show little correlation (0.04), whereas the CDA-estimated GMOC timeseries are highly correlated (0.59, far above the 99% significance for 960 degrees of freedom, see **Methods**). We first check the volume transports at 47°, 26°, and 16° N in the Atlantic (where long-term observations exist), as shown in **Supplementary Figs. 17b–d**. The CDA- and reanalysis-estimated transports align well with observations in all three cross-sections, substantially outperforming model simulations and exhibiting correlation above the 99% significance level. All data-constrained estimates fit best at the 26° N RAPID cross-section (compare **Supplementary Fig. 17c** with **Supplementary Figs. 17b-d**), likely owing to the dominance of large-scale circulations in the mid-latitude Atlantic, which facilitates data constraints. For cross-sections at different latitudes, CDA and ocean reanalyses perform differently. In mid-to-high latitudes, both CDA estimates and ocean reanalyses show comparable quality. However, at low latitudes, CDA substantially outperforms ocean reanalysis products. This suggests that in the tropics, where air-sea interactions are highly active, the coupled data assimilation improves the efficiency of model–data integration, enhancing analysis quality.

Given the lack of direct and systematic observations on ocean currents, we evaluate the CDA-estimated GMOC by verifying its geostrophic component (see **Methods**). We further assess the variability of the CDA-estimated GMOCs by verifying their geostrophic components against "observed" geostrophic timeseries derived from an observational dataset (e.g., EN4) [19] using monthly ocean temperature and salinity data. Given that model simulations, CDA estimates, and ocean reanalysis products may have varying compositions of geostrophic and non-geostrophic components, we examine the behavior of geostrophic GMOCs across different timescales (**Supplementary Figs. 17e–g**). In free model simulations, aside from some correlation in the temporal mean geostrophic distribution with "observations," as expected, no correlation in variability is observed at any scale. Overall, the CDA constraint greatly improves the capability of model's geostrophic fitting, particularly in the low-frequency band. However, for the shorter timescales, geostrophic fitting becomes more difficult (**Supplementary Fig. 17g**). At the decadal scale, CDA estimation shows slightly better skill than ocean reanalysis (0.66 vs. 0.64 for 6–10-yr mean correlation). In contrast, ocean reanalysis performs better at the interannual scale (0.51 vs. 0.41 for 1–5-year mean correlation). These differences likely arise from the distinct approaches in information capture: ocean reanalysis relies on specified "observed" atmospheric fluxes, while CDA estimation operates within a coupled model framework, which has more uncertainties in

atmospheric fluxes but achieves a greater atmosphere-ocean coupling balance. The convergence of GMOC variability can be directly explained by the convergence of meridional heat and salinity transport (**Supplementary Fig. 18**). Within the coupled data assimilation framework, the meridional heat and salinity transport from the two CDA experiments exhibit a clear tendency to converge, indicating a reduction in inter-model uncertainty.

Supplementary Text 3: More analyses on physics of GMOC variability

***a*, Mechanisms of multiscale processes in maintaining GMOC**

The GMOC is the consequence of maintaining an atmosphere–oceans balance. As important components of the Earth system, the ocean and atmosphere interact with each other in multiscale processes (**Supplementary Fig. 19**). Excluding various dissipation mechanisms, the coupled atmosphere and oceans on Earth can be regarded as a semi-enclosed system [20]. In this system, the atmosphere "stirs" the oceans, while the oceans feed back to the atmosphere with updated boundary conditions that have redistributed thermal properties [21]. If the coupled atmosphere–oceans system is reduced to a two-dimensional space in the vertical and meridional directions, the atmosphere and oceans can still be viewed as a pair of zonally-averaged self-constrained counterparts, such as the AMMC [22] and GMOC [23]. The AMMC consists of Hadley, Ferrel, and Polar cells, corresponding to Earth's surface wind systems, including the trade winds and westerlies, among others (**Supplementary Fig. 19a).** Under the joint influences of atmospheric wind stress, Coriolis force, and topography, the oceans ultimately form a complex circulation system (**Supplementary Fig. 19b**). Ocean water upwells in the Southern Ocean under wind-driven Ekman effects, and flows northwards to high-latitudes in the Northern Hemisphere (NH), then sinks owing to evaporation and cooling, forming the NADW that continuously transports southwards as the return flow. There are abundant and complex mixing and dissipation processes in the tropics. The variation mechanism of GMOC can be summarized as a three-term balance model [24] **(Supplementary Fig. 19c)**, i.e., the residual between the difference in northern sinking and southern upwelling, subtracting tropical diffusive mixings, drives the change in pycnocline depth anomaly.

However, owing to imperfect Earth modeling and observational systems [25], a complete picture of the historical AMMC–GMOC mean state and variability has yet to be established. Owing to suboptimal discretization and physical schemes in climate models [26], the three-dimensional motion of ocean water simulated by models is different from that in the real world [27]. Models tend to simulate a pair of AMMC–GMOC structures with different features in their own model spaces [2]. The model GMOC is unable to represent the transport of heat, freshwater, carbon, and nutrients between the hemispheres in the real world.

Moreover, measurements of ocean states are very limited [28], usually focusing on the ocean surface [29], or otherwise only available as in-situ temperature and salinity profiles for the subsurface [30]. In this study, we attempt to discover the degree to which a CDA approach can establish the AMMC–GMOC mean state and variability in a coherent and balanced manner of model–data incorporation.

We show the mean states of NADW (**Figs.1g–l**), ACC-RC (**Supplementary Fig. 20**) and tropical diffusive mixing (**Supplementary Fig. 21**). For NADW, both CDA estimates produce a nearly identical isopycnal structure with the observation while ORAS5 only displays somewhat improvement from free model simulations. For ACC-RC, CDA constraints weaken the northward transport between 65°–45° S and strengthen the southward transport around 45° S at 1000–2000 m depth from free model simulations, bringing the CDA ACC-RC mean state closer to the "observation" (**Supplementary Fig. 20**). Since ocean reanalysis uses "observational" atmospheric fluxes, it is expected that ORAS5 shows the best fit for the mean ACC-RC features. For tropical diffusive mixing (ε_m), while the model simulations appear very weak, especially in CESM, both CDA estimates greatly enhance ε_m in the regions of tropical instability waves, WBCs, and the southern and northern equatorial currents across the Pacific, Atlantic, and Indian basins, bringing the estimates close to the level of high-resolution (25 km) ORAS5 ocean reanalysis (**Supplementary Fig. 21**).

***b*, Decomposition of multiscale GMOC modes**

In **Supplementary Fig. 24**, we present the 2–30-yr band-pass filtered timeseries of the North Atlantic Oscillation (NAO) index, Southern Oscillation Index (SOI), and Southern Annular Mode (SAM) index. The NAO employs a PC-based index, which is the timeseries of the leading EOF of sea level pressure (SLP) anomalies over the Atlantic sector within the range of 20°–80° N and 90° W–40° E, as defined by Hurrell & Deser [31]. The SOI is a timeseries that represents the monthly SLP difference between Tahiti and Darwin in the tropical Pacific, as described by Trenberth & Hoar [32]. The SAM also employs a PC-based index, which is the timeseries of the leading EOF of SLP anomalies over the SH within the range of 40°–65° S, as specified by Marshall [33].

Similar to the cases of the AMMC and GMOC mentioned previously, the variabilities of the NAO, SOI, SAM, NADW, ε_m, and ACC-RC in CESM-HIS and CM2-HIS are discrepant. In contrast, those in CESM-CDA and CM2-CDA converge. The NAO, SOI, and SAM estimated by CDA are in line with the ERA5 "observational" result. Furthermore, the NADWs of the two CDAs converge to the objective analysis product EN4 on decadal and multidecadal scales. However, the variabilities of the model simulations and ORAS5 are discrepant and deviate substantially from those of EN4. Moreover, the ACC-RCs of the CDAs are highly in

line with both EN4 and ORAS5 on the interannual scale, corresponding to the interannual variability of wind stress. It is worth mentioning that the ε_m timeseries of CESM-CDA and CM2-CDA are essentially consistent after 1978, especially with regard to changes on interdecadal and annual scales.

***c*, Physics of improved GMOC in CDA**

The improvement in GMOC representation in the CDA experiments can be physically attributed to enhanced representation of key dynamical processes across ocean basins. In the high-latitude Northern Ocean (**Supplementary Fig. 26a**), where deep GMOC variability is dominated by diapycnal mixing, the CDA experiment shows a correlation coefficient of 0.55 with EN4-derived mixing-induced transport, representing a 63% improvement over the HIS simulation. Similarly, in the Tropical Ocean (**Supplementary Fig. 26b**), where diapycnal mixing drives upwelling, mixing, and dissipation in the meridional circulation, the CDA experiment achieves a correlation of 0.80 with EN4, a 73% improvement relative to HIS. In the Southern Ocean (**Supplementary Fig. 26c**), where wind stress and eddy processes dominate the GMOC, the wind-driven Ekman transport in CDA shows a 0.72 correlation coefficient with ERA5, and a 71% improvement over HIS. Notably, despite only assimilating surface atmospheric pressure data, the CDA significantly improves the representation of wind stress and its associated Ekman transport.

These basin-specific results demonstrate that the improved GMOC in CDA arises from enhanced representation of diapycnal mixing in the Northern and Tropical Oceans, and improved wind-driven processes in the Southern Ocean. Due to the lacks in long-term observational buoyancy flux products and model's capability to resolve mesoscale eddies, our current study focuses solely on the terms of diapycnal mixing and wind-driven Ekman transport. In the future when more observational information and decent models are available, more deep studies on the contributions of buoyancy and mesoscale eddies can reveal more physical mechanisms.

Supplementary Text 4: More analyses of GMOC's fingerprints on historic volcanic events

Previous study [34] used a global mean SST (GMSST) residual (GMSSTr) index (a quadratic fit of GMSST is removed) to show the responses of observed GMSST on Mt. Agung (1963), Mt. Chichon (1982) and Mt. Pinatubo (1991) events, three major volcanic eruptions in the second half of the 20th century. They found that the cold GMSSTr anomalies caused by the Agung and Pinatubo volcanic eruptions have a long and profound influence on GMSST since they happen to have occurred at the descending period of a multidecadal GMSST variation. Particularly, the Pinatubo volcanic eruption was the 2nd largest event in the 20th century and the 8th in history [35], and it had even more profound impacts on global climate compared to the Agung

volcanic eruption. Nevertheless, the Chichon volcanic eruption occurred in the ascending period of a GMSST decadal variation and it only produced a short period of cold abnormality for GMSST.

Following that, we first define the global mean sea surface temperature residual (GMSSTr) to identify the SST's fingerprint on prominent volcanic eruption events. The GMSST is an area-weighted mean of 8-year low-passed SST anomalies between 40°S and 60°N and the GMSSTr is obtained by removing the quadratic fit from the GMSST. Then we define a quantity of North Atlantic SST residual (NASSTr) to examine the relationship of NASST anomaly and NADW. The calculation of NASSTr is the same as that of GMSSTr but for only limited in the Northern Atlantic Ocean over the area of 55°–35° W and 45°–65° N.

Supplementary Fig. 26a shows the 8-year low-passed GMSSTr of CDA estimates and the observation which is consistent with Liguori et al. [34]. Affected by the volcanic eruptions of Mt. Agung (1963), Mt. Chichon (1982) and Mt. Pinatubo (1991), the global mean sea surface temperature anomaly (SSTA) showed a significant downward trend. This phenomenon shows consistent manifestations in CDA estimates and OBS.

Supplementary Fig. 27 shows the distributions of SSTA during the periods of 1963–1965, 1982–1985 and 1991–1995 for CESM-CDA and CM2-CDA. Similar with the observation of ERSSTv5 (Extended Reconstructed Sea Surface Temperature version 5) in **Figs. 3a–c**, the global SST cooling produced by the eruption of Mt. Agung (1963) mainly occurred in the Southern Hemisphere. In the high-latitude areas of the North Atlantic, the SSTA shows a positive anomaly. This weakens the cooling and sinking of seawater related to local radiation, reduces the thickness of NADW, and leads to a decrease in the corresponding NADW index. However, the SST cooling caused by the eruptions of Mt. Chichon (1982) and Mt. Pinatubo (1991) is mainly in the Atlantic Ocean and the western Pacific Ocean. In the high-latitude waters of the North Atlantic, there are strong negative anomalies. **Supplementary Fig. 26b** further confirms the different impacts of the three volcanic eruption events on the high-latitude regions of the North Atlantic. The continuous decrease in the NASSTr of Mt. Chichon (1982) and Mt. Pinatubo (1991) corresponds to the continuous increase in NADW, which reaches its maximum around 1997.

Supplementary Fig. 28 shows the CDA-estimated AMMC anomaly distributions during 1963–1965 and 1991–1995 corresponding to Agung and Pinatubo events, respectively. It can be observed that during the period from 1961 to 1966, the amplitude of the SAM index fluctuated within a wide range. In contrast, between 1980 and 2000, its amplitude is notably small. This variation corresponds to the cooling of the SST in the Southern Ocean during the relevant event. Conversely, the amplitude of the NAO is relatively small from 1961 to 1966 but became considerably larger from 1980 to 2000. Notably, there is a distinct upward trend in the NAO index

following the eruptions of Chichon and Pinatubo. The surface buoyancy flux anomalies in the North Atlantic, as simulated by CESM-CDA and CM2-CDA, exhibit a high degree of spatial similarity following the three major volcanic eruptions (**Supplementary Fig. 29)**. This convergence in the buoyancy flux response across the two CDA experiments indicates a consistent representation of the ocean-atmosphere adjustment to volcanic forcing within the assimilation framework.

Supplementary References

1. Sun Y, and Zhou TJ. How does El Niño affect the interannual variability of the boreal summer Hadley circulation? *J. Clim.*, **27**, 2622–2642, (2014). https://doi.org/10.1175/JCLI-D-13-00277.1

2. Jiang, Y., Lu, L., Zhang, S., Zhu, C., Gao, Y., Lin, Z., Wan, L., Li, M., Yu, X., Wu, L. & Lin, X. An assessment of convergence of climate reanalyses from two coupled data assimilation systems with identical high-efficiently filtering. *J. Clim.*, **37**(16), 4153–4181 (2024). https://doi.org/10.1175/JCLI-D-23-0423.1

3. Zhang, S., Harrison, M. J., Rosati, A. & Wittenberg, A. System design and evaluation of coupled ensemble data assimilation for global oceanic climate studies. *Mon. Wea. Rev.*, **135**, 3541–3564 (2007). https://doi.org/10.1175/MWR3466.1

4. Zhang, S., Rosati, A., & Harrison, M. J. Detection of multidecadal oceanic variability by ocean data assimilation in the context of a "perfect" coupled model. *J. Geo. Res.*, **114**(C12), C12018, (2009). https://doi.org/10.1029/2008JC005261

5. Chang Y, Zhang S, Rosati A, Delworth T, and Stern W. An assessment of oceanic variability for 1960–2010 from the GFDL ensemble coupled data assimilation. *Clim. Dyn.*, **40**, 775–803, (2013). https://doi.org/10.1007/s00382-012-1412-2

6. Huang RX, Wang W, and Liu LL. Decadal variability of wind-energy input to the world ocean. *Deep Sea Res. Part II: Topical Studies in Oceanography*, **53**(1), 31–41, (2006). https://doi.org/10.1016/j.dsr2.2005.11.001

7. Cronin MF, Gentemann CL, Edson J, et al. Air-sea fluxes with a focus on heat and momentum. *Front. Mar. Sci.*, **6**, 430, (2019). doi:10.3389/fmars.2019.00430

8. Ma, X., Jing, Z., Chang, P., Liu, X., Montuoro, R., Small, R. J., Bryan, F. O., Greatbatch, R. J., Brandt, P., Wu, D., Lin, X. & Wu, L. Western boundary currents regulated by interaction between ocean eddies and the atmosphere. *Nature*, **535**, 533–537 (2016). https://doi.org/10.1038/nature18640

9. Pokhrel, S., Dutta, U., Rahaman, H., Chaudhari, H., Hazra, A. & Saha, S. K., Veeranjaneyulu, C. Evaluation of different heat flux products over the tropical Indian Ocean. *Earth Space Sci.* **7**, e2019EA000988 (2020). https://doi.org/10.1029/2019EA000988

10. Mayer, J., Haimberger, L. & Mayer, M. A quantitative assessment of air–sea heat flux trends from ERA5 since 1950 in the North Atlantic basin. *Earth Syst. Dyn.* **14**, 1085–1105 (2023). https://doi.org/10.5194/esd-14-1085-2023

11. Su, F., Fan, R., Yan, F., Meadows, M., Lyne, V., Hu, P., Song, X., Zhang, T., Liu, Z., Zhou, C., Pei, T., Yang, X., Du, Y., Wei, Z., Wang, F., Qi, Y. & Chai, F. Widespread global disparities between modelled and observed mid-depth ocean currents. *Nat. Commun.*, **14**, 2089 (2022). https://doi.org/10.1038/s41467-023-37841-x

12. Ollitrault, M. & Rannou, J. P. ANDRO: An Argo-based deep displacement dataset. *J. Atmos. Ocean Technol.*, **30**, 759–788 (2013). https://doi.org/10.1175/JTECH-D-12-00073.1

13. Buckley MW, and Marshall J. Observations, inferences, and mechanisms of Atlantic meridional overturning circulation variability: A review. *Rev. Geo.*, **54**, 5–63, (2016). https://doi.org/10.1002/2015RG000493

14. Lu, L., Zhang, S., Yeager, S. G., Danabasoglu, G., Chang, P., Wu, L., Lin, X., Rosati, A., & Lu, F. Impact of coherent ocean stratification on AMOC reconstruction by coupled data assimilation with a biased model. *J. Clim.*, **3**, 7319–7334 (2020). https://doi.org/10.1175/JCLI-D-19-0735.1

15. Liu, Z., Gu, S., Zou, S., Zhang, S., Yu, Y. & He, C. Wind-steered eastern pathway of the Atlantic meridional overturning circulation. *Nat. Geosci.*, **17**, 353–360 (2024). https://doi.org/10.1038/s41561-024-01407-3

16. Köhl, A. & Stammer, D. Variability of the meridional overturning in the North Atlantic from the 50-year GECCO state estimation. *J. Phys. Oceanogr.*, **38**, 1913–1930 (2008). https://doi.org/10.1175/2008JPO3775.1

17. Frajka-Williams, E., Ansorge, I. J., Baehr, J., et al. Atlantic meridional overturning circulation: Observed transport and variability. Front. Mar. Sci., 6, 260 (2019). https://doi:10.3389/fmars.2019.00260

18. Wett, S., Rhein, M., Kieke, D., Mertens, C. & Moritz, M. Meridional connectivity of a 25-year observational AMOC record at 47°N. *Geo. Res. Lett.*, **50**, e2023GL103284 (2023). https://doi.org/10.1029/2023GL103284

19. Good, S. A., Martin, M. J. & Rayner, N. A. EN4: Quality controlled ocean temperature and salinity profiles and monthly objectively analyses with uncertainty estimates. *J. Geophys. Res. Oceans.*, **118**, 6704–6716 (2013). https://doi.org/10.1002/2013JC009067

20. Stommel H. Thermohaline convection with two stable regimes of flow. *Tellus*, **13**(2), 224–230, (1961). https://doi.org/10.1111/j.2153-3490.1961.tb00079.x

21. Manabe S. Climate and the ocean circulation: I. The atmospheric circulation and the hydrology of the Earth's surface. *Mon. Wea. Rev.*, **97**(11), 739–774 (1969). https://doi.org/10.1175/1520-0493(1969)097<0739:CATOC>2.3.CO;2

22. Charney JG, and Drazin PG. Propagation of planetery scale disturbances from the lower into the upper atmosphere. *J. Geophys. Res.*, **66**, 83–109 (1961). https://doi.org/10.1029/JZ066i001p00083

23. Wright, D. G. & Stocker, T. F. A zonally averaged ocean model for the thermohaline circulation. Part I: model development and flow dynamics, *J. Phys. Oceanogr.*, **21**(12), 1713–1724 (1991). https://doi.org/10.1175/1520-0485(1991)021<1713:AZAOMF>2.0.CO;2

24. Gnanadesikan, A. A simple predictive model for the structure of theoceanic pycnocline. *Science*, **283**, 2077–2079 (1999). https://www.science.org/doi/10.1126/science.283.5410.2077

25. Zhang, S., Xu, S., Fu, H., et al. Toward Earth system modeling with resolved clouds and ocean submesoscales on heterogeneous many-core HPCs. *Natl. Sci. Rev.*, **10**, nwad069 (2023). https://doi:10.1093/nsr/nwad069

26. Zhang, S. & Rosati, A. An inflated ensemble filter for ocean data assimilation with a biased coupled GCM. *Mon. Wea. Rev.*, **138**, 3905–3931 (2010). DOI: 10.1175/2010MWR3326.1

27. Griffies SM, Danabasoglu G, Durack PJ, et al. OMIP contribution to CMIP6: experimental and diagnostic protocol for the physical component of the Ocean Model Intercomparison Project. *Geo. Mod. Dev.*, **9**(9), 3231–3296, (2016). https://doi.org/10.1175/1520-0469(1950)007<0080:OTWDOC>2.0.CO;2

28. Jayne SR, Roemmich D, Zilberman N, Riser SC, Johnson KS, Johnson GC, and Piotrowicz SR. The Argo program: present and future. *Oceanography*, **30**(3), 18–28, (2017). https://doi.org/10.5670/oceanog.2017.213

29. Donlon CJ, Martin M, Stark J, Robert-Jones J, Fiedler E, and Wimmer W. The operational sea surface temperature and sea ice analysis (OSTIA) system. *Remote Sens Environ*, **116**, 140–158, (2012). https://doi.org/10.1016/j.rse.2010.10.017

30. Abraham JP, Baringer M, Bindoff NL, et al. A review of global ocean temperature observations: Implications for ocean heat content estimates and climate change. *Rev. Geo.*, **51**(3), 450–483, (2013). https://doi.org/10.1002/rog.20022

31. Hurrell JW, and Deser C. North Atlantic climate variability: The role of the North Atlantic Oscillation. *J. Mar. Syst.*, **78** (1), 28–41, (2009). https://doi.org/10.1016/j.jmarsys.2009.11.002

32. Trenberth K-E, and Hoar T-J. The 1990-1995 El Nino-Southern Oscillation event longest on record. *Geo. Res. Lett.*, **23**, 57–60, (1996). https://doi.org/10.1029/95GL03602

33. Marshall GJ. Trends in the Southern Annular Mode from observations and reanalyses. *J. Clim.*, **16**, 4134–4143, (2003). https://doi.org/10.1175/1520-0442(2003)016<4134:TITSAM>2.0.CO;2

34. Liguori, G., McGregor, S., Arblaster, J. M., Singh, M. S. & Meehl, G. A. A joint role for forced and internally-driven variability in the decadal modulation of global warming. *Nat. Commun.*, **11**, 3827 (2020). https://doi.org/10.1038/s41467-020-17683-7

35. Ghose, T. The 12 biggest volcanic eruptions in recorded history. Live Science, Future US, Inc. Full 7th Floor, 130 West 42nd Street, New York, NY 10036 (2023). https://www.livescience.com/planet-earth/volcanos/the-12-biggest-volcanic-eruptions-in-recorded-history

36. Nikurashin, M. & Vallis, G. A theory of the interhemispheric meridional overturning circulation and associated stratification. *J. Phys. Oceanogr.*, **42**(10), 1652–1667 (2012). https://doi.org/10.1175/JPO-D-11-0189.1

37. Zhang, S. Impact of observation-optimized model parameters on decadal predictions: Simulation with a simple pycnocline prediction model. *Geo. Res. Lett.*, 38, L02702 (2011). https://doi.org/10.1029/2010GL046133

Supplementary Fig. 1–30:

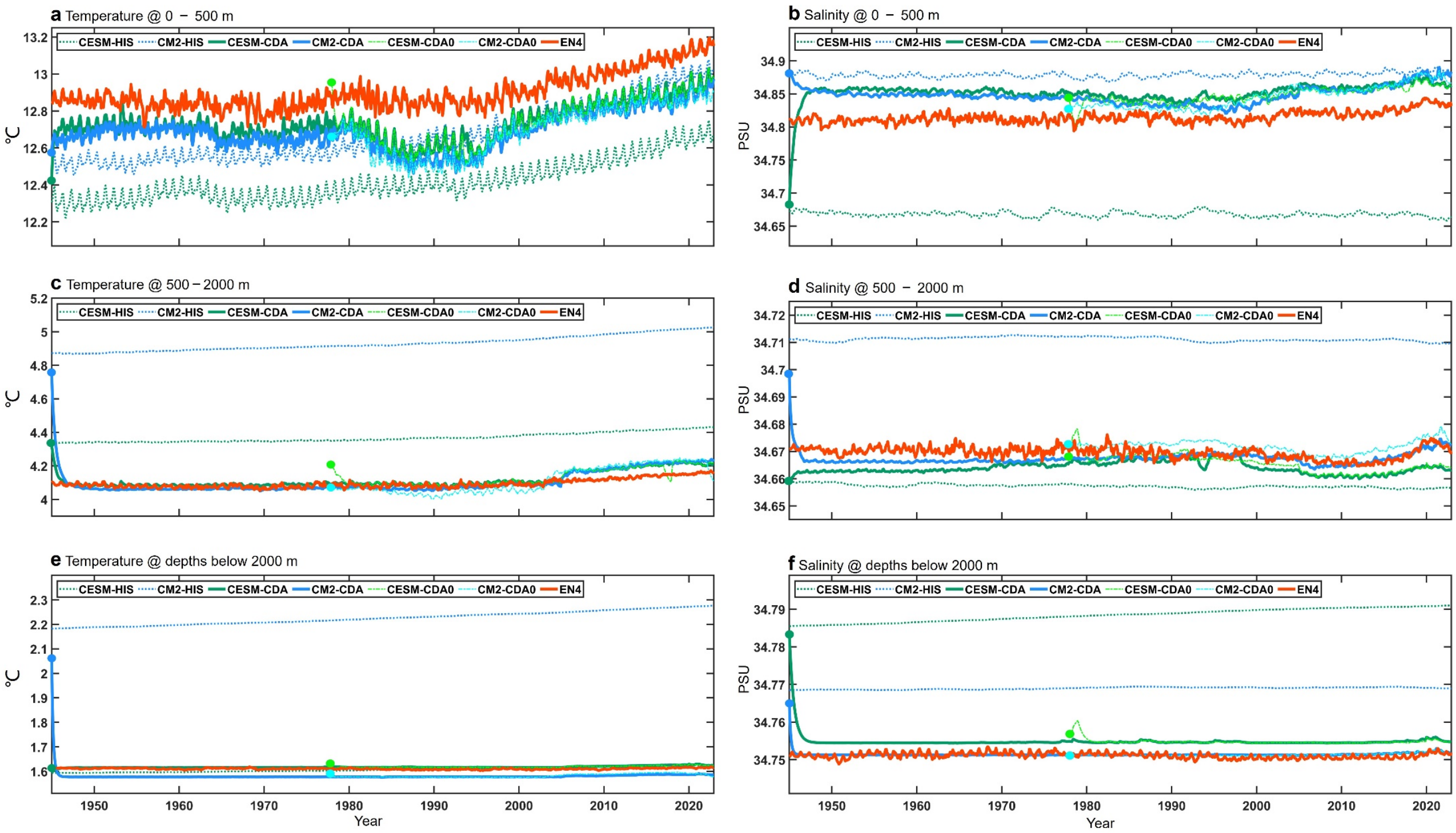

Supplementary Fig. 1 | Convergent ocean temperature and salinity in coupled data assimilation (CDA) estimates. **a–b**, Timeseries of **a**) mean temperature (unit: °C) and **b**) salinity (unit: PSU) over the area 60° S–60° N at 0–500 m ocean depth. The dashed light-green and light-blue lines represent other CDA experiments (CESM-CDA0 and CM2-CDA0) for the period 1978–2022 [2]. The restart initial condition (denoted by a light-green dot) of CESM-CDA0 is the coupled model state at 00:00 UTC on 01-01-1978 of the historical model simulation running from 01-01-1869. The restart initial condition (denoted by a light-blue dot) of CM2-CDA0 is the previous assimilation coupled state at 00:00 UTC on 01-01-1978 [5]. **c–d**, Same as panels **a–b** but for 500–2000 m ocean depth. **e–f**, Same as panels **a–b** but for the ocean depths below 2000 m. All panels are plotted using MATLAB R2025a.

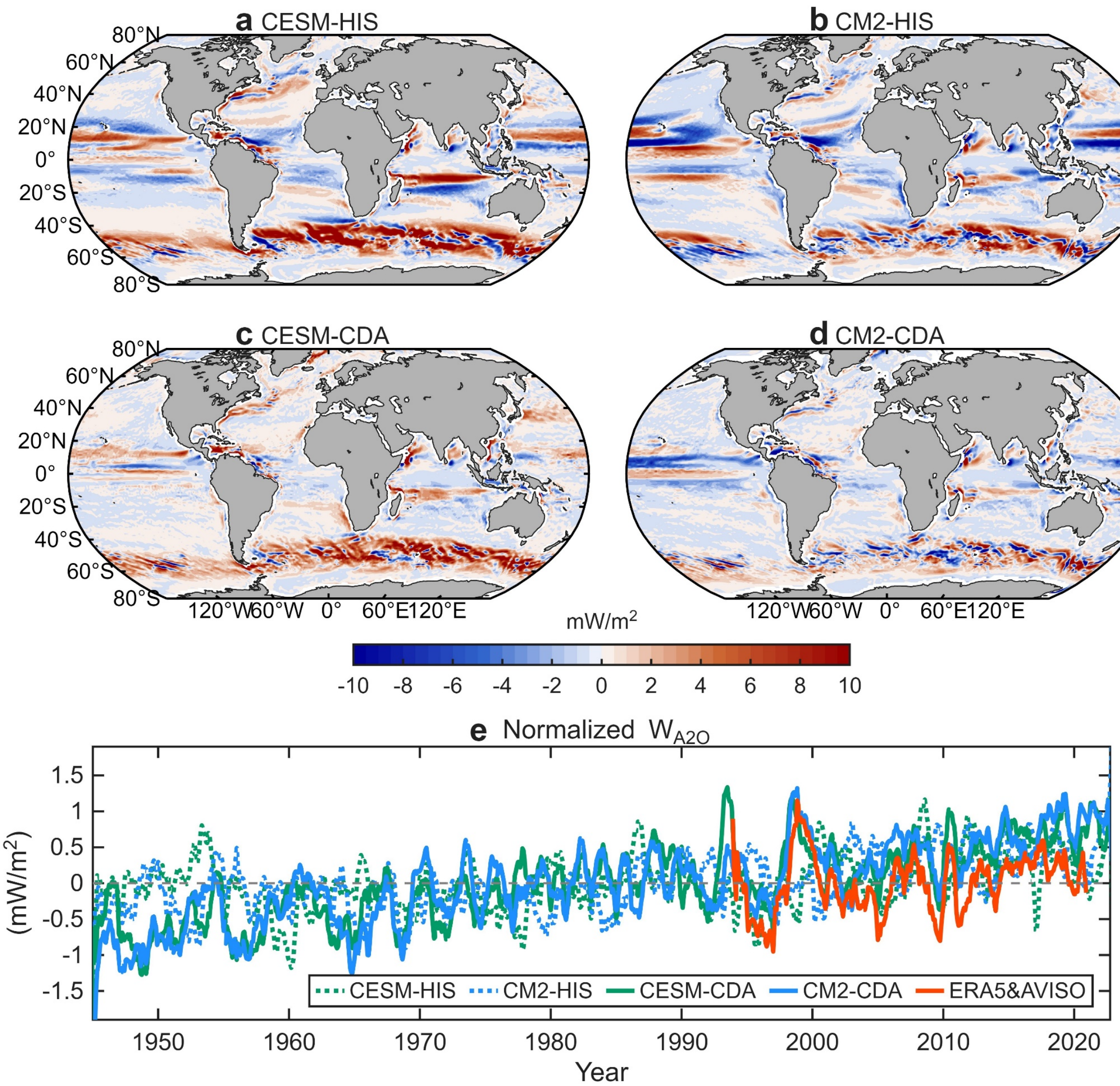

Supplementary Fig. 2 | Evaluation of atmospheric work exerted on the oceans (W_{A2O}). **a–d**, Error distributions of W_{A2O} for CESM-HIS, CM2-HIS, CESM-CDA, and CM2-CDA, respectively, against the observed product derived from ERA5 and AVISO over the period 1993–2020 (unit: $mW{\cdot}m^{-2}$). **e**, Normalized global mean timeseries of W_{A2O} in CESM-HIS (green dotted line), CM2-HIS (blue dotted line), CESM-CDA (green solid line), CM2-CDA (blue solid line), and the observed product derived from ERA5 reanalysis and AVISO (red solid line). Generated by MATLAB R2025a.

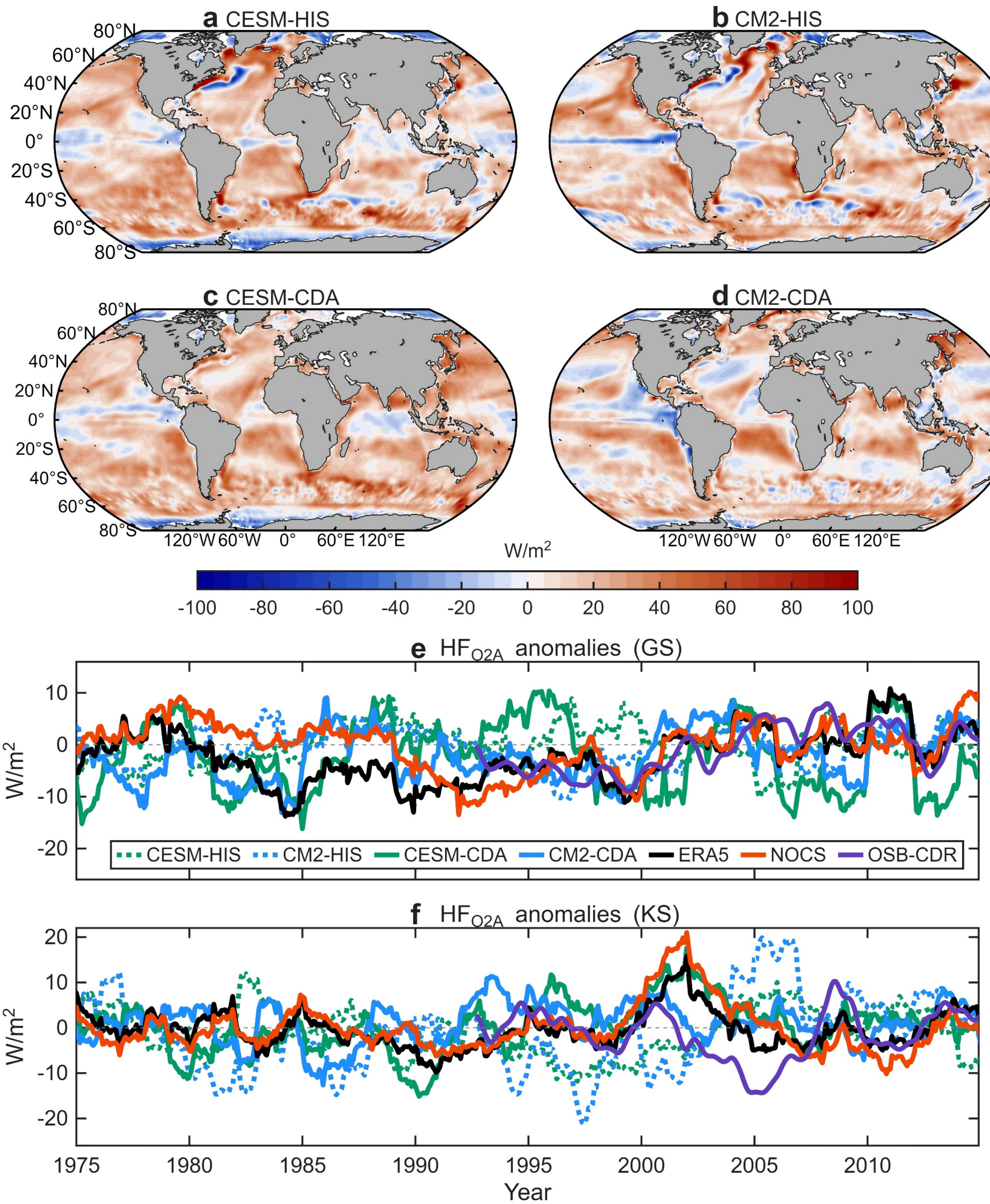

Supplementary Fig. 3 | Evaluation of upward ocean-to-atmosphere heat flux (HF_{O2A}) in coupled data assimilation (CDA). **a–d**, Spatial distributions of climatological HF_{O2A} bias for CESM-HIS, CM2-HIS, CESM-CDA, and CM2-CDA, respectively, validated against the NOCS observational dataset. **e–f**, Timeseries of 24-month running-mean HF_{O2A} anomalies (unit: $W{\cdot}m^{-2}$) averaged over the Gulf Stream (80°W–40°W, 30°N–50°N) and Kuroshio (140°E–160°E, 32°N–50°N). Lines denote CESM-HIS (green dotted), CM2-HIS (blue dotted), CESM-CDA (green solid), CM2-CDA (blue solid), ERA5 (black solid), NOCS (red solid), and OSB-CDR (purple solid). Generated by MATLAB R2025a.

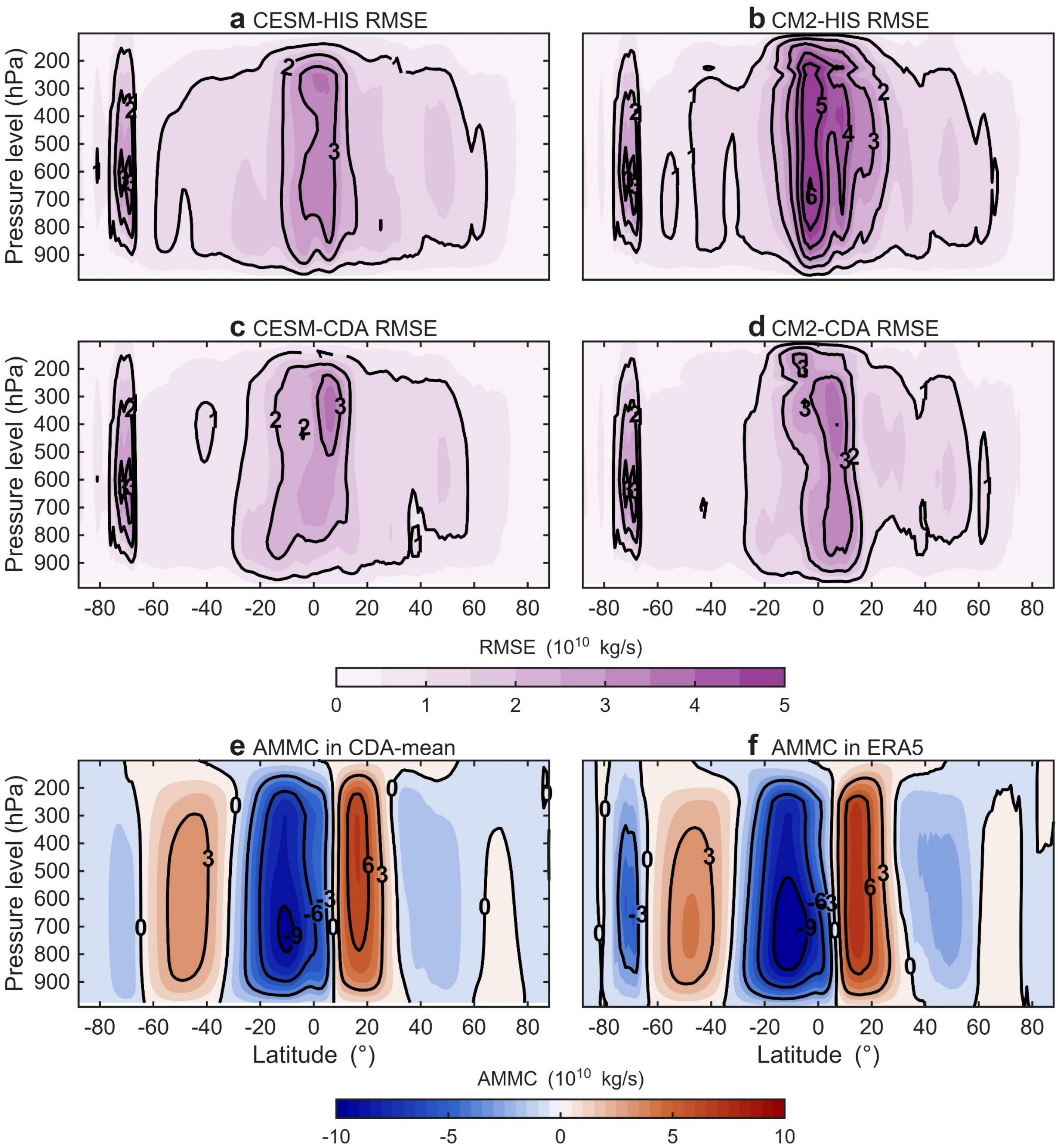

632

633 **Supplementary Fig. 4 |** Convergent atmospheric mean meridional circulation (AMMC) mean state in coupled data assimilation (CDA) estimates. **a–d**, Root-mean-square error (RMSE) distributions of AMMC (unit: 10^{10} kg·s^{-1}) for the model simulations CESM-HIS and CM2-HIS, and the CDA results CESM-CDA and CM2-CDA, verified against ERA5. **e–f**, Mean states of AMMC in CDA mean and ERA5, respectively. Generated by MATLAB R2025a.

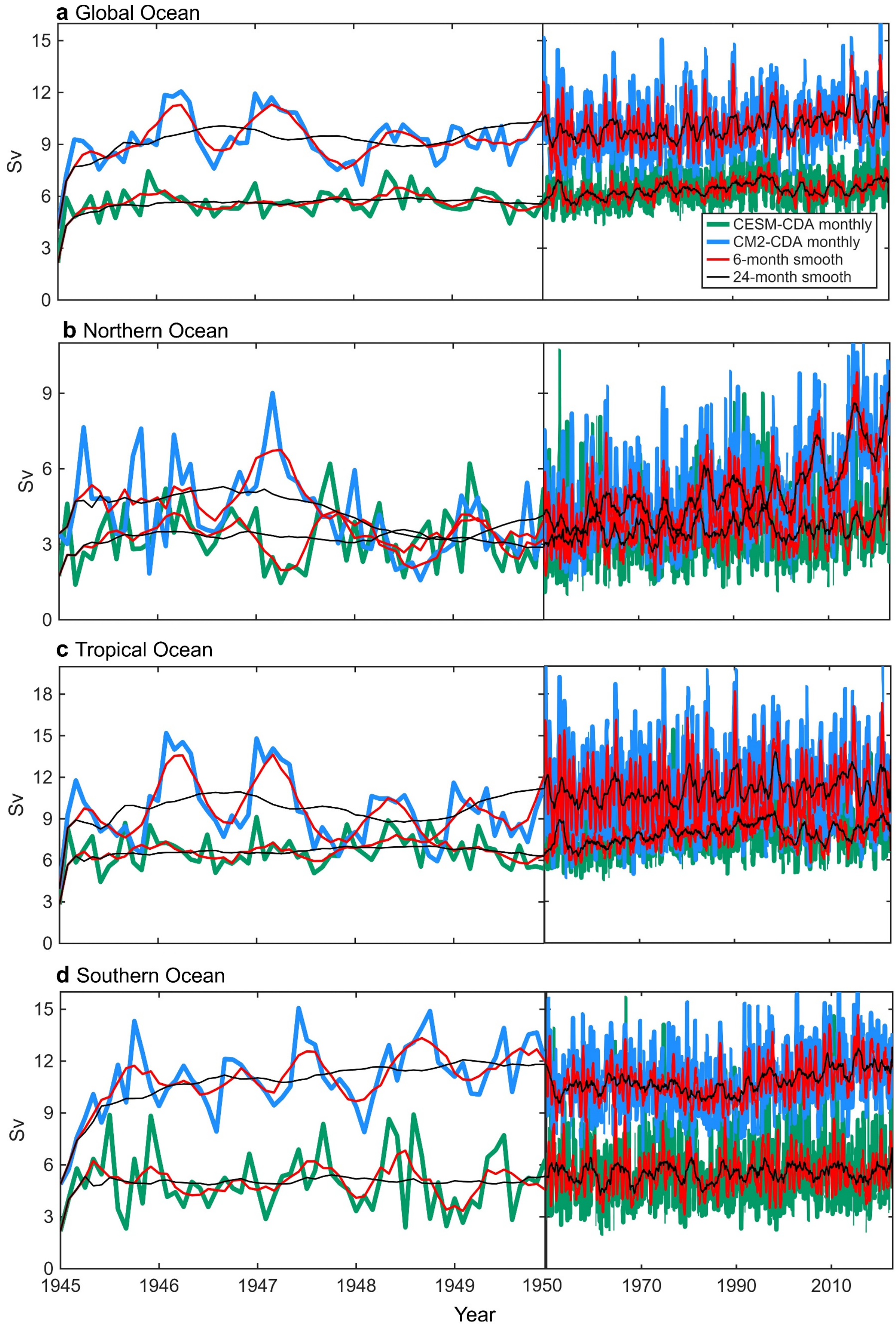

Supplementary Fig. 5 | Time series of root-mean-square differences (RMSD, Sv) of the Global Meridional Overturning Circulation (GMOC) below 2000 m between the CDA experiments and their corresponding historical model simulations (HIS): **a**, Global Ocean, **b**, Northern Ocean, **c**, Tropical Ocean and **d**, Southern Ocean. Monthly data are shown for CESM-CDA (green) and CM2-CDA (blue). The red and black lines represent 6-month and 24-month running means, respectively, to highlight low-frequency variability. Note that the period of 1945–1950 is zoomed in for visualization convenience. Generated by MATLAB R2025a.

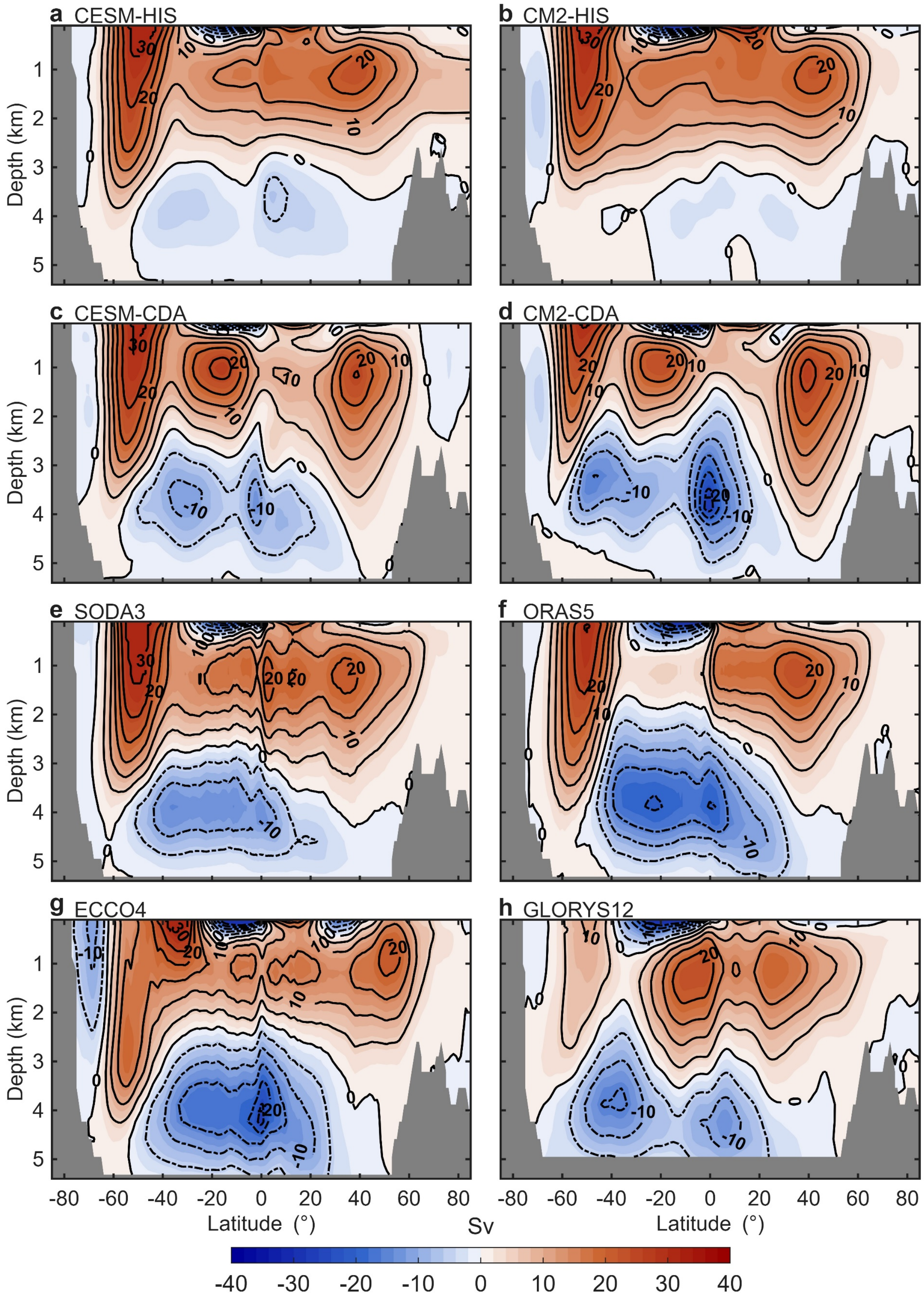

Supplementary Fig. 6 | a–h, Stream functions (unit: Sv = 10^6 $m^3 \cdot s^{-1}$) of GMOCs for historical simulations (1950–2022) of CESM (a) and CM2 (b) (denoted as CESM-HIS and CM2-HIS), coupled data assimilation (CDA) results (1950–2022) with CESM (c) and CM2 (d) models (denoted as CESM-CDA and CM2-CDA), as well as the ocean reanalysis products SODA3 (1980–2019) (e), ORAS5 (1958–2022) (f), ECCO4 (1992–2015) (g) and GLORYS12 (1993–2016) (h). Generated by MATLAB R2025a.

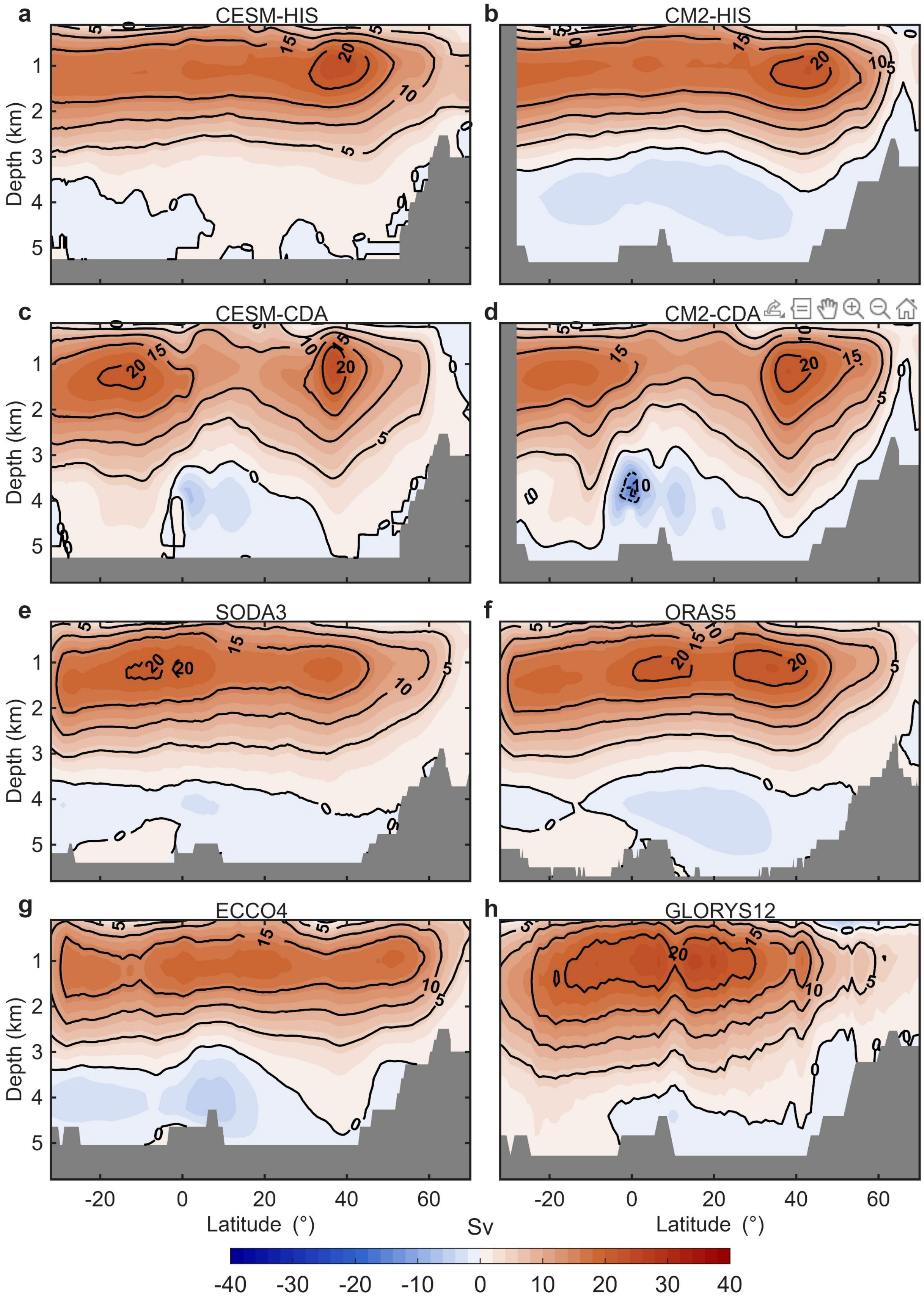

Supplementary Fig. 7 | a–h, Temporal mean AMOC (unit: Sv) in CESM-HIS (a), CM2-HIS (b), CESM-CDA (c), CM2-CDA (d), SODA3 (e), ECCO4 (f), ORAS5 (g) and GLORYS12 (h), respectively. Generated by MATLAB R2025a.

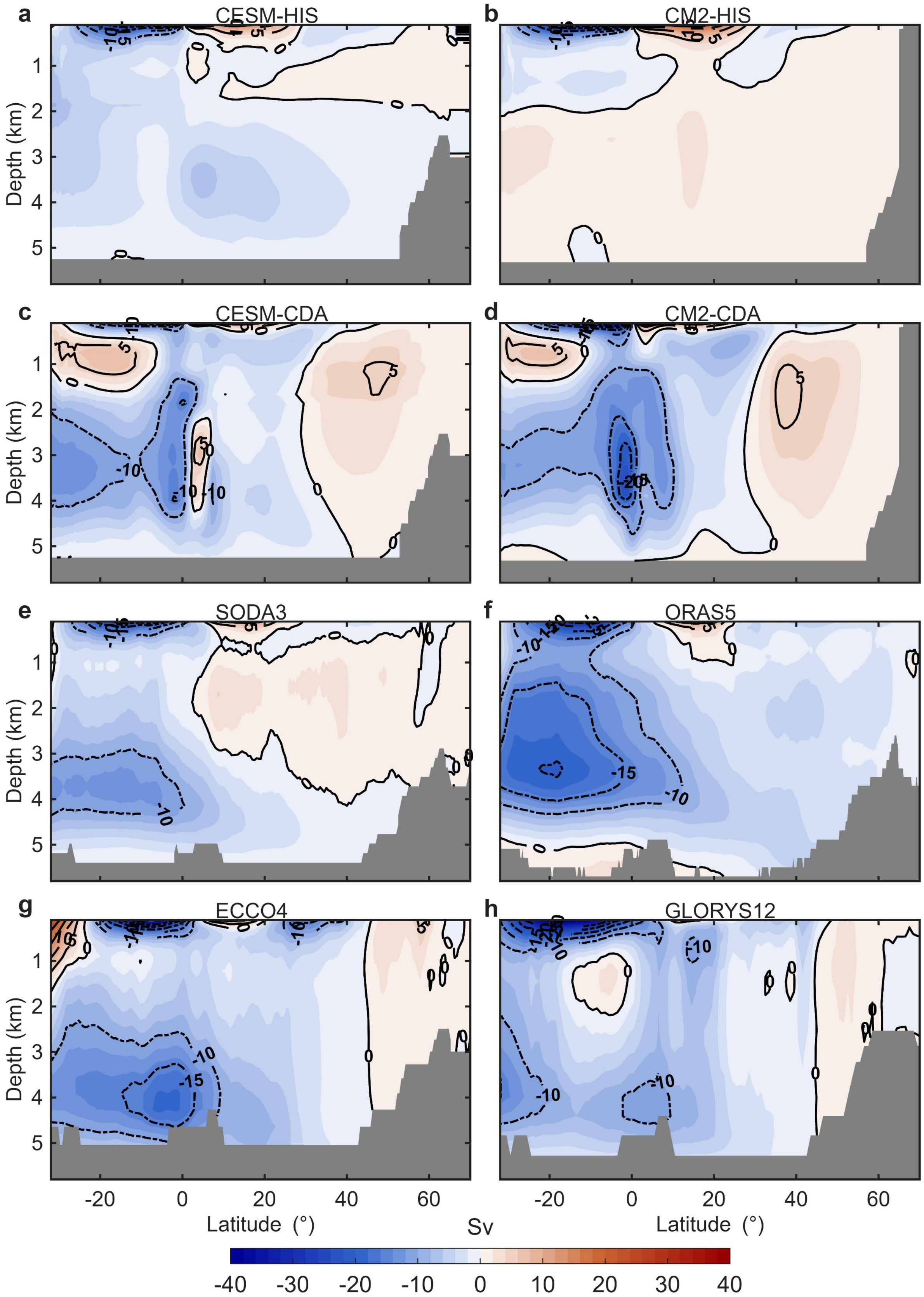

Supplementary Fig. 8 | a–h, Temporal mean Indo-Pacific meridional overturning circulations (IPMOC) (unit: Sv) in CESM-HIS (a), CM2-HIS (b), CESM-CDA (c), CM2-CDA (d), SODA3 (e), ECCO4 (f), ORAS5 (g) and GLORYS12 (h), respectively. Generated by MATLAB R2025a.

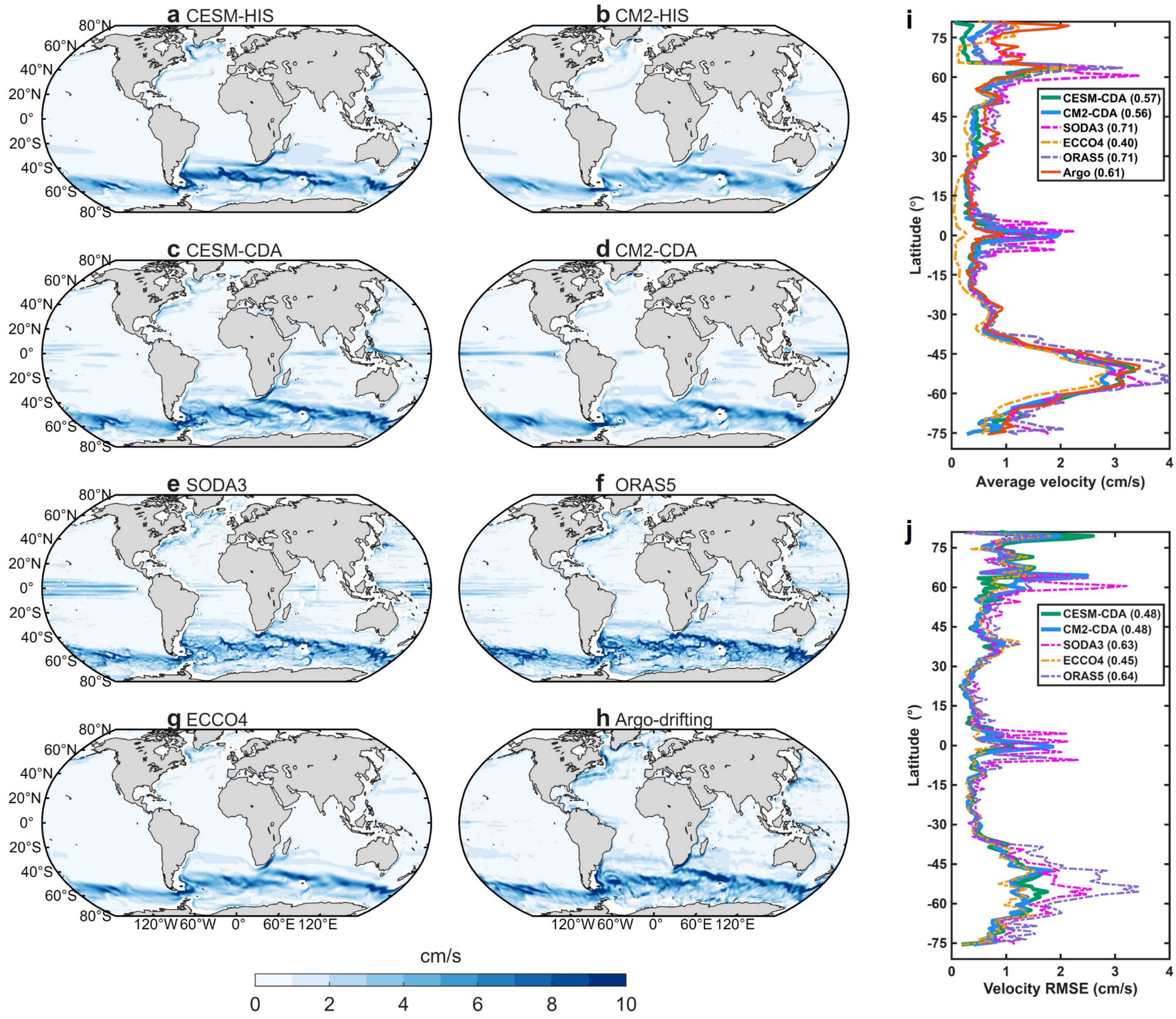

Supplementary Fig. 9 | a–b, Argo period (2000–2023) temporal mean distributions of 950–1150 m ocean current velocities (unit: cm·s^{-1}) in CESM-HIS and CM2-HIS, respectively. **c–g**, Same as **a–b** but for CESM-CDA, CM2-CDA, SODA3, ECCO4 and ORAS5, respectively. **h**, Argo-drifting estimated ocean current velocities (unit: cm·s^{-1}) for 950–1150 m ocean depth [9]. **i**, The distribution of average ocean current velocities with latitude at 950–1150 m. **j**, Same as panel **i**, but for root mean square errors (RMSEs). Generated by MATLAB R2025a.

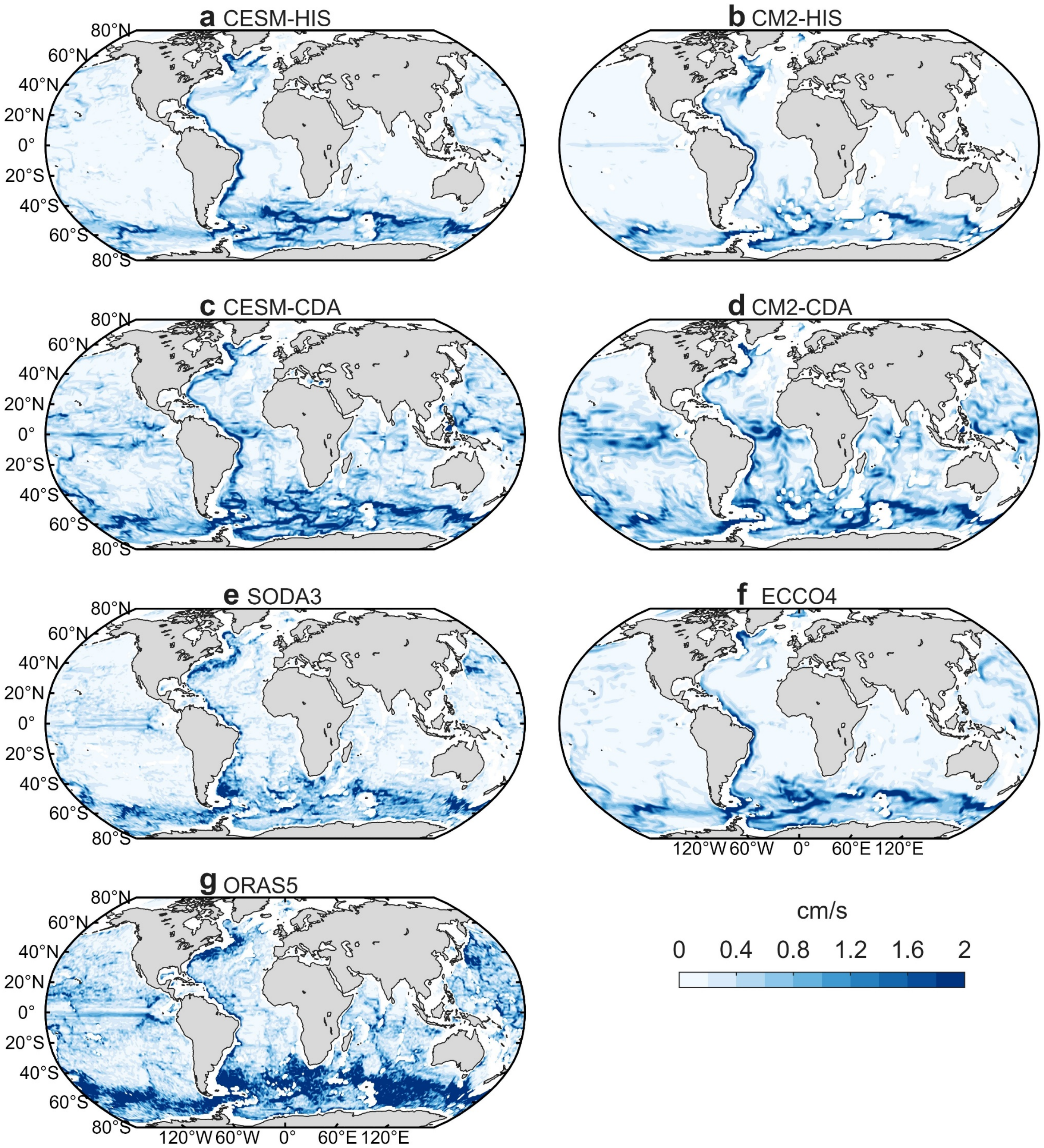

Supplementary Fig. 10 | Convergent deep-ocean current velocities in coupled data assimilation (CDA) estimates. **a–g**, Temporal mean distributions of 2500–5000 m ocean current velocities (unit: $cm \cdot s^{-1}$) in CESM-HIS (a), CM2-HIS (b), CESM-CDA (c), CM2-CDA (d), SODA3 (e), ECCO4 (f) and ORAS5 (g), respectively. Generated by MATLAB R2025a.

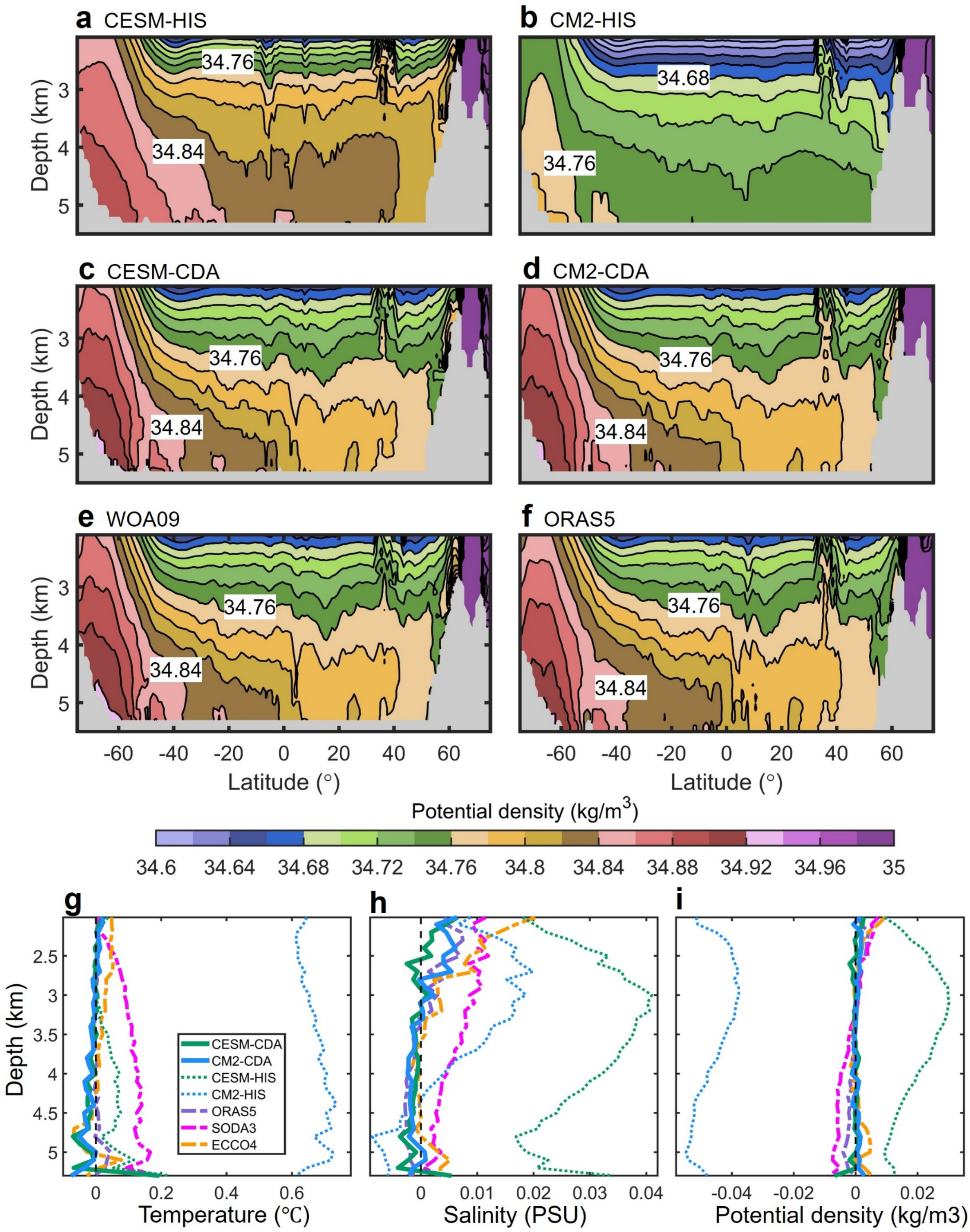

Supplementary Fig. 11 | Evaluation of the convergence of deep ocean in two CDA products. **a–f**, Zonal-mean potential density (kg m⁻³) as a function of latitude and depth (km) for the 2–6 km deep ocean in CESM-HIS (a), CM2-HIS (b), CESM-CDA (c), CM2-CDA (d), ORAS5 (e) and WOA09 (f) observational climatology. The color bar at the bottom shows the range of potential density values, with contour intervals of 0.04 kg m⁻³. Key isopycnals of 34.76 and 34.84 kg m⁻³ are labeled in each panel to highlight the structure of deep-water masses. **g–i**, Vertical profiles of global-mean biases relative to observational references in the 2–6 km deep ocean for Potential temperature (°C) (g), salinity (PSU) (h) and potential density (kg m⁻³) (i). The profiles are shown for CESM-CDA (solid green), CM2-CDA (solid blue), CESM-HIS (dashed green), CM2-HIS (dotted blue), ORAS5 (dashed purple), SODA3 (dashed magenta), and ECCO4 (dashed orange). Generated by MATLAB R2025a.

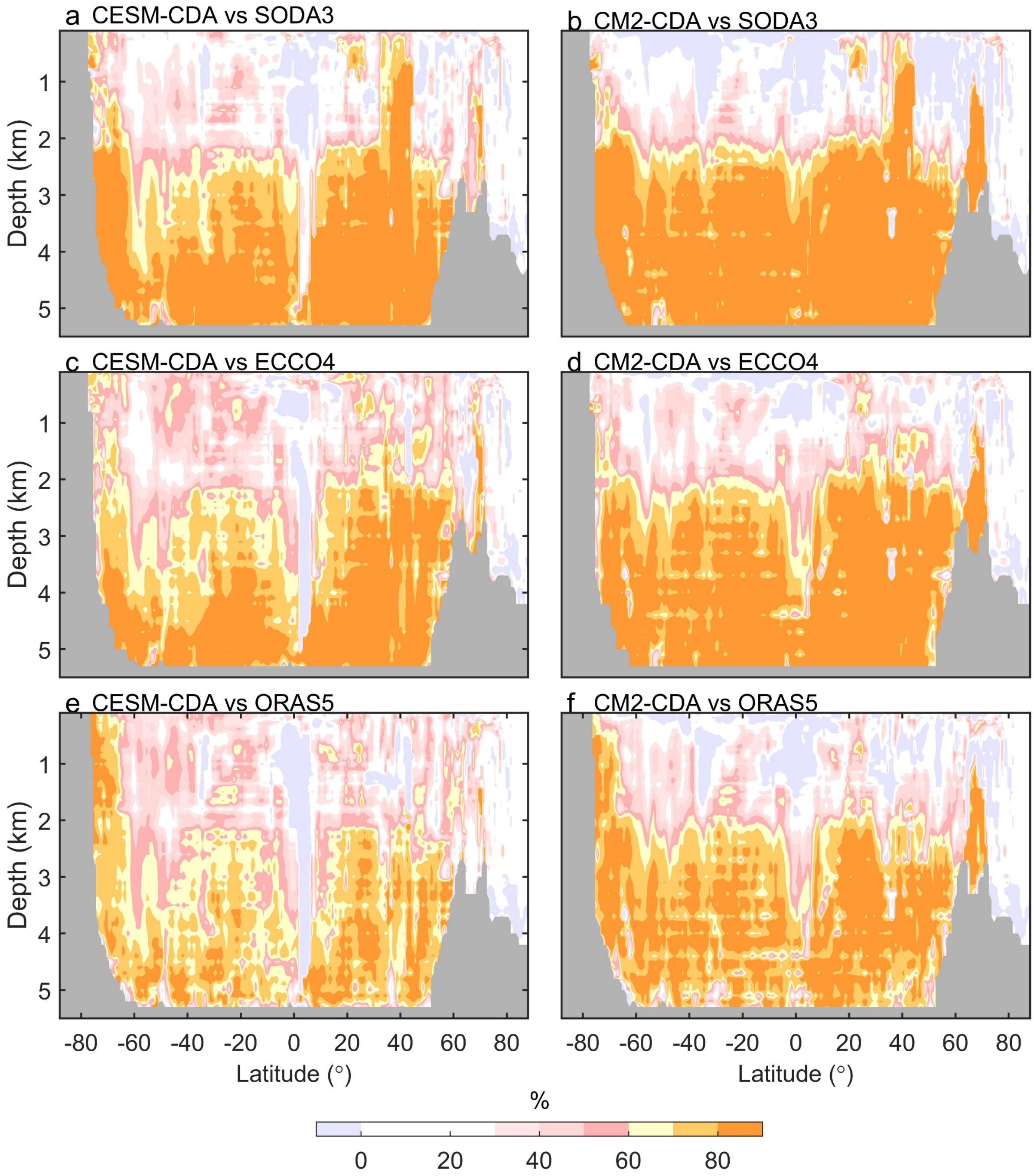

Supplementary Fig. 12 | Coherent ocean stratification in coupled data assimilation (CDA) estimates. **a–f**, Latitudinal distributions of root-mean-square error (RMSE) reduction rates (unit: %) for potential density compared with **a–b**, SODA3, **c–d**, ECCO4, and **e–f**, ORAS5 in CESM-CDA (left panels) and CM2-CDA (right panels), verified against the WOA09. Generated by MATLAB R2025a.

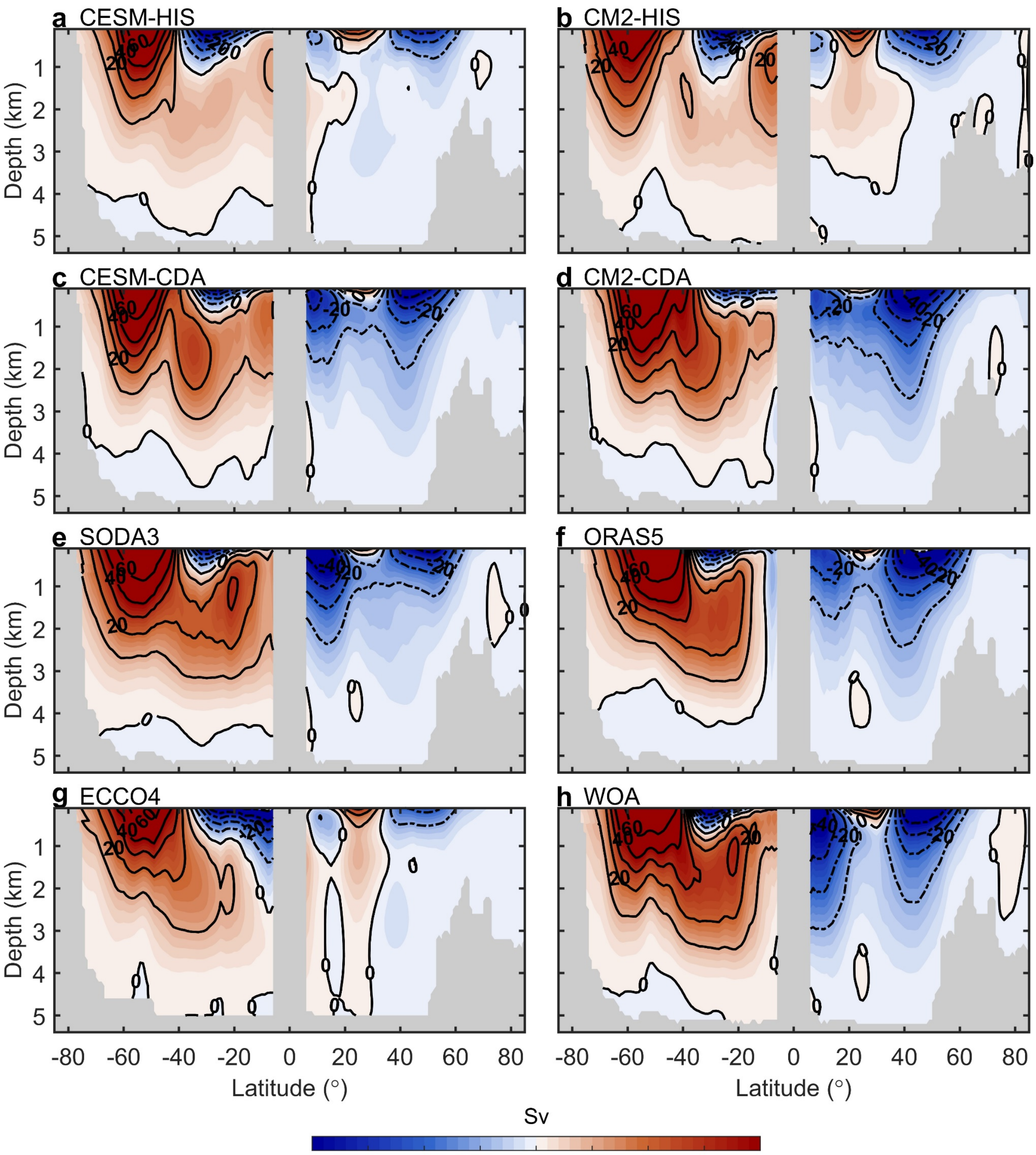

Supplementary Fig. 13 | Geostrophic global meridional overturning circulation (GMOC) mean states of model simulations and assimilation products. **a–h**, Geostrophic GMOC mean states in CESM-HIS, CM2-HIS, CESM-CDA, CM2-CDA, SODA3, ORAS5, ECCO4, and WOA18, respectively. Generated by MATLAB R2025a.

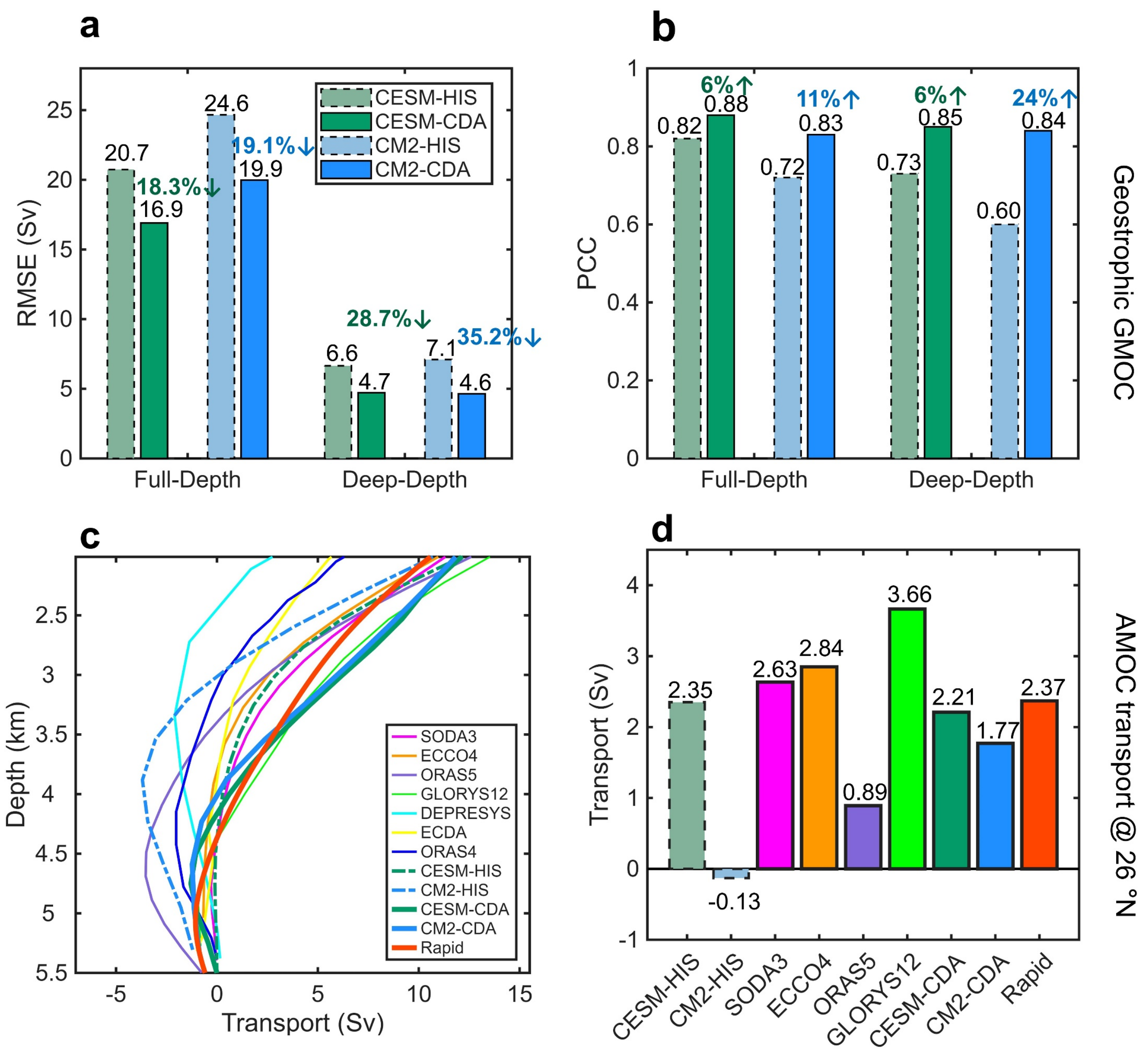

Supplementary Fig. 14 | Evaluation of geostrophic GMOC and AMOC transport in model simulations and CDA as well as reference datasets. a, The RMSE (Sv) of geostrophic GMOC in the full-depth and deep-ocean (below 2000 m) domains for CESM-HIS, CESM-CDA, CM2-HIS, and CM2-CDA. Percentages indicate the relative change in RMSE from the historical simulation (HIS) to the data assimilation (CDA) experiment. **b,** Pattern Correlation Coefficient (PCC) of the geostrophic GMOC in the full-depth and deep-depth domains. Percentages denote the relative improvement in correlation coefficient from HIS to CDA. **c,** Vertical profiles of the Atlantic Meridional Overturning Circulation (AMOC) transport (Sv) at 26°N, focusing on depths below 2 km, from multiple datasets (SODA3, ECCO4, ORAS5, GLORYS12, DEPRESYS, ECDA, ORAS4) and experiments (CESM-HIS, CM2-HIS, CESM-CDA, CM2-CDA), as well as the Rapid observation. **d,** Mean AMOC transport (Sv) at 26°N (averaged over depths below 2 km) for the datasets and experiments shown in **c**. Generated by MATLAB R2025a.

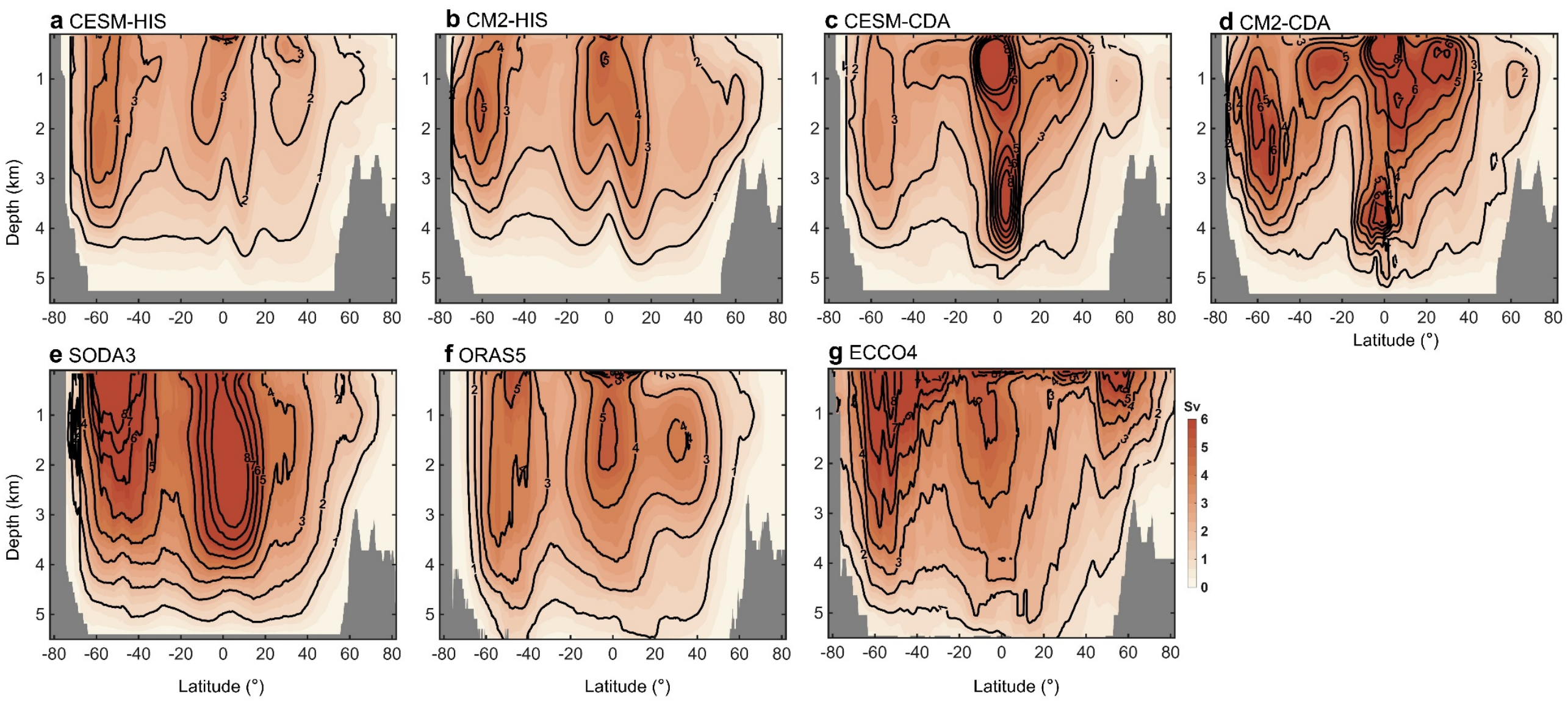

Supplementary Fig. 15 | a–g, Distributions of standard deviations for global meridional overturning circulation (GMOC) stream functions from model simulations CESM-HIS (a), CM2-HIS (b), and assimilation products CESM-CDA (c), CM2-CDA (d), SODA3 (e), ECCO4 (f), ORAS5 (g) (unit: Sv). Generated by MATLAB R2025a.

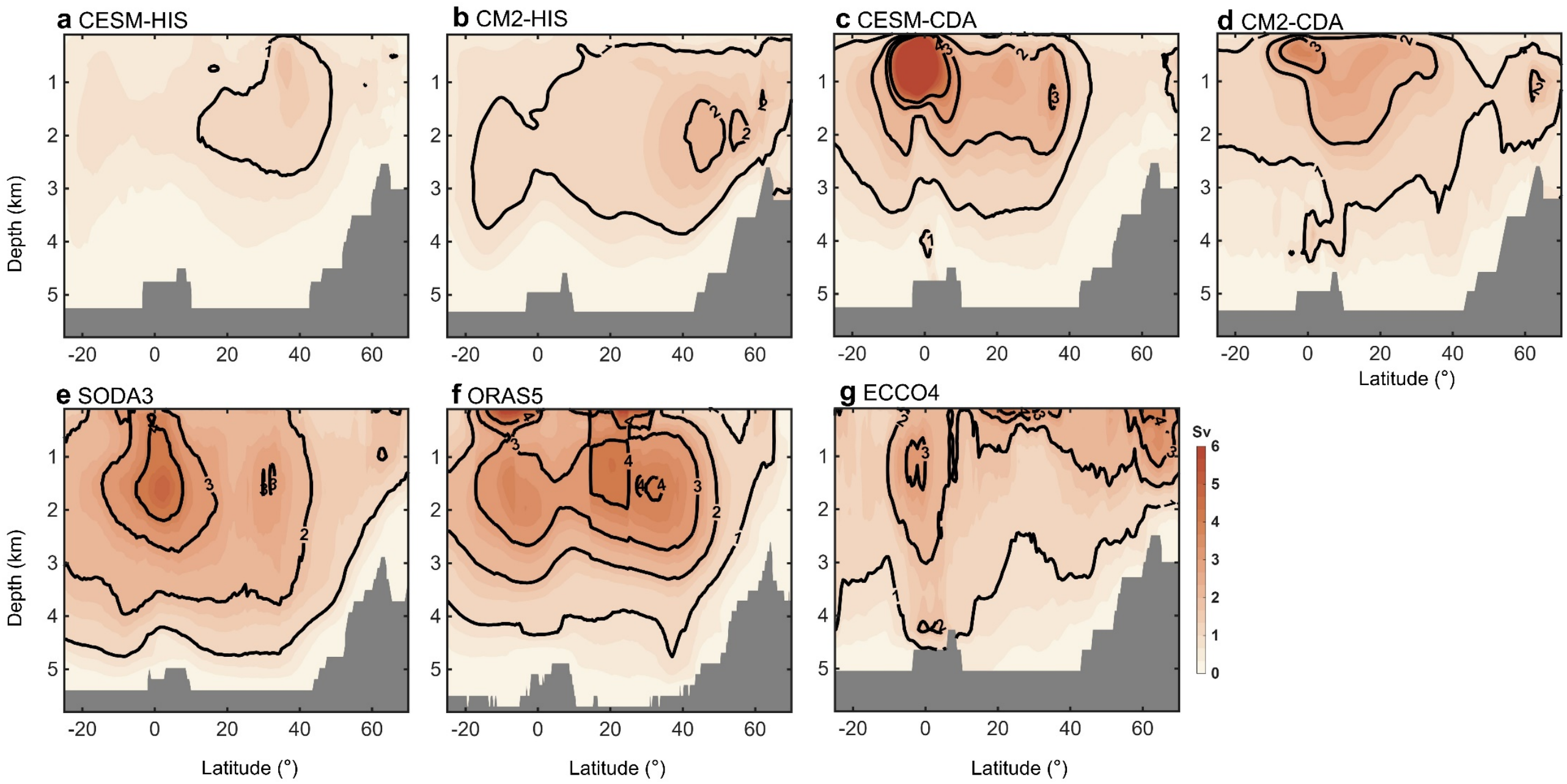

Supplementary Fig. 16 | a–g, Distributions of standard deviations for Atlantic meridional overturning circulation (AMOC) stream functions from model simulations CESM-HIS (a), CM2-HIS (b), and assimilation products CESM-CDA (c), CM2-CDA (d), SODA3 (e), ECCO4 (f), ORAS5 (g) (unit: Sv). Generated by MATLAB R2025a.

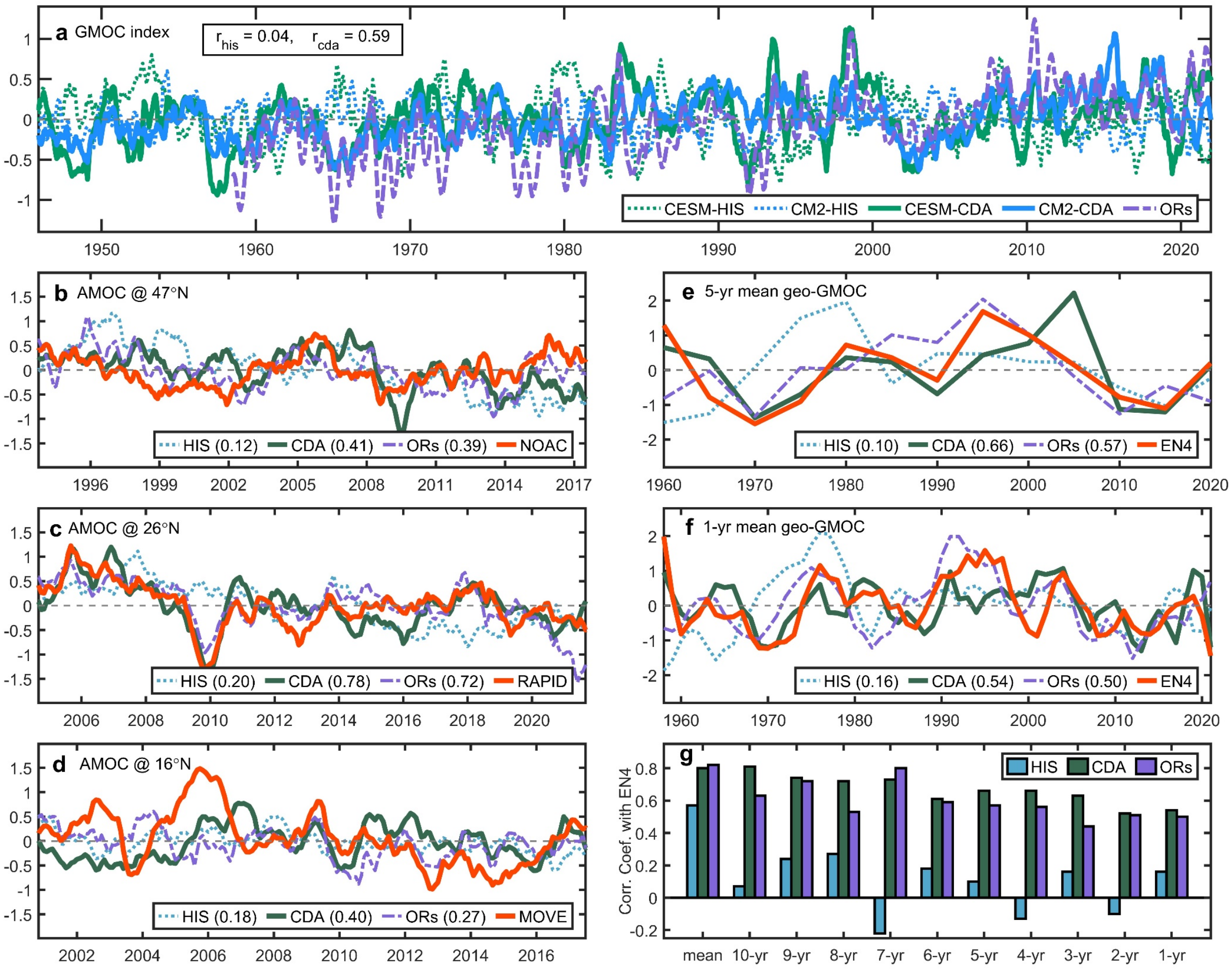

Supplementary Fig. 17 | a, Timeseries of 12-month running mean GMOC indices in two model historical simulations (CESM-HIS and CM2-HIS) and CDA estimates (CESM-CDA and CM2-CDA), as well as the mean of SODA3, ECCO4 and ORAS5 ocean reanalysis products (denoted as ORs). **b–d,** Timeseries of meridional mass transport anomalies at 47°, 26°, and 16° N sections in the Atlantic, respectively, for the mean of CESM-HIS and CM2-HIS (denoted as HIS), mean of CESM-CDA and CM2-CDA (denoted as CDA), ORs, as well as the observations of NOAC (**b**), RAPID (**c**), and MOVE (**d**) arrays. **e–f**, Timeseries of geostrophic GMOC indices with 5-year mean data (**e**), and yearly mean data (**f**), in HIS, CDA, ORs and the ocean objective analysis product EN4. **g**, Variation of anomaly correlation coefficients between the HIS, CDA, and ORs geostrophic GMOCs and the EN4 geostrophic GMOC as the timescale changes from low-frequency to high-frequency. The mean state correlation coefficient (denoted by 'mean') is for spatial pattern correlation of temporal mean structure of geostrophic GMOCs. See **Methods** for the definition of the GMOC index. All correlation coefficient statistics for CDA the 95% significance level. Generated by MATLAB R2025a.

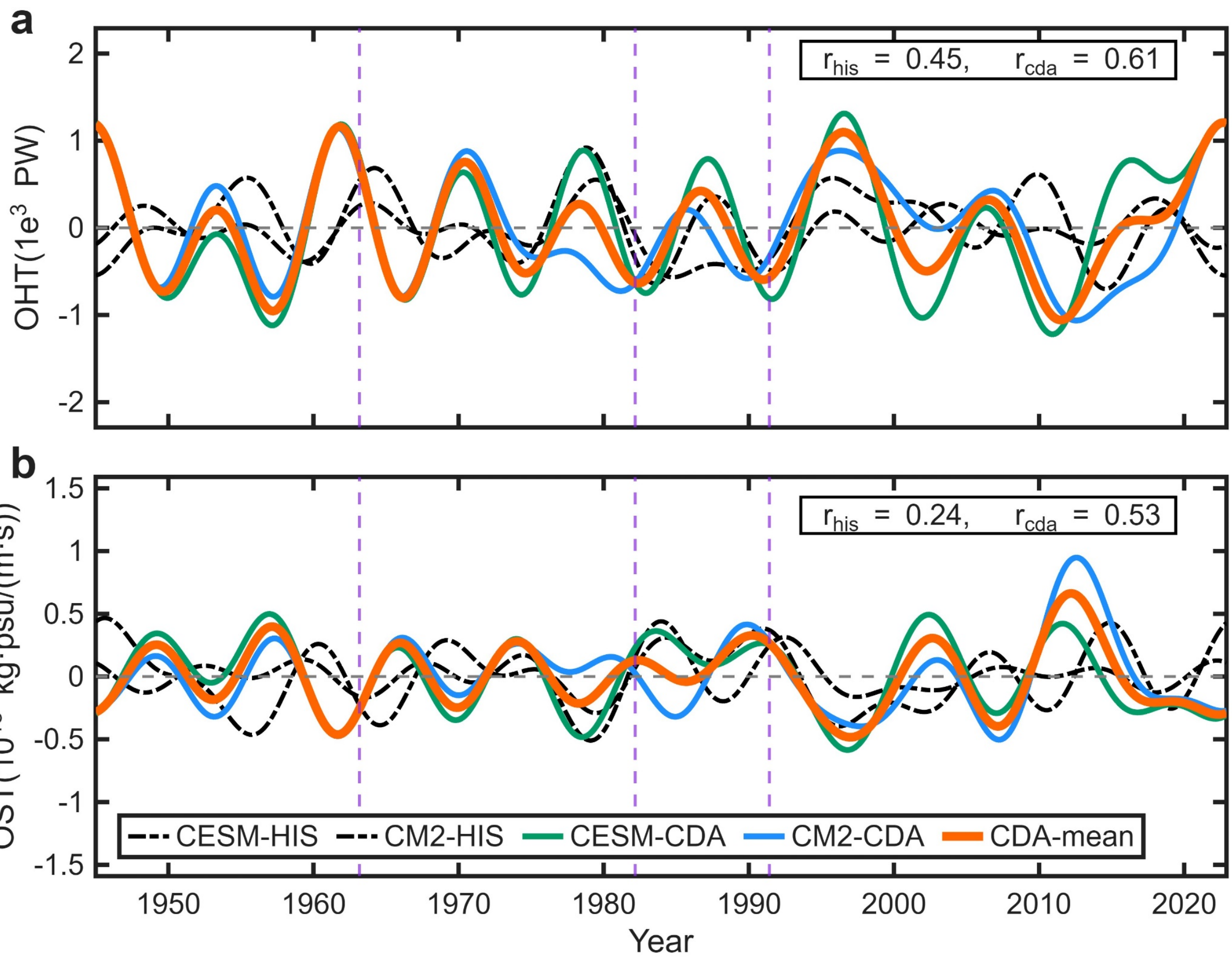

Supplementary Fig. 18 | Timeseries of (**a**) ocean meridional heat transport (OHT) above 1500 m (10^3 PW) and (**b**) ocean salinity transport (OST) below 1500 m (1×10^{18} kg·psu·m^{-1}·s^{-1}) in historical simulations CESM-HIS (dashed-black) and CM2-HIS (dotted-black), coupled data assimilation experiments CESM-CDA (solid-green) and CM2-CDA (solid-blue) as well as the CDA-mean (solid-orange) at the 26° N section. The correlation coefficients between the historical simulations and CDA results are indicated in the top-right corner of each panel. Generated by MATLAB R2025a.

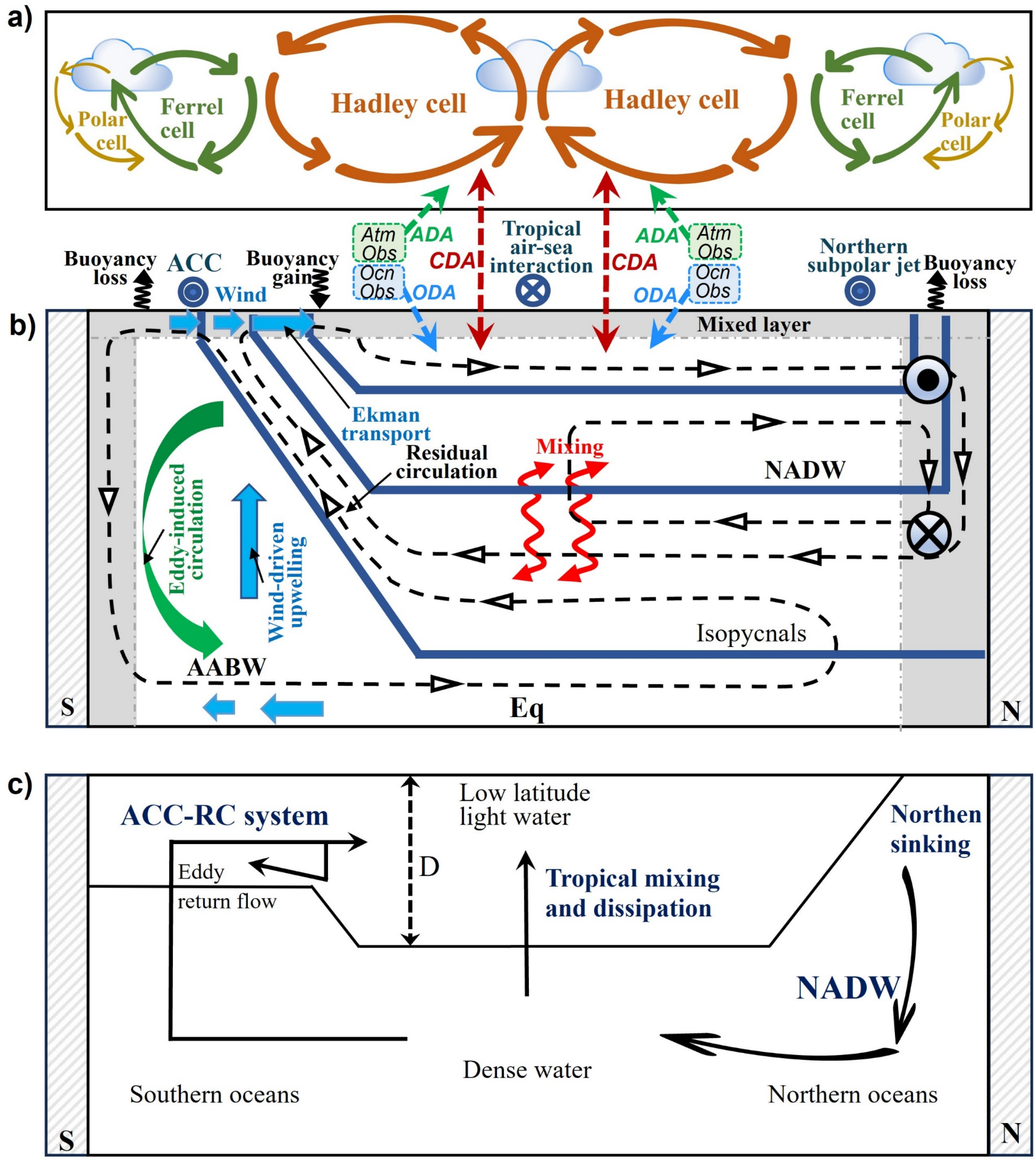

Supplementary Fig. 19 | Schematic illustration of GMOC's integration on cross-sphere climate signals in multiscale processes**. a**, Basic atmospheric mean meridional circulation (AMMC) consisting of Hadley, Ferrel and Polar cells. **b**, The configuration of processes at the air-sea interface include [36]: westerly jet at the Antarctic Circumpolar Current (ACC) region, buoyancy gain and loss induced by Ekman effects, northern subpolar jet, upward heat flux and buoyancy loss, and air–sea interactions associated with easterlies in the tropics. The configuration of ocean interior processes include: Northen Hemisphere (NH) sinking to form the North Atlantic Deep Water (NADW), diffusive mixing in tropical oceans, Southern Hemisphere (SH) wind-driven Ekman transport, eddy-induced circulation, and wind-driven upwelling and eddy-induced circulation associated with the Antarctic Bottom Water (AABW). **c**, The mechanism summarized from **b** for GMOC based on the three-term balance model, i.e., the residual between the difference of NH sinking and SH upwelling subtracting tropical upwelling drives the change in pycnocline depth anomaly [37]. All panels are plotted using Microsoft PowerPoint 2021.

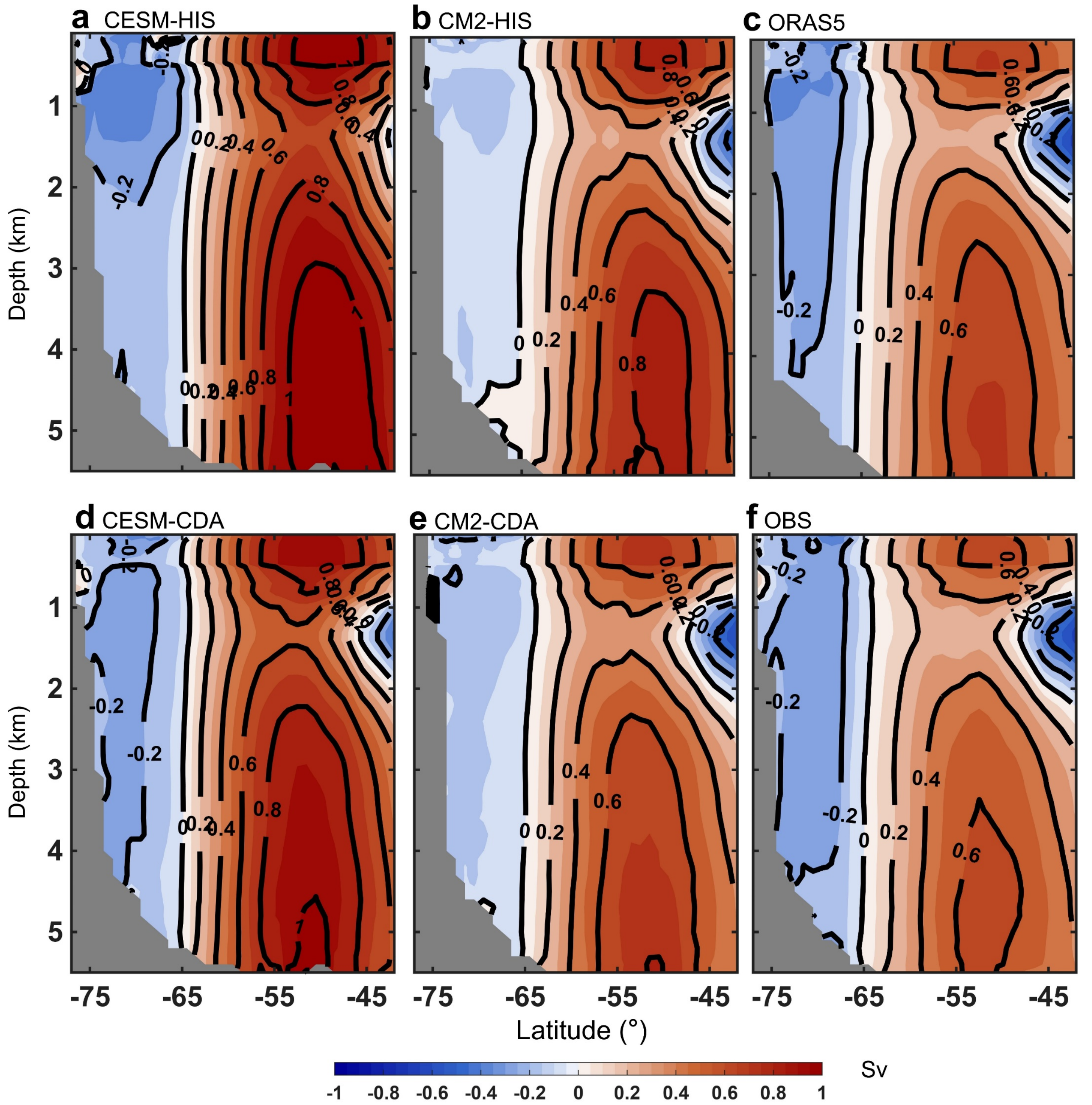

Supplementary Fig. 20 | a–f, Mean states of Antarctic Circumpolar Current residual circulation (ACC-RC) stream functions for **a**, CESM-HIS, **b**, CM2-HIS, **c**, ORAS5, **d**, CESM-CDA, **e**, CM2-CDA, and **f**, observational result (calculated from EN4 and ERA5). Generated by MATLAB R2025a.

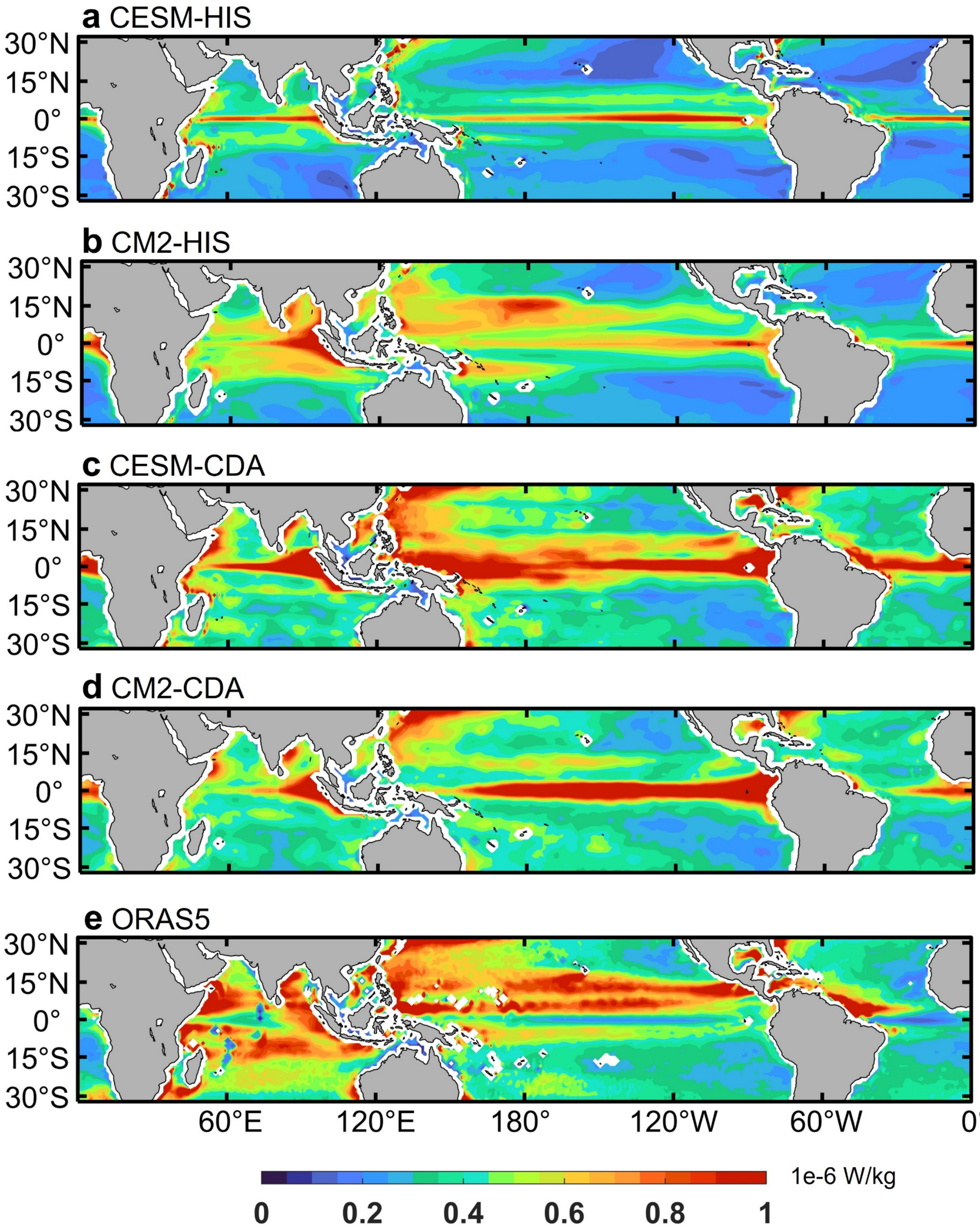

Supplementary Fig. 21 | a–e, Temporal mean distributions of tropical (30° S–30° N) kinetic energy dissipation rate [ε_m] (unit: 10^{-6} W·kg^{-1}) in CESM-HIS (a), CM2-HIS (b), CESM-CDA (c), CM2-CDA (d), and ORAS5 ocean reanalysis (e). Generated by MATLAB R2025a.

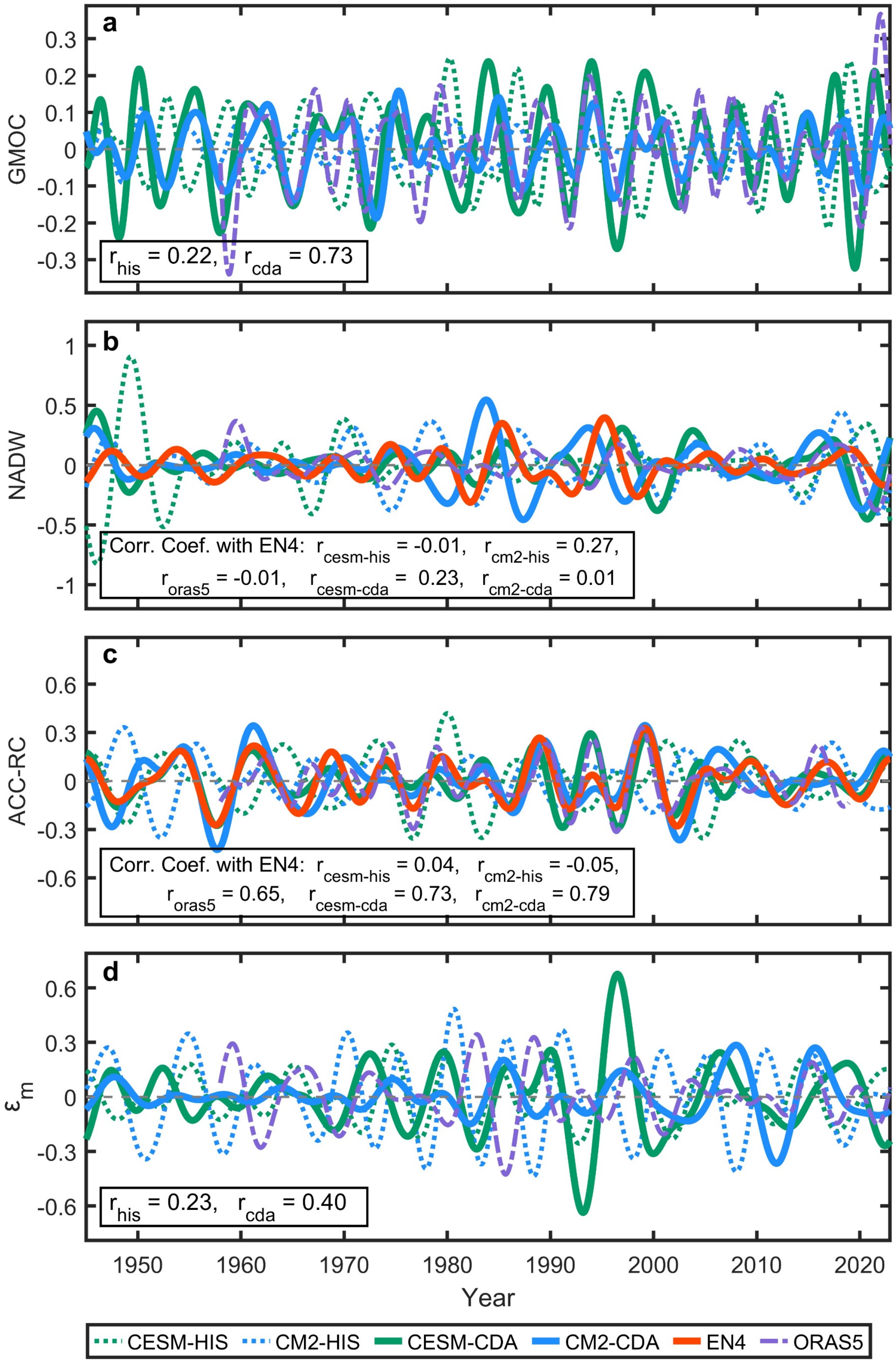

Supplementary Fig. 22 | Same as **Fig. 1i–l**, but for 2–5 years filtering of timeseries for GMOC (**a**), North Atlantic Deep Water (NADW) (**b**), Antarctic Circumpolar Current residual circulation (ACC-RC) (**c**) and tropical diffusive mixing (ε_m) indices (**d**). Generated by MATLAB R2025a.

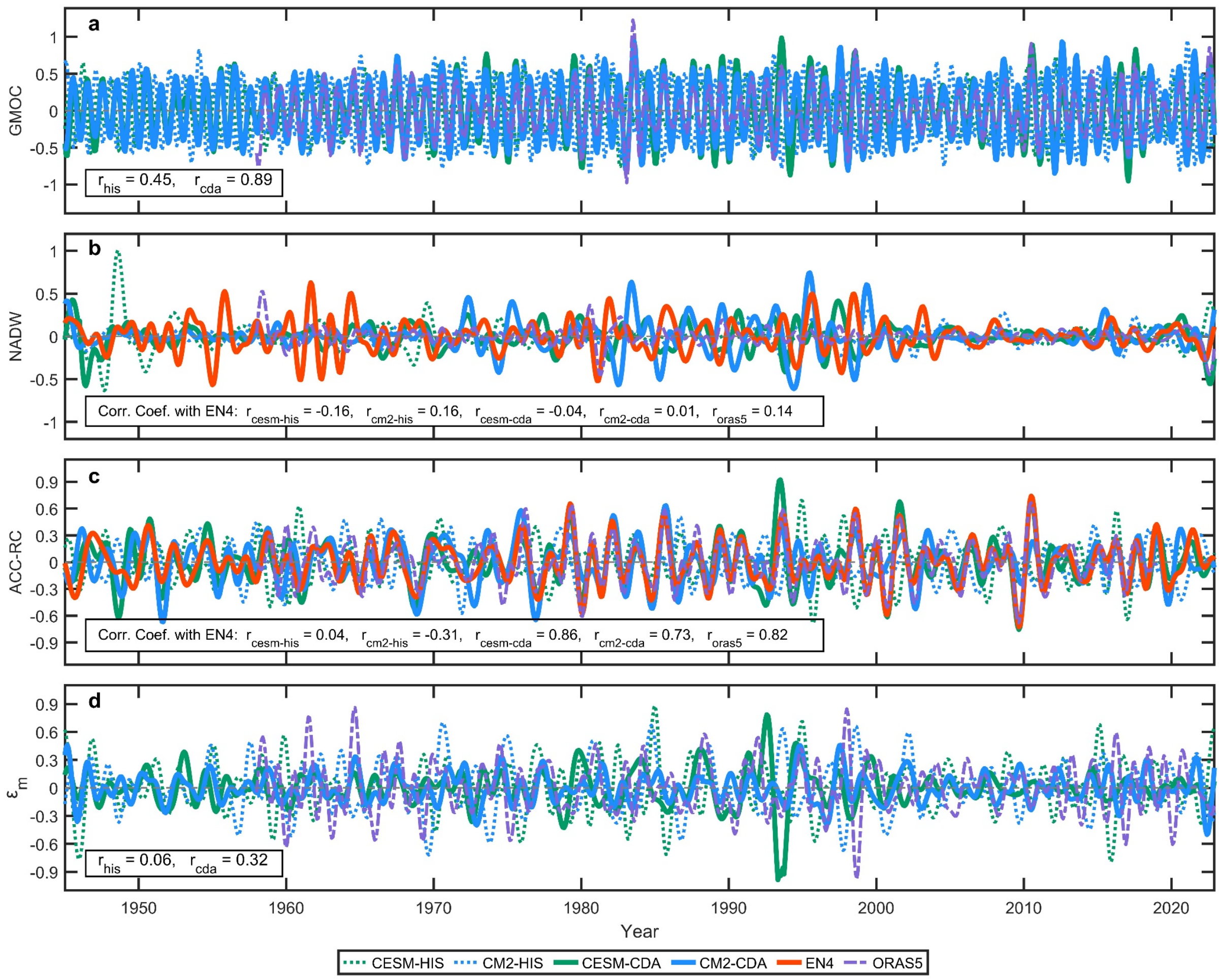

Supplementary Fig. 23 | Same as **Fig. 1i–l**, but for 6 months–2 years filtering of timeseries for GMOC (**a**), North Atlantic Deep Water (NADW) (**b**), Antarctic Circumpolar Current residual circulation (ACC-RC) (**c**) and tropical diffusive mixing (ε_m) indices (**d**). Generated by MATLAB R2025a.

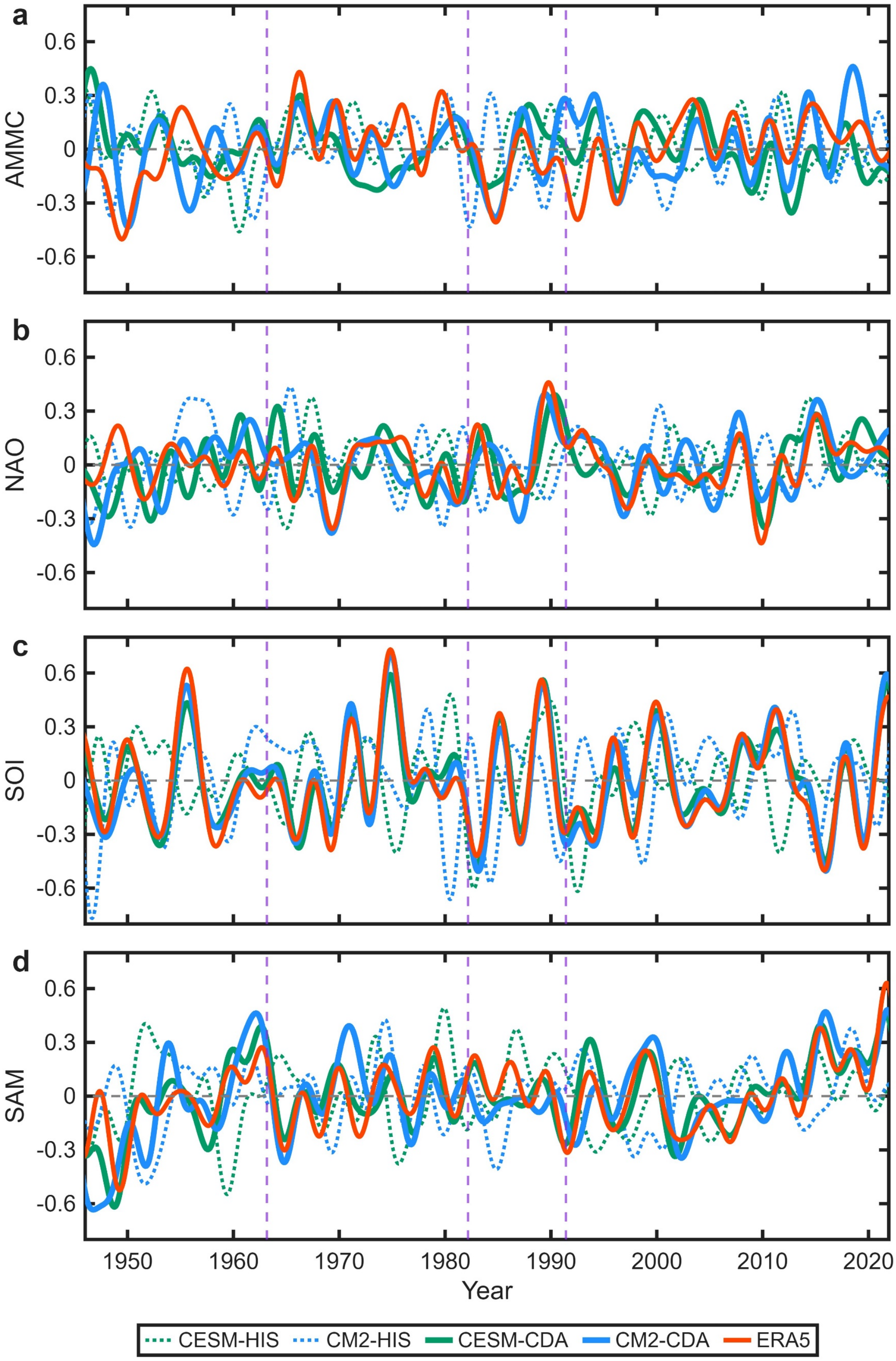

Supplementary Fig. 24 | a–d, The normalized 2–30-yr band-pass filtered timeseries of AMMC (**a**), North Atlantic oscillation (NAO) (**b**), Southern Oscillation Index (SOI) (**c**), and Southern Annular Mode (SAM) indices (**d**). Generated by MATLAB R2025a.

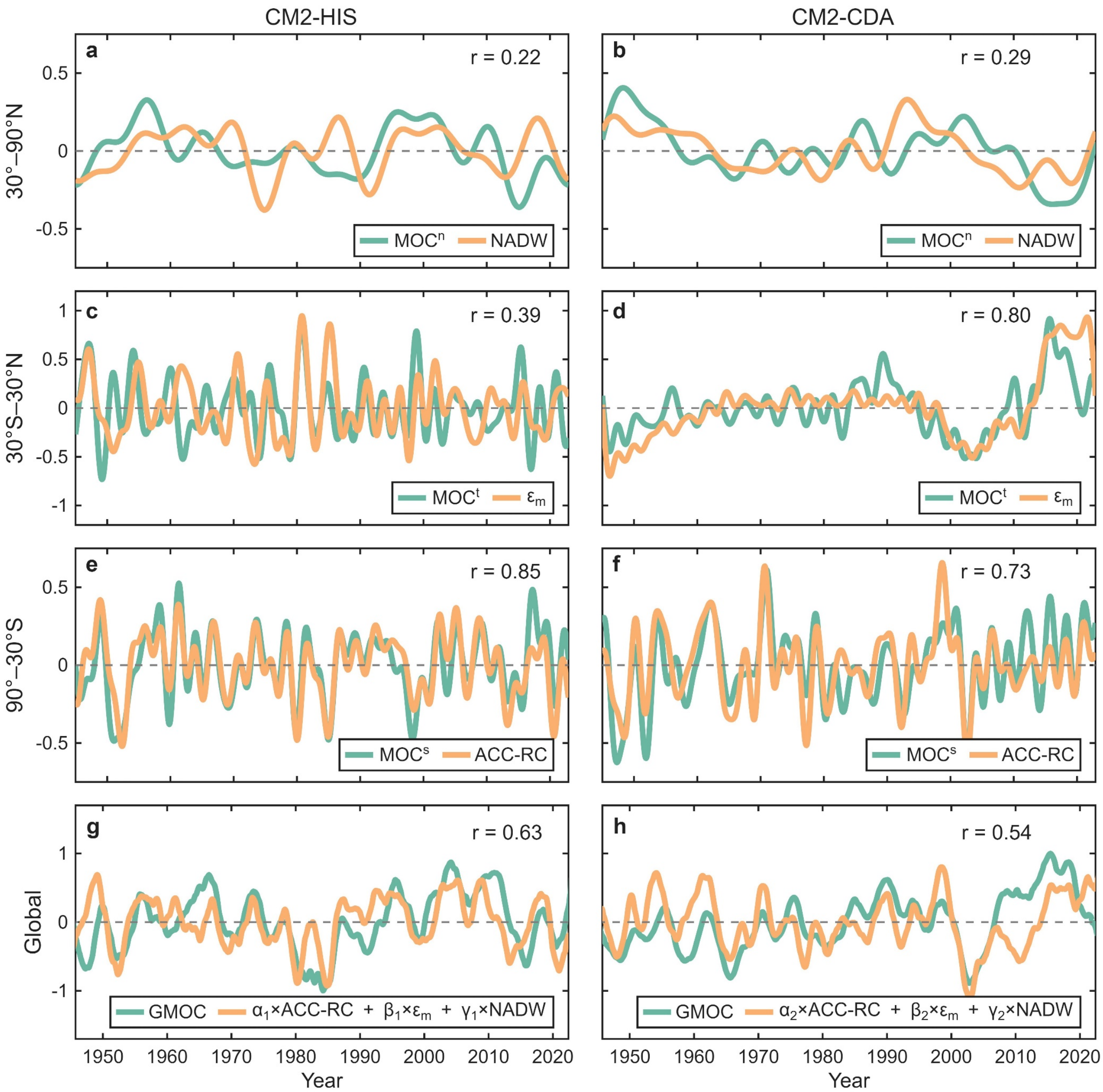

Supplementary Fig. 25 | Composited GMOC with its physical modes in CM2 model simulation and coupled reanalysis. a–b, The first principal component (PC1) derived from empirical orthogonal function (EOF) decomposition of GMOC over the area north of 30° N (denoted as MOC^n) and 5–30-yr filtered timeseries of normalized North Atlantic Deep Water (NADW) in CM2-HIS (**a**) and CM2-CDA (**b**). **c–d**, Same as **a–b**, but for PC1 of GMOC over the area of 30° S–30° N (denoted as MOC^t) and 6-month–20-yr filtered kinetic energy dissipation rate (ε_m). **e–f**, Same as **a–b**, but for PC1 of GMOC over the area south of 65° S (denoted as MOC^s) and the 1–20-yr filtered Antarctic Circumpolar Current residual circulation (ACC-RC). **g–h**, Timeseries of the 24-month running mean GMOC index and linear combination of ACC-RC, ε_m, and NADW with coefficients α, β, and γ, respectively. The parameters α, β, and γ are the linear regression coefficients between the GMOC indices and ACC-RC, ε_m, NADW indices, respectively, as $[\alpha_1, \beta_1, \gamma_1] = [0.39, -0.28, 0.04]$ and $[\alpha_2, \beta_2, \gamma_2] = [0.30, 0.39, 0.01]$. Generated by MATLAB R2025a.

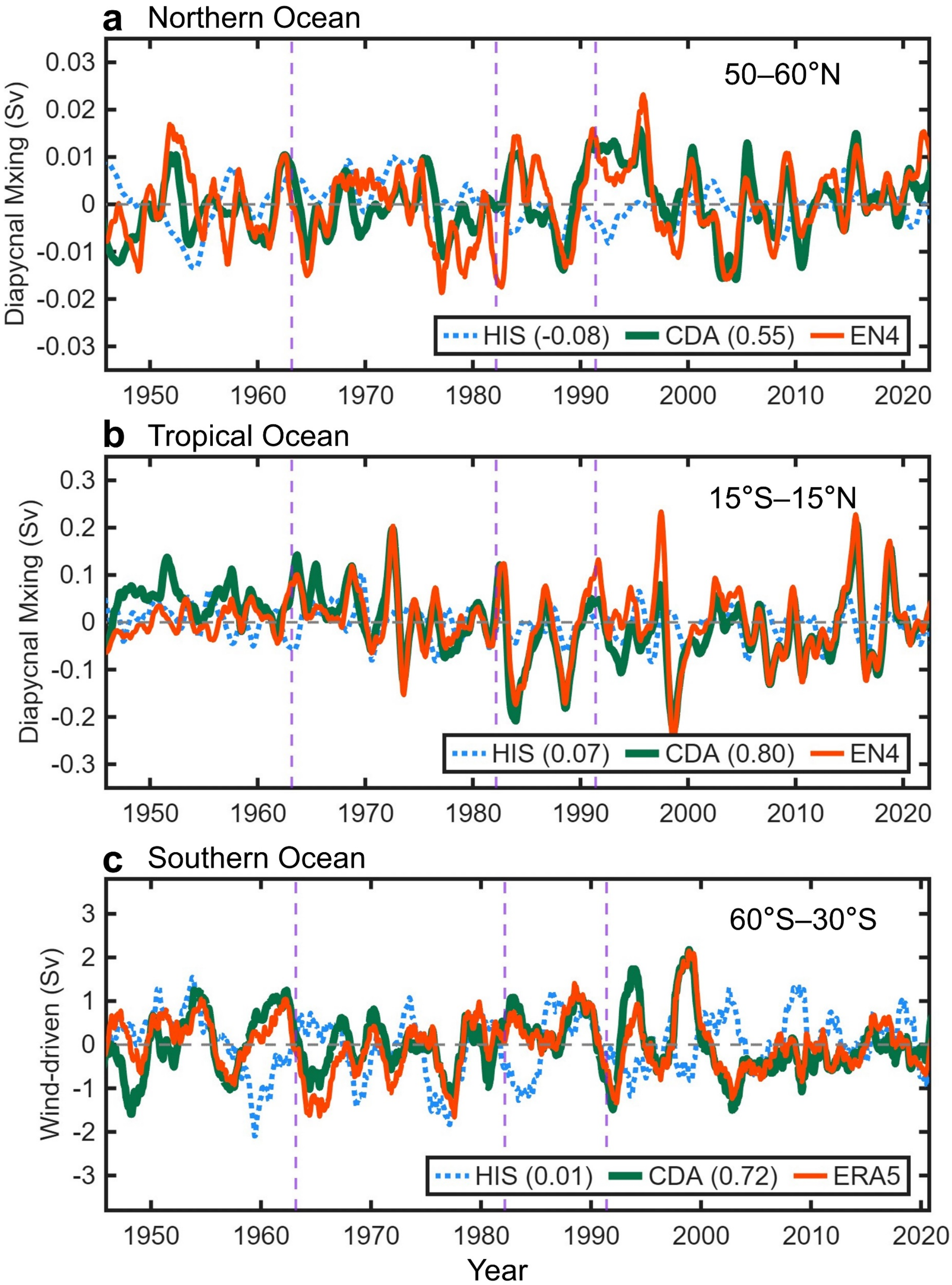

Supplementary Fig. 26 | Evaluation of key terms contributing to the CDA GMOC as diapycnal mixing and wind driven. **a**, Time series of Northern Ocean (50–60°N) diapycnal mixing-induced transport, from the historical simulation (HIS, blue dashed), CDA (green solid), and EN4 observations (orange solid). Correlation coefficients with EN4 are shown in parentheses for each dataset. **b,** Same as **a**, but for the Tropical Ocean (15°S–15°N). **c,** Time series of wind-driven Ekman transport (Sv) in the Southern Ocean (60°S–30°S), from HIS (blue dashed), CDA (green solid), and ERA5 reanalysis (orange solid). Correlation coefficients with ERA5 are shown in parentheses. Generated by MATLAB R2025a.

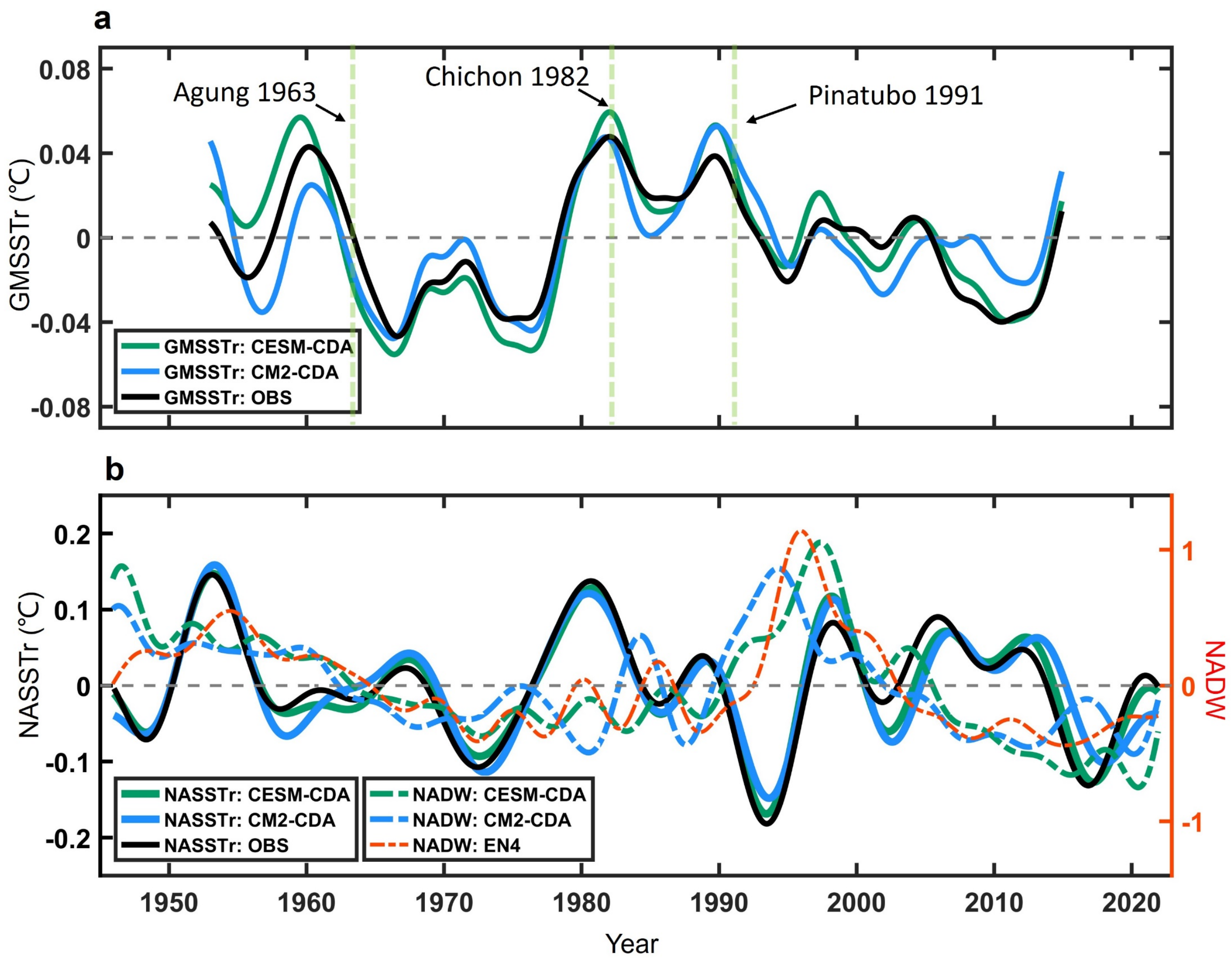

Supplementary Fig. 27 | a, Timeseries of GMSSTr for CDA estimates CESM-CDA (green-solid) and CM2-CDA (blue-solid) as well as observation (OBS, black-solid) (°C). The GMSSTr is obtained by removing the quadratic fit from the GASST (computed from 8-year low-passed SST). The GMSST is defined as area-weighted mean SST between 40°S and 60°N and anomalies are relative to the 1980–2010 mean [32]. **b**, Timeseries of 5–20-yr band-pass filtering NASSTr for observation (black-solid, °C) and 2–20-yr band-pass filtering normalized NADW for CESM-CDA (green-dash) and CM2-CDA (blue-dash). The NASSTr is obtained by removing the quadratic fit from the NASST, which is computed from 1-year low-passed SSTA over the area of 55°–35° W and 45°–65° N. Generated by MATLAB R2025a.

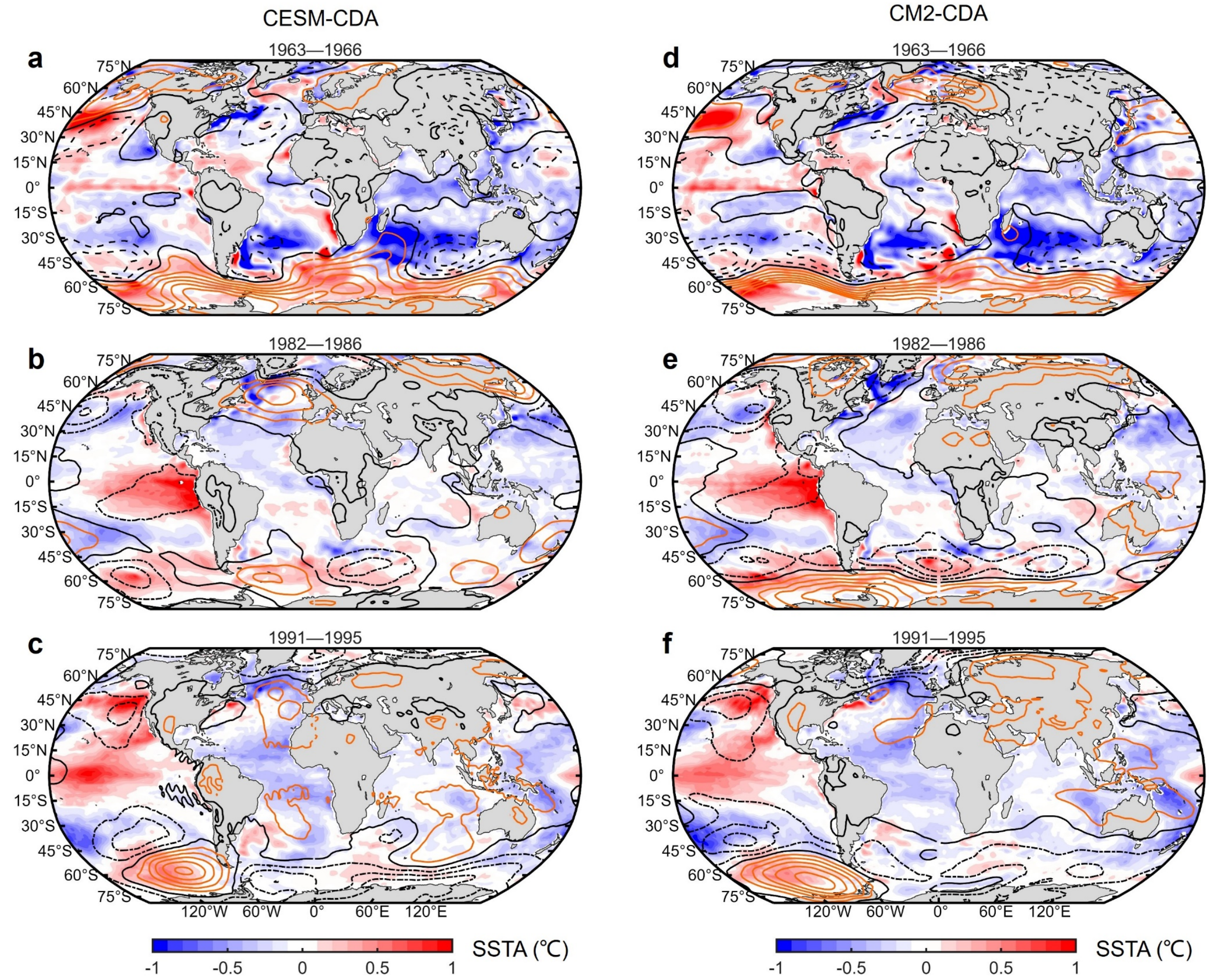

Supplementary Fig. 28 | a–b, Distribution of sea surface temperature (SST) anomaly (SSTA) during the period of 1963–1966 for CESM-CDA (**a**), and CM2-CDA **(b**). **c–d**, Same as **a–b**, but for period 1982–1985. **e–f**, Same as **a–b**, but for period 1991–1995. Generated by MATLAB R2025a.

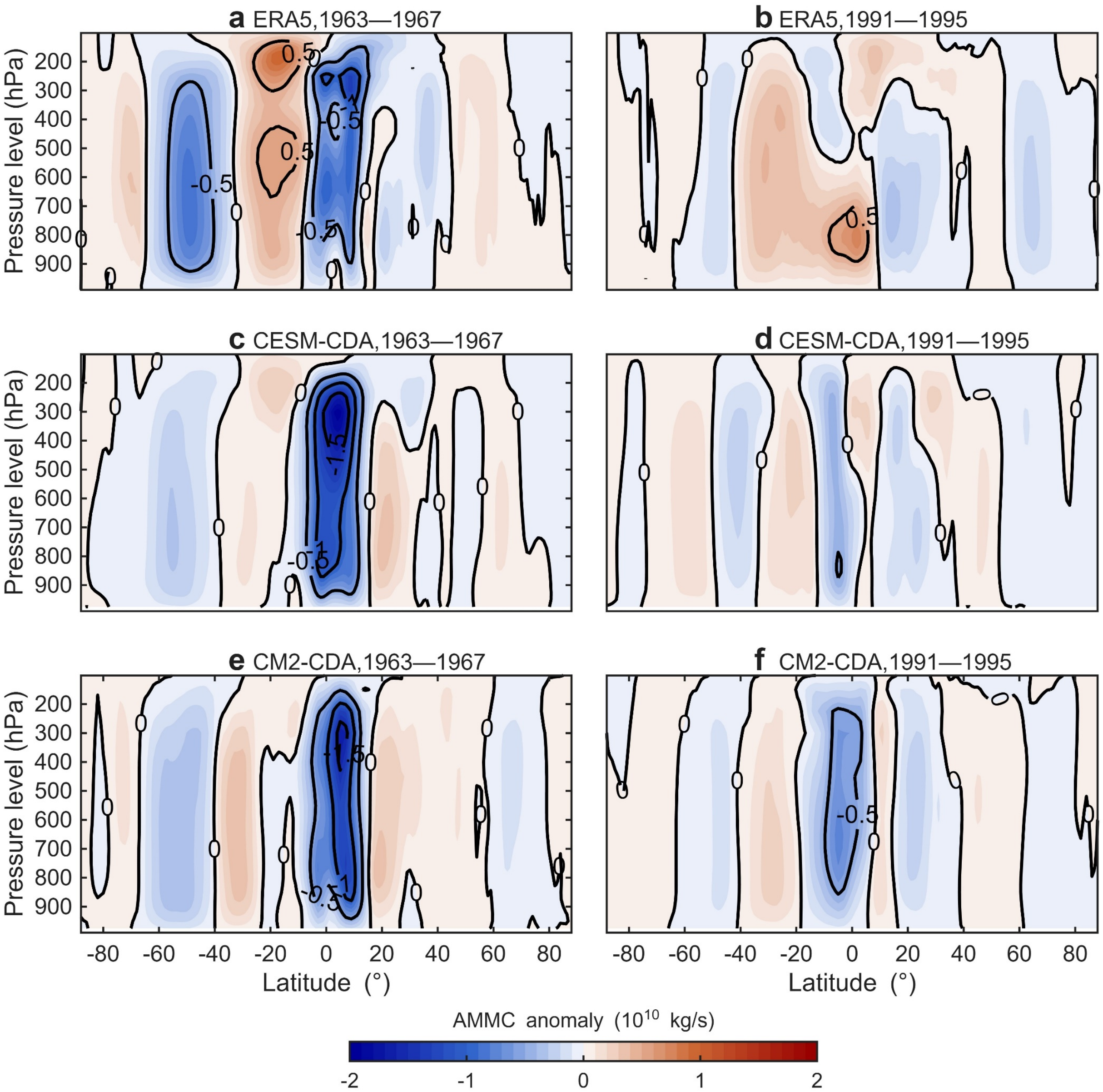

Supplementary Fig. 29 | Atmospheric meridional mean meridional circulation (AMMC) anomaly distributions during (left panels) 1963–1967 and (right panels) 1991–1995 for ERA5 (**a–b**), CESM-CDA (**c–d**), and CM2-CDA (**e–f**) (unit: 1×10^{10} kg·s^{-1}). Generated by MATLAB R2025a.

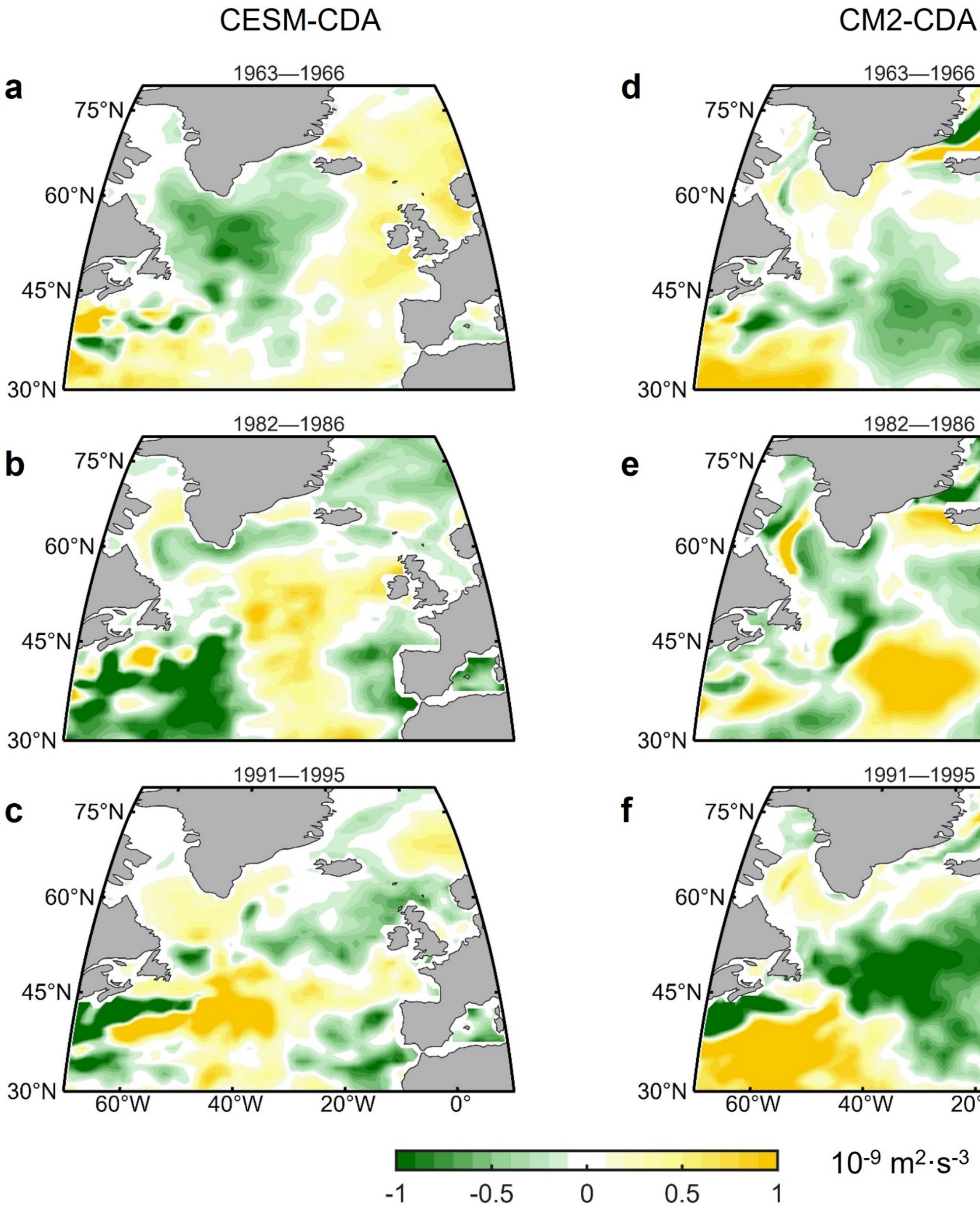

Supplementary Fig. 30 | a–f, Spatial distributions in the North Atlantic of 5-year (2-year) mean surface buoyancy flux difference (10^{-9} m^2/s^3) after and before the Agung (1963) (5-year mean) (a, d), Chichon (1982) (2-year mean) (b, e) and Pinatubo (1991) (5-year mean) (c, f) volcanic eruptions, for CESM-CDA (left) and CM2-CDA (right). Generated by MATLAB R2025a.

Supplementary Tables 1–3:

Supplementary Table 1. List of acronyms.

**Acronym**	**Full name**	**Description/Definition/Role in this study**
ACC	Antarctic Circumpolar Current	An ocean circulation system around the Antarctic
ACC-RC	Residual circulation of the Antarctic Circumpolar Current system	A circulation to reflect the balance between direct Ekman effects and counteracting influences of eddy-induced transport in ACC.
AM2.1	Atmosphere Model version 2.1	Atmospheric component model of CM2
AMMC	Atmospheric mean meridional circulation	The atmospheric vertical circulation in y-z plane
AMOC	Atlantic meridional overturning circulation	An important branch of ocean meridional overturning circulation in the Atlantic
Argo	Array for real-time geostrophic oceanography	An international program that deploys a global array of free-floating profiling floats to measure upper 2000 ocean temperature and salinity
CAM5	Community Atmosphere Model version 5	Atmospheric component of CESM
CDA	Coupled data assimilation	A data constraint approach using coupled models
CESM	Community Earth System Model developed at the National Center for Atmospheric Research	One of coupled models used in this study as assimilation model
CESM-CDA	CDA system/experiment using CESM	CDA system/experiment by applying multiscale CDA algorithm to CESM
CESM-CDA0	The formal version of CDA experiment using CESM	Experiment evaluated in Jiang et al. (2024)
CESM-HIS	Historical simulation experiment of CESM	CESM control run without data assimilation
CICE4	Community Ice Code version 4	An ice component model of CESM
CLM4	Community Land Model version 4	A land component model of CESM
CM2	The second generation of Coupled Climate Model developed at the Geophysical Fluid Dynamics Laboratory	One of coupled models used in this study as assimilation model
CM2-CDA	CDA system/experiment using CM2	CDA system/experiment by applying multiscale CDA algorithm to CM2
CM2-CDA0	The formal version of CDA experiment using CM2	Experiment evaluated in Jiang et al. (2024)
CM2-HIS	Historical simulation experiment of CM2	CM2 control run without data assimilation
ECCO4	Estimating the Circulation and Climate of the Ocean version 4 release 3	One of 3 ocean reanalysis products used in this study as references
EN4	Version 4 of global ocean objective analysis dataset of observations combining from two European Union projects: ENACT (Enhanced Ocean Data Assimilation and Climate Prediction; http://www.ecmwf.int/research/EU_projects/ENACT/index.html) and ENSEMBLES (http://ensembles-eu.metoffice.com/index.html).	The subsurface temperature and salinity observation dataset used in this study to evaluate CDA results
EOF	Empirical Orthogonal Function	An analysis method; Each EOF represents a spatial pattern.
ECMWF	European Centre for Medium-Range Weather Forecasts	An institution on numerical weather and climate prediction
ERA5	The fifth-generation ECMWF reanalysis starting from 1940	Atmospheric reanalysis products used for CDA's atmosphere "observation" and validation in this study

ERSSTv5	Extended Reconstructed SST version 5	SST observation product used to evaluate GMSSTr and NASSTr
GFDL	Geophysical Fluid Dynamics Laboratory	The institution of NOAA, who developed the CM2 model
GMOC	Global ocean meridional overturning circulation	The main theme in this study
MOC^n	The northern component of GMOC	The first Principal Component (PC1) of GMOC stream functions over the region north of 30°S
MOC^s	The southern component of GMOC	The PC1 of GMOC stream functions over the region south of 30°S
MOC^t	The tropical component of GMOC	The PC1 of GMOC stream functions over the region 30° S–30° N
GLORYS12	Global Ocean Physics Reanalysis	Ocean reanalysis product as reference in this study
GMSST	Global mean SST	An index of observed global mean SST
GMSSTr	Global mean SST residual	An index used to show the responses of observed SST on major volcanic eruptions
HadISST	Hadley Centre Sea Ice and Sea Surface Temperature Dataset	SST observation product used for CDA in this study
HF_{O2A}	Oceanic heat flux to the atmosphere	Feedback of the ocean to the atmosphere
IPCC	Intergovernmental Panel on Climate Change	An international organization created in response to the growing concern about human-induced climate change
IPMOC	Indo-Pacific meridional overturning circulation	The stream function of ocean meridional overturning circulation for the Indian Ocean and Pacific Ocean
LM2.1	Land Model version 2.1	A land component model of CM2
MOM4	Modular Ocean Model version 4	An ocean component model of CM2
MOVE	Meridional Overturning Variability Experiment	Volume transport data mooring observations at 16° N in the western Atlantic
NADW	North Atlantic Deep Water	The NADW index is defined as the average thickness between two isopycnals of $\sigma_{1.5}$ over the North Atlantic, see **Materials and Methods b3**.
NAO	North Atlantic Oscillation	A timeseries of the leading EOF of SLP anomalies over the Atlantic sector, 20°–80° N, 90° W–40° E
NAOC	The North Atlantic Changes	Volume transport data mooring observations at 47° N in the western Atlantic
NASSTr	North Atlantic SST residual	An index used to show the responses of observed SST over Northern Atlantic on major volcanic eruptions
NCAR	National Center for Atmospheric Research	The institution develops CESM model
NH	Northern Hemisphere	One half of the Earth in north of the equator
NOAA	National Oceanic and Atmospheric Administration	An agent of US federal government
NOCS	National Oceanography Centre Southampton	An observational sea surface heat flux dataset
OISST	Optimum Interpolation Sea Surface Temperature Dataset	SST observation product used for CDA in this study
ORAS5	Ocean Reanalysis System 5	Ocean reanalysis product as reference in this study
OSB-CDR	SeaFlux Ocean Surface Bundle Climate Data Record	An observational sea surface heat flux dataset
PC	Principal component, referred as PC1 (first), PC2 (second) etc.	The temporal evolution associated with each EOF

POP2	Parallel Ocean Program version 2	An ocean model component of CESM
Ps	Surface pressure	A basic variable of atmosphere state
RAPID	Rapid Climate Change: Meridional Overturning Circulation and Heatflux Array	A collaborative research project for measuring transport at 26.5°N in the Atlantic
RCP45	Representative Concentration Pathway 4.5	A medium-emission scenario, in which the radiative forcing level stabilizes at around 4.5 W/m² by 2100.
RMSE	Root mean square error	A measure of quantifying the differences between the estimated values and actual observed values
SAM	Southern Annular Mode	The principal mode of variability in the SH extra-tropics, describing ~30% of SH variability
SH	Southern Hemisphere	One half of the Earth in south of the equator
SLP	Sea level pressure	A basic variable of atmosphere state
SODA3	Simple Ocean Data Assimilation version 3.4.2	Ocean reanalysis product as reference in this study
SOI	Southern Oscillation Index	An index used to characterize the large scale SLP patterns in the tropical Pacific
SST	Sea Surface Temperature	A basic variable of ocean state
STD	Standard deviation	The dispersion of data relative to the mean
W_{A2O}	Atmospheric work exerted on the ocean	An important variable in atmosphere-ocean coupling process
WBCs	West boundary currents	An important ocean currents system
WOA	World Ocean Atlas	Ocean temperature and salinity observational climatology used for deep ocean restoring in CDA and validation of CDA results
ε_m	Kinetic energy dissipation rate	The diagnosed tropical diffusive mixing effects
$\sigma_{1.5}$	The potential density referenced to a depth of 1500 m	The isopycnals used to define NADW

Supplementary Table 2. Description of reanalysis experiments.

**Experiment**	**Model information**	**Data constraint**	**Period**
**CESM-HIS**	**NCAR-CESM1.3**<br>Atmospheric component: CAM5<br>1° × 1°, 26 layers<br>Oceanic component: POP2<br>1° × 1°, 60 layers	1945–2006: historical radiative forcing<br>2007–present: RCP45 scenario	1945–present
**CESM-CDA**		Grided surface pressure (Ps) from ERA5, grided SST from HadISST and OISST, in-situ temperature and salinity profiles, restoring to World Ocean Atlas 2018 (WOA18) in deep ocean<br>Radiative forcing is the same as CESM-HIS.	
**CM2-HIS**	**GFDL-CM2.1**<br>Atmospheric component: AM2.1<br>2° × 2.5°, 24 layers<br>Oceanic component: MOM4<br>1° × 1°, 50 layers	Same as CESM-HIS	
**CM2-CDA**		Same as CESM-CDA	

Supplementary Table 3. Information of reanalysis products.

Reanalysis	Ocean model	Resolution	Forcing	Period
**SODA3.4.2**	Modular Ocean Model	0.5° × 0.5° 50 layers	ERA-interim	1980–2019
**ECCOv4r3**	Massachusetts Institute of Technology General Circulation Model	0.5° × 0.5° 50 layers		1992–2015
**ORAS5**	Nucleus for European Modelling of the Ocean	0.25° × 0.25° 75 layers		1958–present
**GLORYS12**	Nucleus for European Modelling of the Ocean	0.125°× 0.125° 50 layers		1993–2016
