# Supplementary material for: The coupled reanalysis global meridional overturning circulation imprinted by historic volcanic eruptions
Source: Nat Commun. 2026 Jul 16;17:8703. doi: 10.1038/s41467-026-75651-z (PMC13490444; doi:10.1038/s41467-026-75651-z)
Supplement: Supplementary file 2 [file 41467_2026_75651_MOESM2_ESM.pdf]

nature portfolio

# Peer Review File

# The Coupled Reanalysis Global Meridional Overturning Circulation Imprinted by Historic Volcanic Eruptions

Corresponding Author: Dr Shaoqing Zhang

**This file contains all reviewer reports in order by version, followed by all author rebuttals in order by version.**

**Attachments originally included by the reviewers as part of their assessment can be found at the end of this file**

Parts of this Peer Review File have been redacted as indicated to remove third-party material.

Version 0:

Reviewer comments:

Reviewer #1

(Remarks to the Author)
See the attachment.

Reviewer #2

(Remarks to the Author)
Review of “Organization of historical oceanic overturnings on cross-sphere climate signals”

The manuscript provides an interesting assessment of GMOC from coupled reanalyses that outperform in many aspects more conventional (uncoupled) reanalyses, stressing the benefits of a state-of-the-science CDA approach in representing key large-scale climate processes. It is suitable for publication, but several aspects need to be improved, which is why I recommend a major revision of the manuscript. Please check the points below, in order as they appear (rather than major and minor; however, points 11, 12, and 13 are those “major”).

1. The title itself is quite cryptic and not easy to understand, so I recommend choosing a clearer and more appealing title (for instance, mentioning the convergence of the two reanalyses, the benefits of the methodology in representing the global overturning circulation, or similar).

2. Related to point 1), the reference to “cross-sphere” in the title and elsewhere is not self-evident. It could refer to multiple Earth system components or timescales used within the DA (I presume it is the second option, in any case is not obvious at all). I suggest replacing with cross-component and/or multi-scales as needed, which are more standard definitions.

3. L32: “two” and not “multiple”

4. L34: The sentence is not clear. “printed in variability of the rebuilt GMOC” is hard to understand and needs rephrasing.

5. L39-41 I suggest expanding the discussion on the importance of GMOC on the global climate, as only a couple of lines is not enough to set up the motivation of the work.

6. Additionally, still in the initial methodological discussion, inverse methods (box models, etc.) can be used to infer GMOC, together with other indirect methods, and need to be mentioned and cited.

7. L44 “multi-spheric” again unclear

8. L52 “especially in the deep ocean”, as bias does not only occur at depth

9. Many references (eg, 24, etc.) refer to AMOC and not GMOC, but the authors use them for GMOC. I suggest carefully checking in order not to consider for GMOC works that focus on AMOC.

10 L64 Why is “observations” quoted?

11. The results about the convergence of the two reanalyses in terms of GMOC are interesting. However, the two historical simulations from CESM and CM2 appear quite close as well in the way they reproduce the GMOC. It would be important to understand whether a completely different representation of the GMOC from a data-unaware coupled model would converge, with CDA, to the same reanalyzed GMOC. I understand this is difficult to prove in practice, but if the authors have any comments or speculation, it would be interesting.

12. The choice of ocean reanalyses used in Fig 1 seems arbitrary; either there is a very strong justification for including ORA and SODA, or I suggest to include (at least here) also other reanalyses, such as GLORYS12, ECCO, CGLORS, MOVE, etc., which in turn would make the assessment more complete. There was also a coupled reanalysis, CERA. produced by ECMWF that would offer another comparison dataset closer to the formulation of the CDA here (although CERA was probably not built for low-frequency variability studies): I think it could be at least mentioned and shown, if data are available. The manuscript will become of broader interest if these datasets are compared.

13. The other major point is about the reasons for the convergence. The authors are confident about the fact that the CDA approach leads to convergent and improved representation of GMOC and underlying processes. However, while they show many diagnostics that suggest so, this is not fully conclusive. For instance, when compared to the ocean reanalysis like ORAS5, the impact could be due to the i) use of coupled model instead of ocean-only; ii) use of coupled data assimilation; iii) use of surface pressure observations in the coupled DA scheme (are these crucial or not?); iv) use of multiple scales for the error covariances. While disentangling the merits of each enhancement compared to standard ocean reanalyses might be difficult, it will be important if the authors can discuss the relative merits of these CDA ingredients, as they certainly performed many preliminary experiments to reach the final configuration.

14. The results’ section is quite convincing, simple, and clear. I found, however, the discussion related to Figure 4 slightly disconnected, or perhaps to move at the beginning (e.g., as a proof of the reliability of the two reanalyses). Alternatively, the authors can improve the initial part of the section to make a clearer link and motivation with the previous analyses.

15. Methods: The bias adjustment is a quite standard and old method (strong relaxation to climatology, even with a 30d strength at the bottom), so I would not emphasize its sophistication.

16. L435: What do you mean by pressure data from ERA5? Are you assimilating gridded data from the reanalyses or raw observations?

17. L486: Is the 0.1 mm/s removal due to the use of the bottom as no motion level instead of the usual 1000 m- 2000 m of depth?

Version 1:

Reviewer comments:

Reviewer #1

(Remarks to the Author)

Reviewer #2

(Remarks to the Author)
The authors have revised the manuscript in a satisfactory way, addressing all my previous concerns, and I am therefore happy with the revision.
There is only one remaining point in my opinion (I should have mentioned it earlier, though): one important claim of the authors is the improvement borne by CDA, which is verified, for instance, in the turbulent fluxes. However, these are verified against ERA5. I think that while ERA5 is a good reference for the wind work, pressure, and all other parameters investigated, I doubt this can be a good reference for air-sea turbulent fluxes. I then suggest reporting any literature speculating on the accuracy of ERA5 turbulent fluxes and mentioning its limitations (here, what is important is the increase in consistency), and ideally, comparing with other independent datasets not based on models.
See for instance

https://journals.ametsoc.org/view/journals/atot/41/12/JTECH-D-24-0028.1.xml
https://www.sciencedirect.com/science/article/pii/S0012825223003513

Version 2:

Reviewer comments:

Reviewer #1

(Remarks to the Author)
The authors have fully responded to my comments and the manuscript of this version has been significantly improved. My suggestion is acception.

Reviewer #2

(Remarks to the Author)
The authors have satisfactorily addressed all my previous comments, and I am happy to recommend publication of the manuscript.

**Open Access** This Peer Review File is licensed under a Creative Commons Attribution 4.0 International License, which permits use, sharing, adaptation, distribution and reproduction in any medium or format, as long as you give appropriate credit to the original author(s) and the source, provide a link to the Creative Commons license, and indicate if changes were made.
In cases where reviewers are anonymous, credit should be given to 'Anonymous Referee' and the source.
The images or other third party material in this Peer Review File are included in the article's Creative Commons license, unless indicated otherwise in a credit line to the material. If material is not included in the article's Creative Commons license and your intended use is not permitted by statutory regulation or exceeds the permitted use, you will need to obtain permission directly from the copyright holder.
To view a copy of this license, visit https://creativecommons.org/licenses/by/4.0/

Reviewer #1:

Recommendation: Rejection

The authors present two 80-year coupled reanalyses (CESM-CDA and CM2-CDA) produced with a multiscale coupled-data-assimilation (CDA) scheme that jointly ingests atmospheric surface pressure, SST and subsurface temperature/salinity (1945-present). They claim the new reconstructions (i) converge on a common global meridional overturning circulation (GMOC) mean state, (ii) reproduce multiscale GMOC variability—including fingerprints of major volcanic eruptions—and (iii) will underpin "AI coupled big models." The work addresses the pressing need for dynamically consistent long-term Earth-system reanalyses, and the technical effort behind two independent CDA systems is impressive.

The CDA effort is valuable, but the manuscript in its present form suffers from the following fundamental weaknesses: the title is misleading, figures fall short of publication quality, CDA fails to improve the key diagnostic, the runs are not spun up for AMOC equilibrium, and no new scientific insight is provided, the manuscript should be rejected.

RE: All authors appreciate this reviewer's encouragement and constructive comments greatly. All concerns of title, figures, CDA diagnostics as well as new scientific insights etc. have been fully addressed in the revision We have changed the title, re-plotted all figures, added more CDA diagnostics and analyses and discussions on new scientific insights in the revision. What follows gives point-by-point responses for this reviewer's comments.

Major Comments:

1. Title–content mismatch. The manuscript's title—"Organization of Historical Oceanic Overturnings on Cross-Sphere Climate Signals"—does not reflect what is actually delivered. The paper is essentially a performance assessment of coupled-model hindcasts with and without coupled data assimilation (CDA). Terms such as "organization," "cross-sphere," and "climate signals" are never defined or demonstrated in the text, leaving the reader confused about the study's scope and novelty.

RE: Very good comment! We change title as "**The Coupled Reanalysis Global Meridional Overturning Circulation Imprinted by Historic Volcanic Eruptions**."

2. Poor figure quality. Many figures are pixelated, employ non-uniform "rainbow" colour scales, and use fonts below 6 pt, making key details illegible. Several panels lack units and consistent axis limits. The visual presentation is well below the standards expected for Nature Communications.

RE: Thanks for pointing out this deficit. We re-designed and re-plotted all Figures.

3. CDA does not outperform the historical run for overturning circulation. In Figure 1 the global/Atlantic meridional overturning circulation produced by the CDA integrations does not outperform the corresponding historical (HIS) simulations; in some depth ranges the skill is even worse. This directly contradicts the manuscript's central claim that CDA "substantially improves" overturning realism.

RE: Thanks for this good point which brought us more thoughts on our results! We have added more analyses and discussions on the similar feature of GMOC mean states in free model simulations, which is different from the similar patterns and variability of GMOC in the coupled reanalyses. Summarily speaking, due to common deficits on dynamics (coarsely-discretized fluid and quite smooth topography etc.) and physics (parameterized subgrid processes, for instance), the mean state of GMOCs in free model simulations have similar distorted features. In the revision, we place our primary emphasis on investigating multidecadal variability of GMOC and the impact of large volcanic eruptions on its dynamics. We change figure presentation and discuss this in the revision. Please see more detailed analyses and discussions for the new **Fig. 1** on **L82-87, L101-129** on the revised main context as well as specific discussions for the similar feature of GMOC mean states in free model simulations on **L131-147 of SI**.

4. Insufficient integration length for AMOC adjustment. The coupled integrations span approximately 72 years (1950–2022). For the AMOC this is far shorter than the adjustment time-scale required for the deep limb to reach quasi-equilibrium. Consequently, any diagnosis of heat–salt overturning variability is premature; only upper-ocean thermodynamic metrics can be considered marginally adequate.

RE: Thanks for the reviewer's concern about the spinup time of model-data integration, which really brought us more analyses and understanding on the equilibrium issue of CDA. We add more justification and discussions on data assimilation's spinup time to support and clarify our analyses. Summarily, due to direct data-constraints, the spinning up of ocean data assimilation is much quick than free model integration. Also, the climatological restoration for deep ocean in this study is crucial for coherence spinup of upper and deep oceans to rebuild the ocean's stratification. More detailed analyses and discussions are added in the revision (please see **L60-71 in SI** of the revision). We also add more diagnosis and analysis for heat and salt transport, which is presented in the new **Supplementary Fig. 15** and **L335-338 in SI**.

5. No genuine scientific discovery. The manuscript is essentially a model-to-model comparison exercise. Beyond reporting performance metrics, it offers no new physical insight into ocean

dynamics, climate interactions or predictability. Without a substantive scientific question and discovery, the work does not meet the novelty threshold for this journal.

RE: Thanks for reviewer's advice on this point. We substantially rewrote the manuscript which clearly addresses the scientific question and discovery as the new title and abstract state.

6. Bias-correction strategy may undermine "deep-ocean realism". A strong depth-dependent relaxation to World Ocean Atlas climatology is imposed below 1000 m (Methods, lines 423-431). This nudging dominates the deep stratification that is subsequently highlighted as a CDA "achievement" (Fig. 1g-n). The authors should clarify how much of the apparent improvement arises from the bias-relaxation rather than true multiscale CDA; include sensitivity tests without deep nudging.

RE: This point brought us more thinking on our results. In the revision, we state that using climatology data to restoring deep ocean is a part of multiscale oceanic data-constraints (please see **L67-71 of SI**). We add more discussions on the adverse impact of deep ocean bias on ocean stratification and the importance of controlling deep ocean bias to sustain the ocean variability reconstruction in the revision (please see **L237-251 of SI**).

7. Causal attribution for GMOC variability is largely descriptive. The manuscript relies on correlations among synthetic indices (GMOC, NADW thickness, ACC-RC, $\varepsilon m$) but lacks closed budget analyses. No meridional heat- or salt-transport diagnostics, streamwise potential-vorticity budgets, or overturning decompositions (isopycnal vs. diapycnal) are presented. The authors should supply full overturning budgets (wind-driven Ekman, eddy, diapycnal mixing, surface buoyancy forcing) for at least two key decades; quantify the contribution of each term to GMOC tendencies.

RE: Very excellent suggestions and advices on diagnostic analyses about GMOC! We add more diagnostic analyses advised by the reviewer in the revision and we learned a lot from these diagnostic studies. Using the CESM as a representative framework, we analyzed the surface buoyancy flux, diapycnal mixing, and eddy-induced meridional transport in the revision. Please see new **Fig. 4** and corresponding analyses on **L193-218 and L344-357**.

8. Overstatement of "AI big-model" relevance. The authors claim that the reanalyses will "give birth to Artificial Intelligence coupled big models" (abstract, lines 34-36) are marketing language.

RE: We agree! The statement is removed in the revision.

Reviewer #2:

The manuscript provides an interesting assessment of GMOC from coupled reanalyses that outperform in many aspects more conventional (uncoupled) reanalyses, stressing the benefits of a state-of-the-science CDA approach in representing key large-scale climate processes. It is suitable for publication, but several aspects need to be improved, which is why I recommend a major revision of the manuscript. Please check the points below, in order as they appear (rather than major and minor; however, points 11, 12, and 13 are those "major").

RE: Thanks a lot for this reviewer's encouragement! We have fully addressed each comment in the revision, especially taking more cautious and deep examination on 11, 12 and 13. Please see the following p2p responses.

**Point-by-point responses for Reviewer 2's comments:**

1. The title itself is quite cryptic and not easy to understand, so I recommend choosing a clearer and more appealing title (for instance, mentioning the convergence of the two reanalyses, the benefits of the methodology in representing the global overturning circulation, or similar).

RE: Thanks for this concern. We take the reviewer's suggestion. Combined with the reviewers' suggestions to reflect the scientific question for which our study is seeking the answer, we change the title as "**The Coupled Reanalysis Global Meridional Overturning Circulation Imprinted by Historic Volcanic Eruptions**."

2. Related to point 1), the reference to "cross-sphere" in the title and elsewhere is not self-evident. It could refer to multiple Earth system components or timescales used within the DA (I presume it is the second option, in any case is not obvious at all). I suggest replacing with cross-component and/or multi-scales as needed, which are more standard definitions.

RE: Good point! Please see the new title.

3. L32: "two" and not "multiple"

RE: Corrected. Thanks. Please see **L32** in the revision.

4. L34: The sentence is not clear. "printed in variability of the rebuilt GMOC" is hard to understand and needs rephrasing.

RE: Rephrased. Thanks. Please see **L34** in the revision.

5. L39-41 I suggest expanding the discussion on the importance of GMOC on the global climate, as only a couple of lines is not enough to set up the motivation of the work.

RE: Excellent comment! More discussions are added in the revision. Please see **L39-47** in the revision.

6. Additionally, still in the initial methodological discussion, inverse methods (box models, etc.) can be used to infer GMOC, together with other indirect methods, and need to be mentioned and cited.

RE: Same as point 5, excellent comment! More discussions are added in the revision. Please see **L42-47** in the revision.

7. L44 "multi-spheric" again unclear

RE: Done. Please see **L50**. Thanks.

8. L52 "especially in the deep ocean", as bias does not only occur at depth

RE: Done. Please see **L56**.Thanks.

9. Many references (eg, 24, etc.) refer to AMOC and not GMOC, but the authors use them for GMOC. I suggest carefully checking in order not to consider for GMOC works that focus on AMOC.

RE: Thanks a lot for this good suggestion! The related places are reworded accordingly. AMOC is a key component of GMOC, and its dynamic mechanisms and research methods inform GMOC studies. We have carefully rechecked all AMOC-related references in the revised manuscript, confirmed their applicability to GMOC research, and retained those with clear reference value.

10 L64 Why is "observations" quoted?

RE: Clarified. Please see **L62**. Thanks.

11. The results about the convergence of the two reanalyses in terms of GMOC are interesting. However, the two historical simulations from CESM and CM2 appear quite close as well in the

way they reproduce the GMOC. It would be important to understand whether a completely different representation of the GMOC from a data-unaware coupled model would converge, with CDA, to the same reanalyzed GMOC. I understand this is difficult to prove in practice, but if the authors have any comments or speculation, it would be interesting.

RE: Very excellent comment! It is true that due to common deficits on dynamics (coarsely-discretized fluid units and quite smooth topography etc.) approximation on physics (sub-grid processes' parameterizations, for instance), the mean state of GMOCs in data-unaware coupled models have similar distorted features. We change figure presentation and discuss this in the revision. Please see **L118-127 in main text** and **L195-207 in SI** of the revision.

12. The choice of ocean reanalyses used in Fig 1 seems arbitrary; either there is a very strong justification for including ORA and SODA, or I suggest to include (at least here) also other reanalyses, such as GLORYS12, ECCO, CGLORS, MOVE, etc., which in turn would make the assessment more complete. There was also a coupled reanalysis, CERA. produced by ECMWF that would offer another comparison dataset closer to the formulation of the CDA here (although CERA was probably not built for low-frequency variability studies): I think it could be at least mentioned and shown, if data are available. The manuscript will become of broader interest if these datasets are compared.

RE: Very excellent comment! Mainly based on the time length of datasets, we used ORA and SODA. In the revision, we add more ocean reanalyses (ECCO4 and GLORYS12) as suggested by the reviewer although their time spans are relatively short. Indeed, now the comparison and analyses are more convincing and robust. Please see the new **Supplementary Figs. 5-7** and corresponding analyses and discussions on **L134, L172, L187-190 in SI** of the revision. Due to the unavailability of CERA data, the corresponding results are not presented in this manuscript. Nevertheless, the references to CERA have been included in the text, please see **L52, L276, and L430**.

13. The other major point is about the reasons for the convergence. The authors are confident about the fact that the CDA approach leads to convergent and improved representation of GMOC and underlying processes. However, while they show many diagnostics that suggest so, this is not fully conclusive. For instance, when compared to the ocean reanalysis like ORAS5, the impact could be due to the i) use of coupled model instead of ocean-only; ii) use of coupled data assimilation; iii) use of surface pressure observations in the coupled DA scheme (are these crucial or not?); iv) use of multiple scales for the error covariances. While disentangling the merits of each enhancement compared to standard ocean reanalyses might be difficult, it will be important if the authors can discuss the relative merits of these CDA ingredients, as they

certainly performed many preliminary experiments to reach the final configuration.

RE: Very excellent comment! Following the reviewer's suggestion, in the revision we performed additional diagnostics and analyses to try to clarify the reason of why our multiscale CDA achieves the improved GMOC representation. For the use of coupled model instead of ocean-only, please see **L191-194 in SI** of the revision. For the use of coupled data assimilation, please see **L101-128 in SI** of the revision. For the use of multiscale data-constraints, please see **L67-71 and L247-251 in SI** of the revision.

14. The results' section is quite convincing, simple, and clear. I found, however, the discussion related to Figure 4 slightly disconnected, or perhaps to move at the beginning (e.g., as a proof of the reliability of the two reanalyses). Alternatively, the authors can improve the initial part of the section to make a clearer link and motivation with the previous analyses.

RE: Very excellent comment! We reorganize the presentation to make the story more silky. Please see the new title and the new organization of the revised manuscript.

15. Methods: The bias adjustment is a quite standard and old method (strong relaxation to climatology, even with a 30d strength at the bottom), so I would not emphasize its sophistication.

RE: Agree! We changed the related statements. Please see **L265-269** in the revision.

16. L435: What do you mean by pressure data from ERA5? Are you assimilating gridded data from the reanalyses or raw observations?

RE: Clarified. We assimilate gridded surface pressure from ERA5. Please see **L273** in the revision.

17. L486: Is the 0.1 mm/s removal due to the use of the bottom as no motion level instead of the usual 1000 m- 2000 m of depth?

RE: Clarified. Please see **L260-261 in the SI** revision. A small, spatially uniform velocity of O(0.1mm s−1) was subsequently removed from the geostrophic velocity field (Armin and Detlef, 2008, JPO). This step was necessary to eliminate a spurious, basin-scale numerical drift introduced during the calculation, ensuring that the net meridional transport across the basin is zero, as required by mass conservation in a closed ocean basin.

**Reviewer #1:**

I have carefully examined the revised manuscript and the authors' responses to my previous comments. The authors have made a significant effort to revise the manuscript. The title has been clarified, the figures have been substantially improved, and additional analyses and diagnostics have been added. I appreciate the authors' careful responses and the improvements in the overall presentation. However, several of my main concerns remain only partially addressed, and in my opinion further clarification and analysis are still required before the manuscript can be considered for publication.

RE: Thanks a lot for this reviewer's encouragement! We have fully addressed each comment in the revision. Please see the following p2p responses.

First, the central claim of the manuscript is that the CDA substantially improves the representation of the GMOC. In the revised manuscript the authors place greater emphasis on multidecadal variability rather than on the mean-state performance. While this shift in emphasis is understandable, the original concern remains: the CDA simulations do not clearly outperform the historical simulations in representing overturning circulation, particularly in the deep ocean. The manuscript should provide a clearer and more quantitative demonstration of the specific advantages of CDA relative to free model simulations.

RE: We thank the reviewer for this comment. To clearly and quantitatively demonstrate the advantages of CDA over free simulations—particularly in the deep ocean—we have added three new analyses:
1) Potential density structures (**Supplementary Fig. 11** and **L262-277** in the SI);
2) Geostrophic GMOC diagnostics (**Supplementary Fig. 14a-b** and **L316-324** in the SI);
3) Evaluation of deep AMOC transport (**Supplementary Fig. 14c-d** and **L325-332** in the SI).
These results are also summarized in the main text (see **L89-92**).

Second, the integration length of the coupled simulations (approximately seven decades) remains relatively short compared with the adjustment timescale of the deep overturning circulation. The authors argue that data assimilation accelerates the spin-up of the ocean state, but additional evidence is needed to demonstrate that the deep overturning structure is sufficiently equilibrated to support the conclusions drawn in the manuscript.

RE: Thanks a lot for this good point! To address this concern, we use the deep MOC index to explicitly illustrate the ocean spin-up evolution, as presented in **Supplementary Fig. 5** and add more analyses in **Supplementary Text 1d** (**L136-147**) in SI, and summarize in the main text in **L295-299**.

Third, the role of the deep-ocean relaxation toward climatology still requires clarification. Because a strong relaxation is imposed below 1000 m, it is difficult to assess how much of the apparent improvement in deep stratification arises from the CDA framework itself versus the imposed climatological constraint. Some additional discussion or sensitivity analysis would help clarify this issue.

RE: We thank the reviewer for raising this important issue. To clarify the respective roles of CDA and deep-ocean relaxation, we revisit twin-experiment studies (see following **Fig. S1, S2,** from Lu et al., 2020), which show that applying CDA alone distorts the AMOC. This is because ocean observations are mostly concentrated above 2000 m (and mainly above 500 m), leaving the deep ocean poorly constrained, leading to inconsistent upper-deep stratification.

We then conducted an additional experiment with only deep-ocean restoring (1945–2022) in CM2. This deep-restoring-only run produces a biased mean GMOC and unrealistic variability (see the following **Fig. S3**). Together, these results demonstrate that neither CDA alone nor deep restoring alone suffices for the ocean stratification.

A balanced and coherent ocean stratification, which is critical for reconstructing the meridional overturning circulation, can only be obtained by constraining deep-ocean biases via climatological relaxation and limiting upper-ocean deviations with observational information concurrently. This discussion has been added to the main text (see **L283-286**).

**Figs. S1–S3** are shown below.

[Figure redacted]

**Figure S1**. (a) The CDA0 (CDA only experiment) AMOC mean state (1981–87) and (b) the climatology and (c) the anomaly time series (six-point smoothing) of AMOC indices produced by CM2.1 CDA0 (red) and CTL (blue) as well as the CESM "TRUTH" run (black) *(From Lu et al., 2020, Fig. 7)*.

[Figure redacted]

**Figure S2**. As in Figure S2, but for CDA3 (CDA + deep ocean restoring experiment) *(From Lu et al., 2020, Fig. 14)*.

Lu, L., Zhang, S., Yeager, S. G., Danabasoglu, G., Chang, P., Wu, L., Lin, X., Rosati, A., & Lu, F. Impact of coherent ocean stratification on AMOC reconstruction by coupled data assimilation with a biased model. J. Clim., 3, 7319–7334 (2020). https://doi.org/10.1175/JCLI-D-19-0735.1 (reference 18 in the main text)

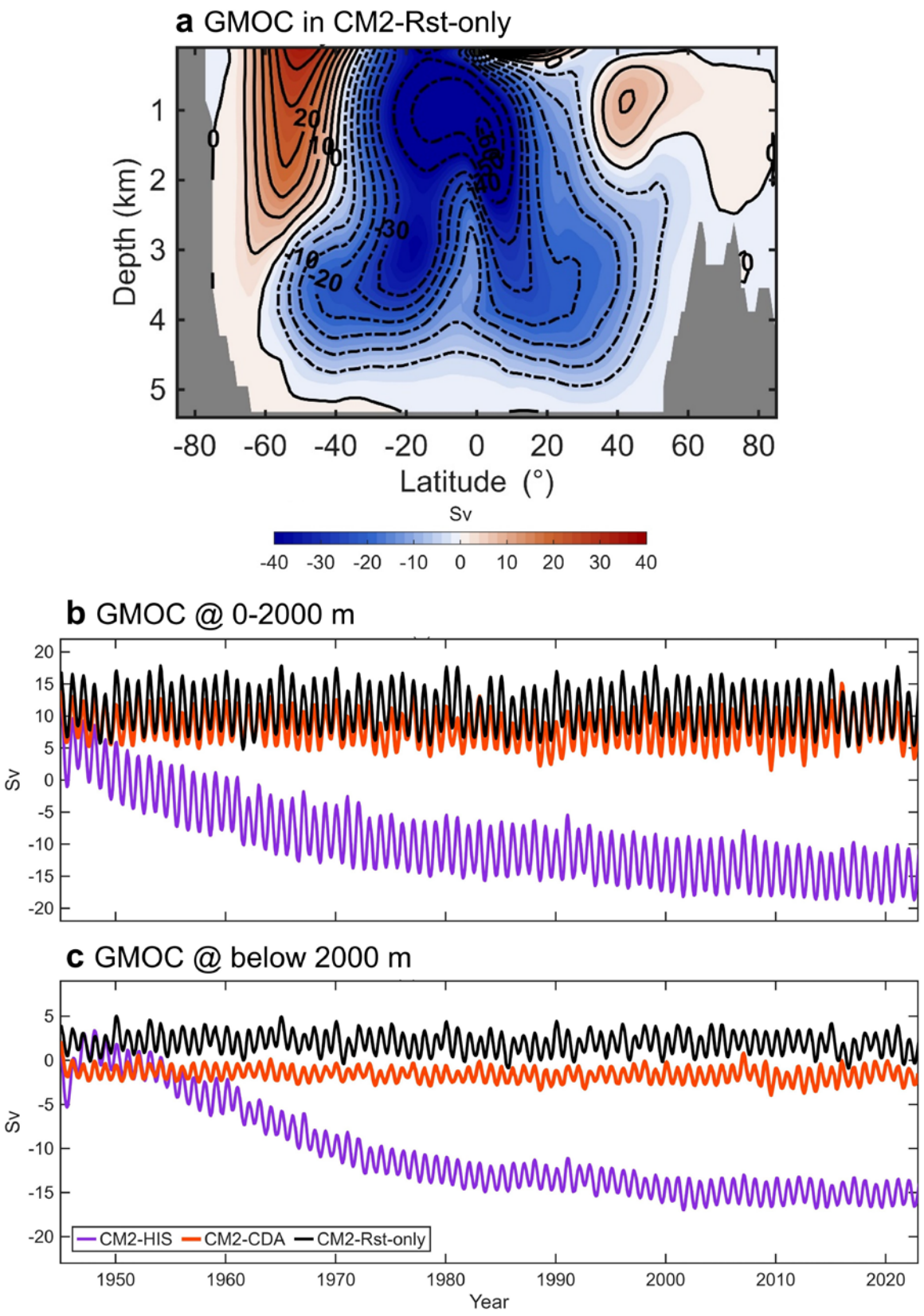


**Figure S3**. a) GMOC mean for the CM2 restoring only experiment (CM2-Rst-only); b–c) Time series of the averaged GMOC over b) 0–2000 m depth and c) below 2000 m for CM2-HIS (black), CM2-CDA (red) and CM2-Rst-only (purple).

Finally, although additional diagnostics have been introduced, the dynamical interpretation of GMOC variability remains somewhat limited. The analysis still relies primarily on correlations among indices. A clearer mechanistic interpretation—possibly through more explicit overturning or transport diagnostics—would strengthen the scientific contribution of the study.

RE: We thank the reviewer for the helpful suggestion. We have now added a more explicit mechanistic interpretation of the GMOC variability by analyzing Southern Hemisphere wind-driven transports, as well as diapycnal mixing-induced transports in the Northern Hemisphere and low latitudes, with quantitative comparisons against ERA5 and EN4. Please see **Supplementary Fig. 25** and **Supplementary Text 3c** (**L449-466**). In addition, surface buoyancy forcing and eddy-induced transports are already examined in **Fig. 4**. Due to the lack of sufficient observational products for comparison, we focused our analysis on the response to volcanic events in the CDA.

In summary, the manuscript has improved in several aspects, but further work is needed to convincingly demonstrate the advantages of the CDA approach and to clarify the dynamical interpretation of the results.

RE: Thanks for your thorough examination! Your constructive suggestions have substantially improved the quality of our manuscript.

**Reviewer #2:**

The authors have revised the manuscript in a satisfactory way, addressing all my previous concerns, and I am therefore happy with the revision.

There is only one remaining point in my opinion (I should have mentioned it earlier, though): one important claim of the authors is the improvement borne by CDA, which is verified, for instance, in the turbulent fluxes. However, these are verified against ERA5. I think that while ERA5 is a good reference for the wind work, pressure, and all other parameters investigated, I doubt this can be a good reference for air-sea turbulent fluxes. I then suggest reporting any literature speculating on the accuracy of ERA5 turbulent fluxes and mentioning its limitations (here, what is important is the increase in consistency), and ideally, comparing with other independent datasets not based on models.

RE: We thank the reviewer for raising this important point. To address the concern about using ERA5 as a reference for turbulent fluxes, we have now included two purely observation-based products, NOCS (National Oceanography Centre Southampton) and OSB-CDR (SeaFlux Ocean Surface Bundle Climate Data Record) (see **L312-314** in the main text) for comparison (see new **Supplementary Fig. 3**, **L113-118** and **L123-128** in SI). A discussion of the limitations of both ERA5 and the observational products has also been added (**L128-135** in SI).

Manuscript ID: NCOMMS-25-3708-T

Title: **Organization of Historical Oceanic Overturnings on Cross-Sphere Climate Signals**

Authors: **Jiang et al.**

Originality: **Fair**
Technical Quality: **Bad**
Clarity of Presentation: **Not good**
Significance: **insignificant**

Recommendation: **Rejection**

The authors present two 80-year coupled reanalyses (CESM-CDA and CM2-CDA) produced with a multiscale coupled-data-assimilation (CDA) scheme that jointly ingests atmospheric surface pressure, SST and subsurface temperature/salinity (1945-present). They claim the new reconstructions (i) converge on a common global meridional overturning circulation (GMOC) mean state, (ii) reproduce multiscale GMOC variability—including fingerprints of major volcanic eruptions—and (iii) will underpin "AI coupled big models." The work addresses the pressing need for dynamically consistent long-term Earth-system reanalyses, and the technical effort behind two independent CDA systems is impressive.

The CDA effort is valuable, but the manuscript in its present form suffers from the following fundamental weaknesses: the title is misleading, figures fall short of publication quality, CDA fails to improve the key diagnostic, the runs are not spun up for AMOC equilibrium, and no new scientific insight is provided, the manuscript should be rejected.

**Major Comments:**

1. Title–content mismatch. The manuscript's title—*"Organization of Historical Oceanic Overturnings on Cross-Sphere Climate Signals"*—does not reflect what is actually delivered. The paper is essentially a performance assessment of coupled-model hindcasts with and without coupled data assimilation (CDA). Terms such as "organization," "cross-sphere," and "climate signals" are never defined or demonstrated in the text, leaving the reader confused about the study's scope and novelty.

2. Poor figure quality. Many figures are pixelated, employ non-uniform "rainbow" colour scales, and use fonts below 6 pt, making key details illegible. Several panels lack units and consistent axis limits. The visual presentation is well below the standards expected for *Nature Communications*.
3. CDA does not outperform the historical run for overturning circulation. In Figure 1 the global/Atlantic meridional overturning circulation produced by the CDA integrations does **not** outperform the corresponding historical (HIS) simulations; in some depth ranges the skill is even worse. This directly contradicts the manuscript's central claim that CDA "substantially improves" overturning realism.
4. Insufficient integration length for AMOC adjustment. The coupled integrations span approximately 72 years (1950–2022). For the AMOC this is far shorter than the adjustment time-scale required for the deep limb to reach quasi-equilibrium. Consequently, any diagnosis of heat–salt overturning variability is premature; only upper-ocean thermodynamic metrics can be considered marginally adequate.
5. No genuine scientific discovery. The manuscript is essentially a model-to-model comparison exercise. Beyond reporting performance metrics, it offers no new physical insight into ocean dynamics, climate interactions or predictability. Without a substantive scientific question and discovery, the work does not meet the novelty threshold for this journal.
6. Bias-correction strategy may undermine "deep-ocean realism". A strong depth-dependent relaxation to World Ocean Atlas climatology is imposed below 1000 m (Methods, lines 423-431). This nudging dominates the deep stratification that is subsequently highlighted as a CDA "achievement" (Fig. 1g-n). The authors should clarify how much of the apparent improvement arises from the bias-relaxation rather than true multiscale CDA; include sensitivity tests without deep nudging.
7. Causal attribution for GMOC variability is largely descriptive. The manuscript relies on correlations among synthetic indices (GMOC, NADW thickness, ACC-RC, εm) but lacks closed budget analyses. No meridional heat- or salt-transport diagnostics, streamwise potential-vorticity budgets, or overturning decompositions (isopycnal vs. diapycnal) are presented. The authors should supply full overturning budgets (wind-driven Ekman, eddy, diapycnal mixing, surface buoyancy forcing) for at least two key decades; quantify the contribution of each term to GMOC tendencies.
8. Overstatement of "AI big-model" relevance. The authors claim that the reanalyses will "give birth to Artificial Intelligence coupled big models" (abstract, lines 34-36) are marketing language

without concrete demonstration. Either provide a proof-of-concept AI application (e.g., training a neural emulator on the CDA fields with performance metrics) or tone down the statement.

Manuscript ID: NCOMMS-25-3708-T

Title: **The Coupled Reanalysis Global Meridional Overturning Circulation Imprinted by Historic Volcanic Eruptions**

Authors: **Jiang et al.**

Originality: **Fair**
Technical Quality: **Fair**
Clarity of Presentation: **Fair**
Significance: **Fair**

Recommendation: **Major revision**

I have carefully examined the revised manuscript and the authors' responses to my previous comments.

The authors have made a significant effort to revise the manuscript. The title has been clarified, the figures have been substantially improved, and additional analyses and diagnostics have been added. I appreciate the authors' careful responses and the improvements in the overall presentation.

However, several of my main concerns remain only partially addressed, and in my opinion further clarification and analysis are still required before the manuscript can be considered for publication.

First, the central claim of the manuscript is that the CDA substantially improves the representation of the GMOC. In the revised manuscript the authors place greater emphasis on multidecadal variability rather than on the mean-state performance. While this shift in emphasis is understandable, the original concern remains: the CDA simulations do not clearly outperform the historical simulations in representing overturning circulation, particularly in the deep ocean. The manuscript should provide a clearer and more quantitative demonstration of the specific advantages of CDA relative to free model simulations.

Second, the integration length of the coupled simulations (approximately seven decades) remains relatively short compared with the adjustment timescale of the deep overturning circulation. The authors argue that data assimilation accelerates the spin-up of the ocean state, but additional evidence is needed to demonstrate that the deep overturning structure is sufficiently equilibrated to support the conclusions drawn in the manuscript.

Third, the role of the deep-ocean relaxation toward climatology still requires clarification. Because a strong relaxation is imposed below 1000 m, it is difficult to assess how much of the apparent improvement in deep stratification arises from the CDA framework itself versus the imposed climatological constraint. Some additional discussion or sensitivity analysis would help clarify this issue.

Finally, although additional diagnostics have been introduced, the dynamical interpretation of GMOC variability remains somewhat limited. The analysis still relies primarily on correlations among indices. A clearer mechanistic interpretation—possibly through more explicit overturning or transport diagnostics—would strengthen the scientific contribution of the study.

In summary, the manuscript has improved in several aspects, but further work is needed to convincingly demonstrate the advantages of the CDA approach and to clarify the dynamical interpretation of the results.
